# Investigation of de novo mutations in a schizophrenia case-parent trio by induced pluripotent stem cell-based in vitro disease modeling: convergence of schizophrenia- and autism-related cellular phenotypes

**DOI:** 10.1186/s13287-020-01980-5

**Published:** 2020-11-27

**Authors:** Edit Hathy, Eszter Szabó, Nóra Varga, Zsuzsa Erdei, Csongor Tordai, Boróka Czehlár, Máté Baradits, Bálint Jezsó, Júlia Koller, László Nagy, Mária Judit Molnár, László Homolya, Zsófia Nemoda, Ágota Apáti, János M. Réthelyi

**Affiliations:** 1 National Brain Research Project (NAP) Molecular Psychiatry Research Group, Hungarian Academy of Sciences and Semmelweis University, Budapest, Hungary; 2 Molecular Cell Biology Research Group, Institute of Enzymology, Research Center for Natural Sciences, 1117 Magyar tudósok körútja 2, Budapest, Hungary; 3 Institute of Rare Disorders and Genomic Medicine, Semmelweis University, Budapest, Hungary; 4 Department of Biochemistry and Molecular Biology, Faculty of Medicine, University of Debrecen, Debrecen, Hungary; 5 Department of Medical Chemistry, Molecular Biology and Pathobiochemistry, Semmelweis University, Budapest, Hungary; 6 Department of Psychiatry and Psychotherapy, Semmelweis University, Balassa utca 6, Budapest, 1083 Hungary

**Keywords:** Schizophrenia, Autism, DNM, KHSRP, LRRC7, iPSC, Disease-modeling, RNASeq, Glutamate, Proliferation, Mitochondrial function

## Abstract

**Background:**

De novo mutations (DNMs) have been implicated in the etiology of schizophrenia (SZ), a chronic debilitating psychiatric disorder characterized by hallucinations, delusions, cognitive dysfunction, and decreased community functioning. Several DNMs have been identified by examining SZ cases and their unaffected parents; however, in most cases, the biological significance of these mutations remains elusive. To overcome this limitation, we have developed an approach of using induced pluripotent stem cell (iPSC) lines from each member of a SZ case-parent trio, in order to investigate the effects of DNMs in cellular progenies of interest, particularly in dentate gyrus neuronal progenitors.

**Methods:**

We identified a male SZ patient characterized by early disease onset and negative symptoms, who is a carrier of 3 non-synonymous DNMs in genes LRRC7, KHSRP, and KIR2DL1. iPSC lines were generated from his and his parents’ peripheral blood mononuclear cells using Sendai virus-based reprogramming and differentiated into neuronal progenitor cells (NPCs) and hippocampal dentate gyrus granule cells. We used RNASeq to explore transcriptomic differences and calcium (Ca^2+^) imaging, cell proliferation, migration, oxidative stress, and mitochondrial assays to characterize the investigated NPC lines.

**Results:**

NPCs derived from the SZ patient exhibited transcriptomic differences related to Wnt signaling, neuronal differentiation, axonal guidance and synaptic function, and decreased Ca^2+^ reactivity to glutamate. Moreover, we could observe increased cellular proliferation and alterations in mitochondrial quantity and morphology.

**Conclusions:**

The approach of reprograming case-parent trios represents an opportunity for investigating the molecular effects of disease-causing mutations and comparing these in cell lines with reduced variation in genetic background. Our results are indicative of a partial overlap between schizophrenia and autism-related phenotypes in the investigated family.

**Limitations:**

Our study investigated only one family; therefore, the generalizability of findings is limited. We could not derive iPSCs from two other siblings to test for possible genetic effects in the family that are not driven by DNMs. The transcriptomic and functional assays were limited to the NPC stage, although these variables should also be investigated at the mature neuronal stage.

## Background

Schizophrenia (SZ) is a chronic, debilitating psychiatric disorder characterized by positive and negative symptoms, such as hallucinations, delusions, and blunted affect as well as alogia, avolition, social withdrawal, cognitive dysfunction, and decreased community functioning. Despite considerable development in pharmacological and psychosocial treatment possibilities, one third of patients does not respond to existing interventions and demonstrates a poor outcome [1, 2]. The molecular and neurobiological processes underlying this disorder are poorly understood and possibly quite heterogeneous. Therefore, based on the limited knowledge of disease pathways, new research approaches are needed to improve our insight of the etiology of SZ.

Induced pluripotent stem cell (iPSC) based disease modeling represents a new avenue of research in the investigation of neuropsychiatric disorders that has been successfully used to study molecular disease pathways in this nosologic group. Briefly, the approach takes advantage of somatic cell reprogramming, which results in iPSC lines that capture genetic variants carried by diseased individuals. After reprogramming, the effects of these putative disease-causing variants can be investigated on various molecular and functional phenotypes in neuronal cell types of interest, by means of targeted differentiation protocols [3–5].

Previous efforts to elucidate the genetic background of SZ and autism have identified both common and rare variants that show association with disease status or various disease-related phenotypes [6]. Interestingly, both common and rare disease causing genetic variants, i.e., single-nucleotide polymorphisms (SNPs) and single-nucleotide variants (SNVs) or gene copy number variants (CNVs), respectively, show a considerable degree of overlap between SZ and other psychiatric disorders, including autism spectrum disorder (ASD) [7–11]. De novo mutations (DNMs) represent a subclass of SNVs, in cases where the rare mutations appear unprecedentedly, de novo in a given generation, and contribute a unique source of genetic variation that plays a role in both autism and SZ. Genetic research focusing on DNMs highlights the importance of variants in evolutionarily conserved, mutation-intolerant genes that perturb downstream essential molecular pathways in neurons [12].

The effects of SZ-associated genetic variants have been investigated previously in iPSCs both directly and indirectly, i.e., by using cell lines with known genetic variants [13] or studying cell lines derived from sporadic SZ cases, without identified genetic alterations [14]. The field of iPSC-based disease modeling in SZ was founded by the seminal paper of Brennand et al. [14] that has been followed by a series of studies looking at different aspects of SZ-related in vitro phenotypes. Reviews comparing these studies have been looking for overlapping findings between these results and identified glutamatergic synaptic dysfunction, Wnt signaling, and increased oxidative stress as potentially significant in vitro phenotypes for SZ [15, 16]. In comparison, in iPSC-based disease modeling studies of ASD, the most consistent phenotypes are increased proliferation and apoptosis of NPCs, with altered expression of genes responsible for these processes, as well as higher number of interneurons, decreased synaptic activity, and dysregulation of neuronal differentiation programs [17, 18].

To unravel the biological functions of SZ DNMs in a cellular context, one possible approach is the investigation of isogenic cell lines after introduction or correction of a DNM by genome editing. This approach has been used for SZ research in previous studies; however, they focused on common SZ risk variants [19, 20]. It was shown that a single SNP can give rise to differential neuronal phenotypes and establish expression quantitative trait loci [19]. Another potential way of investigation is to use case-parent trios and generate iPSC lines from the proband and both parents in order to simulate familial risk and the effects of the de novo mutation. This approach has been used recently both in SZ and ASD [21, 22].

Based on our previous exome-sequencing results and bioinformatics analyses, we focused on downstream effects of 3 genes, LRRC7, KHSRP, and KIR2DL1, in which DNMs were identified in the SZ patient. The K-homology type splicing regulatory protein (KHSRP) is a RNA-binding protein that modulates RNA life and gene expression at different levels, including mRNA decay, miRNA biogenesis, and interaction with lncRNAs [23]. It is localized to the nucleus and cytoplasmic granules. The biological significance of this gene has been shown in cell fate determination, immune response, neuronal differentiation, and neurite outgrowth. Based on its diverse roles as an RNA-binding protein in neurons, it has been suggested to play an etiologic role in several neuropsychiatric disorders [24]. KHSRP has also been identified as a potential SZ risk gene in a study based on transcriptomic differences in circulating white blood cells [25].

The leucine-rich repeat containing 7 (LRRC7) gene encodes densin-180, a postsynaptic density protein in glutamatergic synapses. In an LRRC7 KO animal model, the lack of this protein resulted in decreased dendritic spine number and altered behavioral phenotypes [26]. LRRC7 was also associated with emotional dysregulation and autistic traits, since LRRC7 KO mice had inappropriate juvenile aggressive behavior and significant anxiety-like behavior and social dysfunction in adulthood [25]. The killer cell immunoglobulin-like receptor 2DL1 (KIR2DL1) gene encodes killer cell immunoglobulin-like receptors that are transmembrane glycoproteins expressed by natural killer cells and certain T cells, which plays an important role in regulating immune responses [27]. Due to the gene’s tissue-specific expression patterns, this DNM was not expected to play any role in neuronal differentiation processes or neuronal functions and, therefore, not taken forward for characterization in this study.

In summary, we aimed to investigate the biological effects of DNMs identified in a SZ patient using iPSC-based disease-modeling applied to all members of a case-parent trio. After generation of iPSCs and neuronal differentiation into hippocampal dentate gyrus granule cells, we sought transcriptomic alterations and tested specific cellular phenotypes in vitro, potentially characteristic to SZ, according to previous studies. Based on mutation prediction tools, we hypothesized transcriptomic and functional alterations related both to SZ and previously known biological functions of KHSRP, i.e., neuronal differentiation and neurite outgrowth. As presented hereafter, we were able to uncover marked transcriptomic differences and subtle physiological alterations in the proband-derived neuronal progenitor cells related to neuronal progenitor proliferation, calcium-signaling, and mitochondrial function.

## Methods

### Subject selection and characterization, identification of de novo mutations

We used cell lines derived from 3 human subjects (SZ-HU-PROB, SZ-HU-MO, SZ-HU-FA), a SZ patient and his parents, i.e., a case-parent trio for all experiments (Table 1). (The patient’s two siblings, an unaffected brother and a sister diagnosed with bipolar affective disorder, were not included neither in the genetic analysis, nor the subsequent cell reprogramming.) The patient diagnosed with SZ was selected from 16 similar trios, based on DNMs identified by exome sequencing. Briefly, the ExomeSeq analysis was carried out after 100 bp paired ended sequencing that was run on the Illumina HiScan (TM) SQ platform and resulted in 50 M reads on average per sample. Results were analyzed by a standard bioinformatics pipeline. The identified DNMs were validated by Sanger sequencing. The SZ patient (SZ-HU-PROB) is a carrier of 3 missense DNMs in genes KHSRP (19:6416869C>A), LRRC7 (1:70505093G>A), and KIR2DL1 (19:55286658A>T). Of these, SIFT mutation algorithm predicts the KHSRP mutation as deleterious (Table 2). None of these DNMs have been reported previously to the ClinVar database [28]. An unrelated healthy iPSC line (UCB2) derived from umbilical cord PBMCs was used as a control in the functional assays.

**Table 1:** Demographic and clinical data of the investigated case-parent trio, the extended family, and the unrelated healthy control individual

Subject	Sex	Age	Medical history	Code
**Father**	M	59	No psychiatric treatment or other major somatic disorders.	iPSC-SZ-HU-FA 1
**Mother**	F	55	No psychiatric treatment or other major somatic disorders.	iPSC-SZ-HU-MO 1 and 2
**Proband (son)**	M	24	Diagnosed with schizophrenia at the age of 17. During the past 10 years had 3 hospitalizations, receives clozapine treatment. Predominantly negative symptoms, as measured by PANSS.	iPSC-SZ-HU-PROB 1 and 2
**Unaffected older sibling**	M	28	No psychiatric treatment or other major somatic disorders.	–
**Younger sibling**	F	21	Diagnosed with bipolar affective disorder at age of 18 after suicidal attempt. Receives lithium and olanzapine treatment.	–

**Table 2:** Description of the identified DNMs in proband SZ-HU-PROB

Genomic position	1:70505093G>A	19:6416869C>A	19:55286658A>T
**Gene**	LRRC7	KHSRP	KIR2DL1
**Variation type**	Missense	Missense	Missense
**Amino acid change**	Val1158Ile	Gly403Cys	Thr138Ser
**Conservation of nucleotide**	Weak	High	Not conserved
**Conservation of amino acid**	High	Moderate	Weak
**SIFT**	Tolerated (score 0.37, median 4.32)	Deleterious (score 0.03, median 3.54)	Tolerated (score 0.73, median 3.01)
**Mutation taster**	Polymorphism (*p* value 0.999)	Disease causing (*p* value 0.996)	Polymorphism (*p* value 1)

### Generation and characterization of iPSC lines

Blood samples were obtained from trio members after written informed consent. The iPSC generation process and the study were approved by the Human Reproduction Committee of the Hungarian Health Science Council (ETT HRB). The iPSC generation has been presented elsewhere (Hathy E, Szabó E, Vincze K, Haltrich I, Kiss E, Varga N, et al. Generation of multiple iPSC clones from a male schizophrenia patient carrying de novo mutations in genes KHSRP, LRRC7, and KIR2DL1, and his parents, submitted), but we briefly summarize it here as well. Blood was collected directly to cell preparation tubes with sodium heparin (BD Vacutainer CPT, Cat. no.: 362782) to isolate peripheral mononuclear cells (PBMCs) from all samples. PBMCs were cultured for 4 days with daily medium changes at a density of 5 × 10^5^ cells/ml in StemPro®-34 (Thermo Fisher) hematopoietic medium supplemented with 2 mM L-Glutamine and cytokines at the following final concentrations (SCF 100 ng/mL, FLT-3100 ng/mL, IL-3 20 ng/mL, IL-6 20 ng/mL, all from Peprotech). On day 4, PBMCs were transduced with Sendai virus (Fusaki et al., 2009) particles (Thermo Fisher Cytotune 2.0) carrying KOS (hKlf4, hOct3/4, hSox2), hc-Myc, and hKlf4 at MOIs of 5, 5, and 3, respectively. After addition of the virus particles, samples were centrifuged in a 12-well plate for 90 min at 2250 rpm and incubated at 37 °C overnight. After changing the medium, the cells were maintained for additional 2 days, then transferred to culturing dishes previously seeded with mouse embryonic fibroblasts (MEFs) and cultured in StemPro®-34 medium without cytokines. Six days after transduction, the culturing medium over the cells was gradually changed to HUES medium (KO-DMEM, supplemented with 15% KO Serum Replacement (Thermo Fisher), 100 mM glutamine, 1% nonessential amino acids, 0.1 mM β-Mercaptoethanol, and 4 ng/ml recombinant human basic fibroblast growth factor, bFGF).

Fourteen to 18 days after transduction, individual iPSC colonies emerging were mechanically isolated and transferred to MEFs to generate clones. To ensure virus clearance and monitor stability, the clones were repeatedly passaged and expanded up to p10 using trypsin. Heat treatment at 38.5 °C was used between passage p4-p8 to take advantage of the heat sensitivity of virus particles. The pluripotent state and trilineage differentiation capacity of iPSCs were tested by quantitative PCR and ICC, using established methods [29]. The investigated KHSRP and LRRC7 DNMs were confirmed in the iPSCs with Sanger sequencing. iPSC UCB2 was generated from umbilical cord PBMCs using Sendai virus reprogramming, independently from the other trio iPSC lines, and characterized (Supplementary Fig. 1).

### Cell culturing and neuronal differentiation

Neural progenitor cells (NPCs) were differentiated from iPSC lines SZ-HU-PROB 1 and 2, SZ-HU-MO 1 and 2, SZ-HU-FA 1, and UCB2 (unrelated healthy control) as described previously [30, 31]. Briefly, before starting differentiation, iPSC cells were transferred to Matrigel (Corning, NY, USA) coated plates in mTeSR medium (Stemcell Technologies, Vancouver, Canada) and were cultured to high density. On day 1, the cells were detached with collagenase (Thermo Fisher Scientific, MA, USA) and transferred to ultra-low attachment plates (Nalgene Nunc International, NY, USA). After embryoid body (EB) formation, the medium was changed to DMEM/F-12, GlutaMAX™ (Thermo Fisher Scientific, MA, USA) medium supplemented with N2/B27 (Thermo Fisher Scientific, MA, USA) and anticaudalizing agents (500 ng/ml Noggin (Thermo Fisher Scientific, MA, USA), 500 ng/ml DKK1 (PeproTech, NJ, USA), 1 μg/ml Cyclopamine (Merck, Darmstadt, Germany), and 4 μg/ml SB431542 (Sigma, MO, USA)). The treatment was repeated every other day. On day 20, the EBs were moved to a poly-ornithine (Sigma, MO, USA)/laminin (Thermo Fisher Scientific, MA, USA) coated plate to support further differentiation in adherent conditions. On day 27 or later, manually picked rosettes were dissociated by Accutase (Thermo Fisher Scientific, MA, USA) and re-seeded onto a new poly-ornithine/laminin coated plate in DMEM/F-12, GlutaMAX™ N2/B27 medium containing 20 ng/ml FGF2 (Thermo Fisher Scientific, MA, USA) and 1 μg/ml laminin. The attached neural progenitor cells (NPCs) showed uniform morphology after 5 passages. The NPCs between passage p5 and p15 were used for the experiments. For neuronal differentiation experiments, NPCs were further differentiated NPCs were seeded onto poly-ornithine/laminin coated, in eight-well Nunc Lab-Tek II Chambered Coverglass (Nalgene Nunc International, NY, USA) with 1.5 × 103 density in N2/B27 medium supplemented with 200 nM ascorbic acid (Sigma, MO, USA), 20 ng/ml BDNF (PeproTech, NJ, USA), 500 μg/ml cAMP (Sigma, MO, USA), 1 μg/ml Laminin, and 20 ng/ml Wnt3A (Research and Diagnostic Systems Inc., MN, USA). After 3 weeks, Wnt3A was omitted from the medium. The medium was changed every other day.

### Molecular characterization of neuronal progenitors and neurons

For immunofluorescence staining of NPCs and NPC-derived neurons from the case-parent trio and the unrelated control cell line, NPCs were seeded onto poly-ornithine/laminin coated, eight-well chambers and differentiated into DG neurons for 5 weeks as described above. The cells were fixed with 4% paraformaldehyde (Thermo Fisher Scientific, Waltham, MA, USA) in Dulbecco’s modified PBS (DPBS) (Sigma, MO, USA) for 15 min at room temperature. Following washing with DPBS, the samples were blocked for 1 h at room temperature in DPBS containing 2 mg/ml bovine serum albumin (Sigma, MO, USA), 1% fish gelatin (Sigma, MO, USA), 5% goat serum (Sigma, MO, USA), and 0.1% Triton-X 100 (Sigma, MO, USA). The samples were then incubated for 1 h at room temperature with antibodies anti-SOX2 (monoclonal/mouse, 1:20 dilution; MAB2018, R&D Systems, Minneapolis, USA) and anti-Nestin (polyclonal/rabbit, 1:250 dilution; ab92391, Abcam, Cambridge, UK) or for overnight at 4 °C with antibodies anti-PROX1 (polyclonal/rabbit, 1:500 dilution; ab101851, Abcam, Cambridge, UK) and anti-MAP 2 (monoclonal/mouse, 1:500 dilution; M1406, Sigma/Merck, Darmstadt, Germany or polyclonal/rabbit, 1:1000 dilution; ab5622, Millipore, MA, USA). The proteins encoded by the genes of interest harboring DNMs were also investigated by immunofluorescence staining, using anti-KHSRP (1:1000 ab140648, Abcam, Cambridge, UK) and anti-LRRC7 (1:500, HPA005625, Sigma/Merck, Darmstadt, Germany) antibodies. After washing with DPBS, the cells were incubated for 1 h at room temperature with appropriate secondary antibodies: Alexa Fluor 633-conjugated goat anti-mouse IgG or Alexa Fluor 547-conjugated goat anti-rabbit IgG (Thermo Fisher Scientific, MA, USA). The nuclei were counterstained with DAPI (Thermo Fisher Scientific, MA, USA). The stained samples were examined by a Zeiss LSM 900 confocal laser scanning microscope. Fluorescence images were analyzed with the ZEN 3.1 blue edition software. In the confocal images, pseudo-color coding was used for better visualization.

### Gene expression analysis

Total RNA was isolated from NPCs and NPC-derived neurons using TRIzol™ reagent following the manufacturer’s instructions (Thermo Fisher Scientific, MA, USA). cDNA samples were prepared from 0.2 μg total RNA using the Promega Reverse Transcription System Kit (Promega, WI, USA) as specified by the manufacturer. For real-time quantitative PCR, the following Pre-Developed TaqMan® assays were purchased from Thermo Fisher Scientific, MA, USA: NANOG (HS02387400_g1) as undifferentiated stem cell marker; Pax6 (Hs00240871_m1) and SOX2 (HS01053049_s1) as markers of NPC state, as well as NeuroD1 (HS01922955_s1), FoxG1 (HS01850784_s1), and PROX1 (HS00896294_m1), GRIA1 (Hs00181348_m1), and GRIN1 (Hs00609557_m1) as neuronal differentiation specific markers; and RPLP0 (HS99999902_m1) ribosomal protein as endogenous control for quantification. KHSRP (HS01100863_g1) and LRRC7 (HS00363532_m1) expression were examined as well. RT-PCR analyses were carried out using the StepOnePlus™ Real-Time PCR System (Thermo Fisher Scientific, MA, USA), according to the manufacturer’s instructions. The changes in mRNA levels between the examined and control cells were determined by the 2-ΔCt method using RPLP0 (P0) as endogenous control gene. Relative mRNA levels were presented as heatmaps using the mean values of 3 independent experiments.

### RNA-sequencing experiments

RNASeq experiments were carried out at the NPC stage for each cell line in triplicates and quadruplicates. RNA was isolated at 3 or 4 different passages from each NPC line between p7 and p12 to maximize within-clone biological variation. After library preparation samples were sequenced with the Illumina NextSeq 500 technology using 75 bp single-end reads resulting in 25 million reads coverage per sample. Mapping to the *Homo sapiens* (hg19) reference genome was performed using Hisat2 software, after quality control of raw reads with FASTQC and trimming with Trimgalore. Mapped reads were annotated to genes using featureCounts function of the Subread R package. All X and Y chromosome genes were excluded from downstream analyses. To compare transcriptomic differences, we performed differential gene expression (DE) analysis, and (after removing batch effect caused by date of the sequencing) principal component analysis, and cluster analysis using the DESeq2, limma and PCAExplorer R packages, Cluster 3.0, and Treeview, respectively. Differentially expressed genes between two NPC lines were selected if the logarithmic of fold change was greater than 1 and adjusted *p* value smaller than 0.05. Gene Ontology (GO) and pathway analysis of DE genes was performed using WebGestalt [32]. DE genes were considered significantly enriched in GO and PATHWAY terms when their Bonferroni score was less than 0.05. The potential enrichment of DE genes in putative KHSRP target genes was tested with the hypergeometric test. KHSRP target genes were identified using the oRNAment database [33].

### Ca^2+^ signal measurements in NPC cultures

Before the Ca^2+^ measurements NPCs were seeded for 2 days onto eight-well chambers previously coated with poly-ornithine/laminin. NPCs cells were subjected to 1.0 μM Fluo-4 AM (Thermo Fisher Scientific, MA, USA) in a serum free culture medium for 30 min at 37 °C. Extracellular Fluo-4 AM was removed by changing the medium to Hank’s balanced salt solution (Thermo Fisher Scientific, MA, USA) supplemented with 20 mM Hepes (pH = 7.4) (Thermo Fisher Scientific, MA, USA) and 0.9 mM MgCl_2_ (Sigma, MO, USA) (HBSS). The ligand concentrations were chosen according to literature: glutamate (50 μM) (Sigma, MO, USA) and KCl (50 mM). All experiments were performed at 37 °C using ibidi Heating System.

Calcium signal measurements were carried out by acquisition of time lapse sequences of cellular fluorescence images with the FluoViewTiempo (v4.3, Olympus, http://www.olympusmicro.com) software as described earlier [34]. Fluorescence images were acquired between 505 and 525 nm at 488 nm excitation. Image analysis was carried out with ImageJ software. The fluorescence data were normalized to the baseline of glutamate admission with F/FbaseGlu formula, where *F* is the Ca^2+^ signal intensity at a given time point and FbaseGlu is the average of the 20 time points before glutamate admission. Furthermore, we derived the maximum Ca^2+^ signal intensity of 50 time points after glutamate administration (Fmax50). Thus, we obtained the means (and variance) for each subject resulted from all the individual cells (typically 600–750 cells for one clone from 3 independent measurements). For statistical comparison between subjects, we conducted ANCOVA with dependent variable Fmax50/FbaseGlu and used the two clones as covariates. Statistical analysis was conducted with the SAS 9.4 software.

### Functional phenotyping of NPC lines (cell proliferation, migration, neurite outgrowth)

Based on the RNASeq results, we performed targeted functional assessment of NPCs, namely cell proliferation, migration, and neurite outgrowth tests. For the 96-h proliferation assays, 35,000 NPCs were plated per well in a 24-well plate using triplicates for each time point. NPCs were harvested and labeled with the viability marker propidium iodide then measured in a total volume of 120 μl by Attune flow cytometry at 48, 72, and 96 h after. For assessment of NPC migration, we used the scratch or “wound healing” test, as described earlier [35]. Four hundred fifty thousand NPCs were seeded onto poly-ornithine/laminin coated, six-well plates. After reaching confluence, scars were inflicted manually in each well in triplicates. NPC migration was measured at 24 h on bright field micrographs and the wound closure was calculated using ImageJ software. Data represents the results of two independent experiments, each with three replicate scratches and three measurements per scratch. For neurite outgrowth experiments, we used NPCs treated with calcein-AM (10 μM). Three thousand five hundred NPCs were seeded per well in 96-well plates; after 2 h, the cells were treated with para-nitroblebbistatin (10 μM), a known inductor of neurite outgrowth [36] or DMSO. Neurite outgrowth was visualized and measured by fluorescence microscopy using the automated image acquisition and analysis system of the ImageXpress High content screening system. Data from 3 independent experiments were evaluated.

### Oxidative stress and mitochondrial tests

Oxidative stress tolerance was investigated in NPC lines by treatment with CoCl_2_, a well-known hypoxia inducer and reoxygenation [37]. Cell lines were plated at a number of 35,000 cells/well in poly-ornithine/laminin coated 48-well plates using triplicates. Medium was replaced after reaching confluence with CoCl_2_ containing media at two different concentrations (control = 0 μM, 125 μM, 250 μM). After 24 h, the medium was changed to normal medium (reoxygenation). Two days after reoxygenation, viability was measured using the PrestoBlue dye and the fluorescent signal intensity was measured by Enspire Multimode plate reader (Perkin Elmer). Data from 5 independent experiments, each performed with three technical parallels, were analyzed.

NPCs were characterized by their baseline levels of reactive oxygen species (ROS) using the CellROX kit (Thermo Fischer Scientific, Cat. Number: C10444). NPCs were dissociated by Accutase and then 200,000 cells were incubated/treated with CellROX at 500x dilution for 30 min at 37 °C. After the incubation period, cells were washed three times with 1x PBS. Subsequently, the mean fluorescence signal of labeled cells was measured and compared by flow cytometry (Attune NxT Flow Cytometer, ThermoFisher), and the dead cells were gated out by using propidium iodide staining. Data from three independent experiments were evaluated.

Mitochondrial function in NPCs was examined by Mitotracker staining and transmission electron microscopy. The Mitotracker Red dye (MitoTracker™ Red CMXRos, cat. Number: M7512) was applied on NPC cultures after reaching confluence at a concentration of 250 nM. After 30 min incubation at 37 °C, the dye was washed with DPBS, and the NPCs were fixed by 4% PFA for 15 min at RT. Fluorescent signals were acquired by confocal microscopy (Zeiss LSM 900). Images from 3 independent experiments were evaluated with the NIH Image J software by comparing total fluorescence intensity per nucleus. For transmission electron microscopy, briefly, NPCs were dissociated manually or enzymatically with Accutase; the samples were fixed with solution containing 3.2% PFA, 0.2% glutaraldehyde, 1% sucrose, and 40 mM CaCl_2_ in 0.1 M cacodylate buffer. Next, samples were post-fixed with 1% ferrocyanide-reduced osmium tetroxide and dehydrated using a graded ethanol series, and then embedded in Spurr low viscosity epoxy resin medium. Ultrathin sections were collected on Formvar (Agar Sci., Essex, UK) coated copper slot grids, counterstained with uranyl acetate and Reynolds’s lead citrate, and examined on a JEOL JEM 1011 transmission electron microscope equipped with a Morada 11-megapixel camera (Olympus) using iTEM software.

### Statistical analysis

All experiments were carried out at least in triplicates. Values are presented as mean ± SE for all experimental data. Comparisons between groups were performed by using one-way or two-way ANOVAs followed by Dunn’s post hoc testing. Outliers were removed by Grubb’s test. The *p* value < 0.05 was considered to indicate statistical significance. If otherwise not indicated, statistical analyses and curve-fitting were performed in GraphPad Prism 8.3.0 software. Detailed results of all statistical analyses are presented in Supplementary Table 4.

## Results

### iPSC generation, NPC differentiation, molecular characterization

**Fig. 1:**
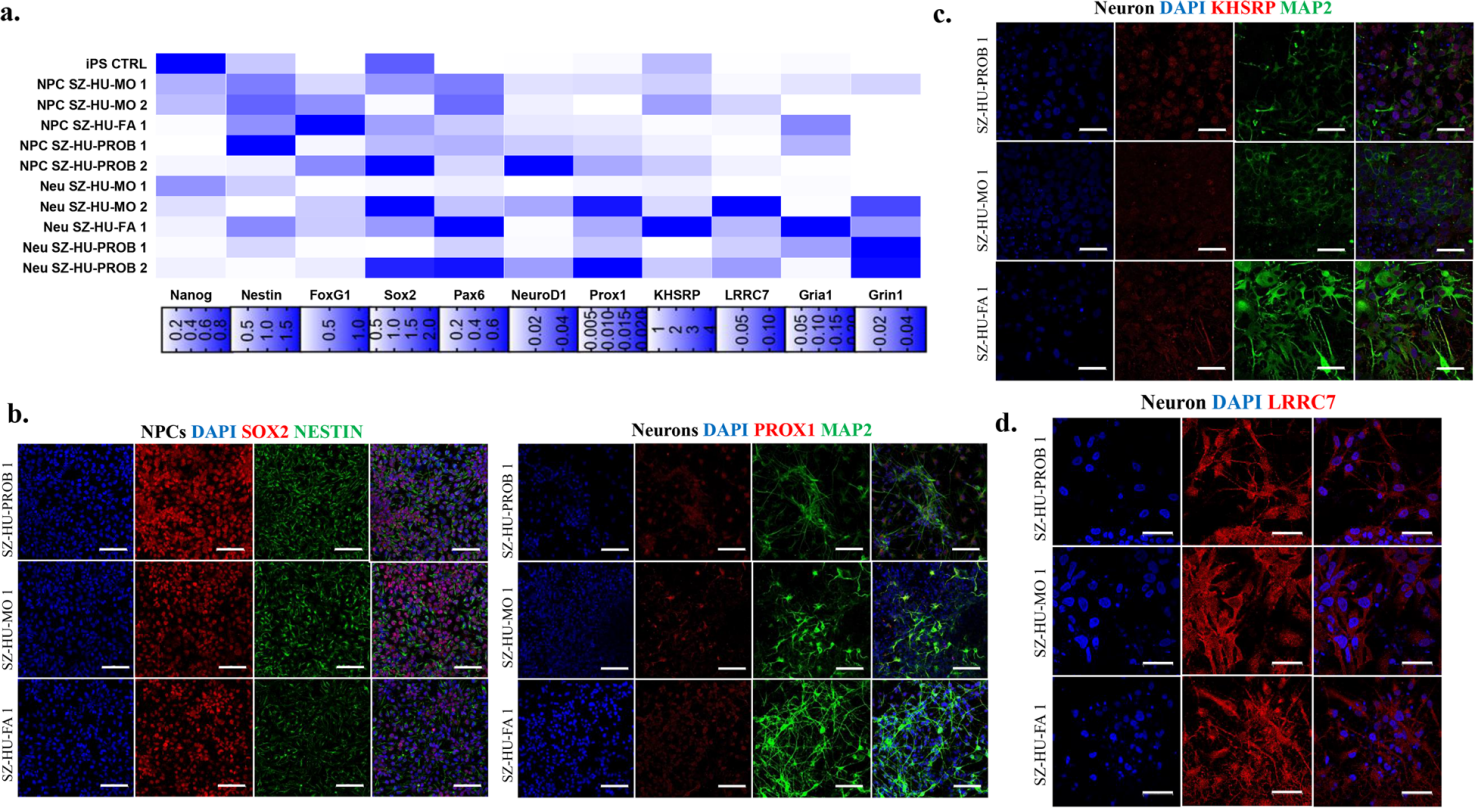
Establishment and molecular characterization of NPC lines and mature neuronal cultures. Investigation of target genes KHSRP and LRRC7. **a** Changes in gene expression patterns in NPCs and neurons derived from the case-parent trio. **b**, **c** NPCs and neurons derived from hiPSCs by the hippocampal neuronal differentiation protocol were investigated by immunofluorescence staining and visualized by confocal fluorescent microscopy. Immunocytochemical staining shows Nestin/Sox2 (**b**) and Map 2/Prox1 (**c**) positivity in these established neural cell types. Scale bars = 100 μm. **d**, **e** Immunofluorescence staining for KHSRP and LRRC7 in neurons. KHSRP (**d**) shows nuclear and cytoplasmic localization, while LRRC7 (**e**) localized postsynaptically in neurons. Scale bars = 50 μm

We successfully reprogrammed PBMCs into iPSCs for subsequent differentiation experiments. All iPSC lines demonstrated typical morphological properties, expressed pluripotency markers, and were devoid of karyotypic abnormalities. The previously described DNMs were also back-validated in iPSCs using Sanger sequencing. The iPSCs had low levels of spontaneous differentiation in pluripotency conditions, but were able to differentiate into all 3 germline layers, as revealed by spontaneous differentiation experiments (29 and Supplementary Fig. 1a-d.) The NPC lines generated form the case-parent trio demonstrated uniform molecular and morphological properties as captured by gene expression analysis (Fig. 1a) and immunofluorescence microscopy (Fig. 1b and Supplementary Fig. 2a), respectively. We observed no differences in the NPC lines’ efficiency to differentiate into neurons (Fig. 1a). The morphological properties and functional maturity of these neurons were tested by immunofluorescence staining (Fig. 1c and Supplementary Fig. 2b) and Ca^2+^-imaging (data not shown), respectively. We included the DNM harboring target genes KHSRP and LRRC7 in our molecular assays to test for potential differences at the RNA or protein expression levels, but found no significant differences (Fig. 1a, d, e).

### RNASeq analyses

**Fig. 2:**
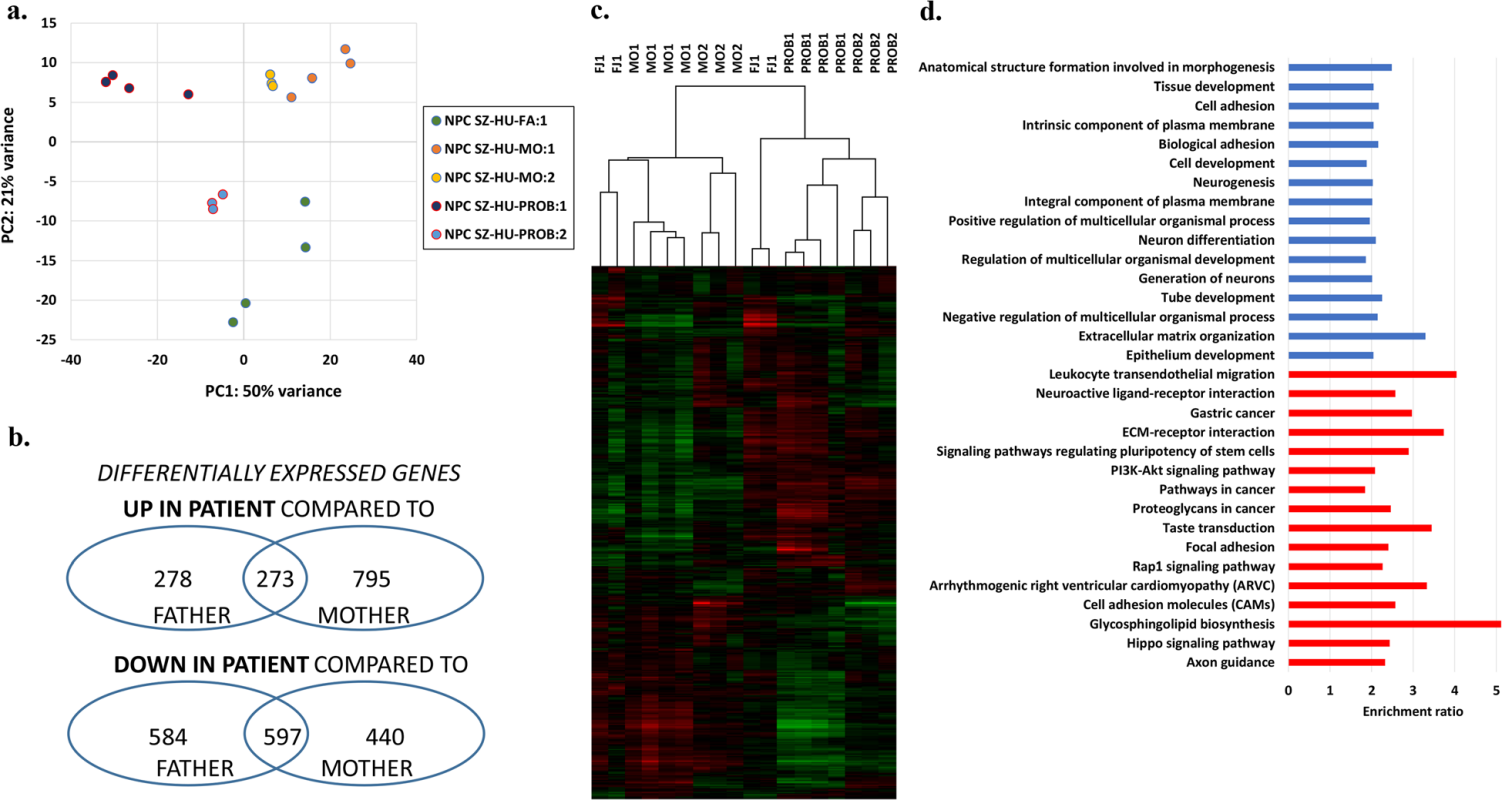
Assessment of transcriptomic differences in the trio NPC lines using RNASeq. **a**, **c** Principal component and cluster analysis of RPKM values demonstrates a clear separation between RNASeq samples from different members of the trio (*N* = 4 for clones 1 and *N* = 3 for clones 2). **b** Differential gene expression analysis identified significantly upregulated and downregulated genes. **d** Significantly enriched GO (blue) and PATHWAY (red) terms and fold enrichment values, listed in order of statistical significance (Bonferroni score < 0.05)

Next, to investigate whole-genome transcriptomic differences that might be associated with the DNMs carried by the proband, or his disease status, we carried out RNA sequencing analyses at the neuronal progenitor state. The RNASeq experiments and subsequent bioinformatics analyses demonstrated a clear separation of cell lines tested by principal component and cluster-analysis (Fig. 2a, c). We identified a set of differentially expressed (DE) genes that were down or upregulated in the SZ-HU-PROB NPC lines compared to both the paternal (SZ-HU-FA) and maternal (SZ-HU-MO) NPC lines (Fig. 2b and Supplementary Tables 1 and 2). These gene sets were taken forward for GO and PATHWAY analyses (Fig. 2d) that indicated the enrichment of DE genes in relevant biological pathways, including neuroactive ligand-receptor interaction, axon guidance, neurogenesis, neuronal differentiation Hippo and Wnt signaling, and Ca^2+^ signaling. Among the 100 top upregulated and downregulated genes were several transcription factor and neuron-specific genes.

To test for the possible enrichment of genes regulated by KHSRP in the DE gene set identified by the RNASeq experiments, we generated the list of KHSRP target genes using the oRNAment database. Using a hypergeometric test, we observed an enrichment of putative KHSRP target genes in the DE gene set identified by our RNASeq experiments (hypergeometric *p* = 0.03, Supplementary Table 3). Several of the differentially expressed KHSRP target genes, e.g., AUTS2, ERBB4, GRIN2A, and KHDRBS2 have been implicated in the etiology of SZ or ASD.

### Ca^2+^ imaging experiments

**Fig. 3:**
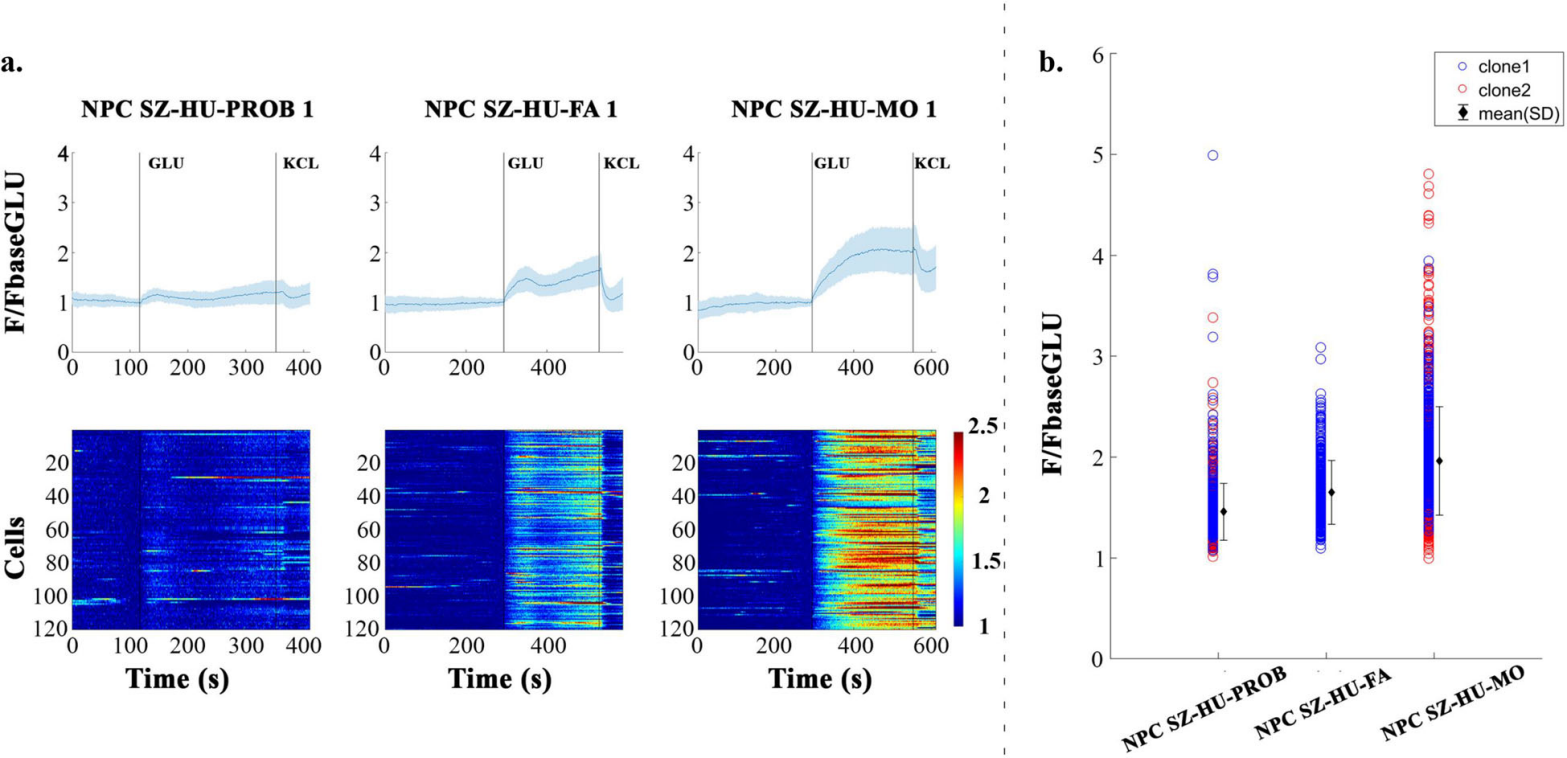
Investigation of calcium-activity in NPCs. **a** Normalized Ca^2+^ activity (F/F_baseGlu_) for one representative measurement of NPCs representing each subject. On the upper part of the subplot the *Y*-axis represents the mean activity (+/− standard deviation, shattered blue) and *X*-axis represents time. On the lower part, *Y*-axis represents the activity of all cells and *X*-axis represents time. Color indicates Ca^2+^ activity. **b** Each circle represents the normalized Ca^2+^ activity of one cell for each subject in each clone (*N* = 3 independent experiments, *n* = 200–250 cells/experiments). Black diamonds indicate the mean activity of a subject for both clones, while the error bars indicate one standard deviation. Red circles indicate NPCs derived from iPSC clone 1, blue circles NPCs derived from iPSC clone 2. For time series and heatmap images of clones 2, see Supplementary Fig. 3

As transcriptomic differences were indicative of synaptic, more specifically glutamatergic differences, and we wanted to test the functional activity of the progenitors, therefore, we used Ca^2+^ imaging to investigate the spontaneous activity and glutamate-evoked reactivity of NPCs. Similarly to our previous results, NPCs demonstrated low levels of spontaneous activity but reacted to stimulation with glutamate (Fig. 3a, b and Supplementary Fig. 3) [30]. The ANCOVA model yielded significant differences between subjects (*F* = 286.78, *p* < 0.001). Specifically, all subjects were significantly different from each other after adjusting for covariates (*F* = 837.62, *p* < 0.001). In addition, there was a significant subject-clone interaction (*F* = 30.57, *p* < 0.001). Post hoc analysis showed that the activity of cells in the second clone were larger compared to the corresponding first clone. Regarding the subjects, the proband-derived NPCs showed a significantly dampened reaction to glutamate compared to others. The NPCs of the mother showed the highest glutamate reactivity (Ca _NPC-SZ-HU-PROB_ < Ca _NPC-SZ-HU-FA_ < Ca _NPC-SZ-HU-MO_, Fig. 3a, b).

### Proliferation, migration, and neurite outgrowth tests in NPCs

**Fig. 4:**
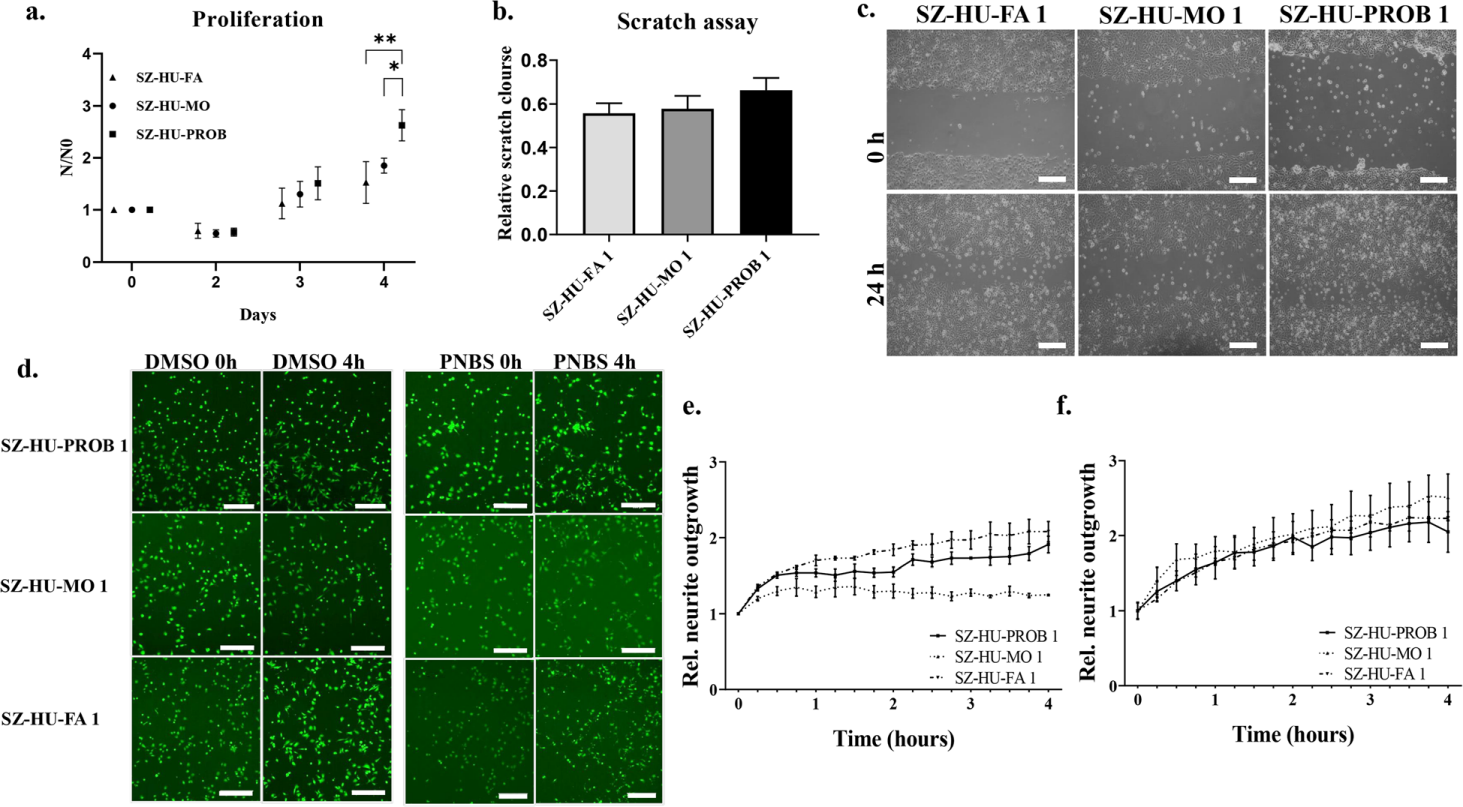
Proliferation, scratch, and neurite outgrowth in the trio NPC lines. **a** For the proliferation assay, 35,000 NPCs were plated onto poly-ornithine/laminin coated plates and were further cultured for 4 days. Cells were harvested daily and cell number was measured by ATTUNE NXT flow cytometer. Non-viable cells were excluded by PI staining. Cell counts relative to seeded cell numbers (N/N0) are plotted per day. Values represent the means ± SE (*N* = 3 independent experiments, *n* = 3 technical replicates/experiments). * *p* < 0.05, **p < 0.01. **b** Representative images of scratch assay in NPCs. Three parallel scratches were made per dish by a sterile P5 pipet. Three photos were taken along every scratch, at 24 h. Manual analysis was performed using ImageJ. The rate of closure was defined by measuring the width of the scratches. **c** Analysis of the scratch closure relative to day 0. The diagram shows mean values ± SE (*N* = 2 independent experiments, *n* = 9 measurements/experiments). Scale bars = 100 μm. **d**–**f** Representative images of neurite outgrowth assay. NPCs were treated with DMSO or para-nitroblebbistatin (PNBS) (10 μM). The changes of neurite outgrowth were monitored and analyzed using the ImageXpress Micro XLS Widefield High-Content Analysis System. Diagrams show the mean neurite outgrowth ± SE of DMSO and PNBS treated NPCs visualized by Calcein after 4 h, normalized to 0 h (*N* = 3 independent experiments). Scale bars = 200 μm

Based on earlier results from iPSC-based experiments in SZ and our transcriptomic findings, we also tested for potential differences in NPC proliferation and migration and furthermore neurite outgrowth speed of differentiating NPCs. The aggregate kinetics of proliferation, migration, and differentiation has been shown to determine the efficiency of neuronal differentiation in these progenitor populations. We found that NPCs derived from the proband, consistently with the upregulation of several Wnt-species in these cells (Fig. 2d and Supplementary Table 1), show significantly increased cell proliferation compared to the maternal (*p* = 0.0139) and paternal (*p* = 0.0039) NPCs at day 4 (Fig. 4a and Supplementary Fig. 4a; for statistical analysis, see Supplementary Table 4a). Comparisons of parallel clones from the same individuals did not yield significant differences in proliferation rates (Supplementary Fig. 4a, *p* = 0.7739 for maternal and *p* = 0.4527 for proband-derived NPCs, respectively). We found no significant differences in NPC migration, as measured by the scratch test (*p* = 0.5148), a functional assessment that measures both proliferation and migration kinetics (Fig. 4b, c and Supplementary Fig. 4b and for statistical analysis see Supplementary Table 4b). As a continuation of these experiments, we investigated neurite outgrowth in differentiating NPCs, either at baseline conditions (Fig. 4d, e) or by chemical stimulation of neurite outgrowth with para-nitroblebbistatin (PNBS) (Fig. 4d–f), an established inductor of neurite outgrowth. At baseline conditions, we observed accelerated neurite outgrowth in the proband-derived and paternal NPC lines compared to the maternal NPC line, and the differences became significant after 1 h of measurement. Treatment with PNBS abolished these differences (Fig. 4d–f, for statistical analysis see Supplementary Table 4c).

### Assessment of mitochondrial function and tolerance to oxidative stress

**Fig. 5:**
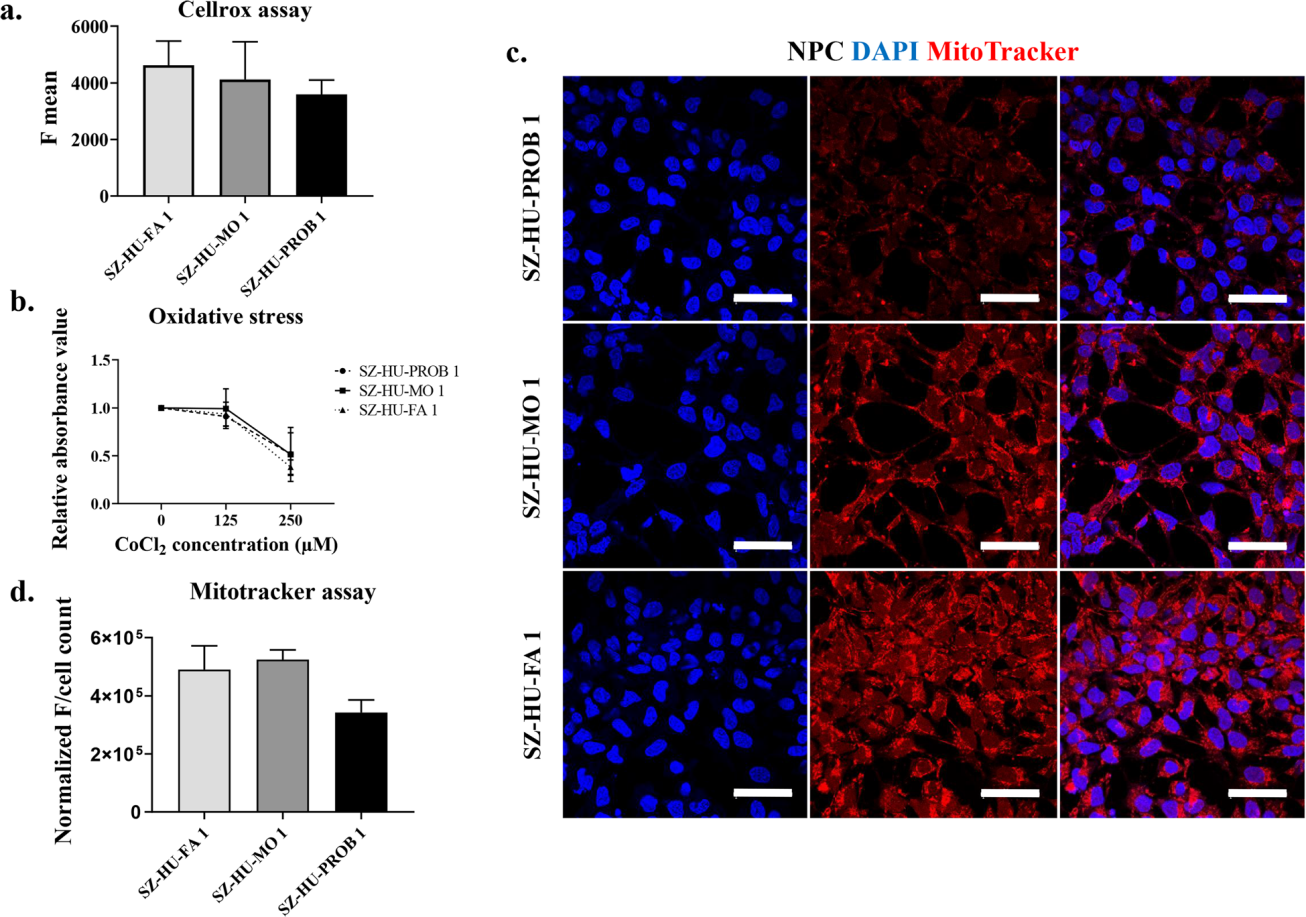
Assessment of mitochondrial function and tolerance to oxidative stress in the trio NPC lines. **a** Quantification of reactive oxygen species (ROS) by flow cytometry in NPCs using CellROX kit. Diagram shows the average of mean fluorescence signals ± SE of *N* = 3 independent experiments. **b** Effect of CoCl_2_ treatment on NPC cell survival. NPCs were treated with 125 and 250 μM CoCl_2_ for 24 h. After 48 h of reoxygenation, cell viability was measured by Presto Blue staining to determine the effect of oxidative stress. The diagram shows the mean values ± SE normalized to untreated cells (*N* = 5 independent experiments, *n* = 3 technical replicates/experiments). **c** Examination of mitochondrial function in NPCs. Fixed NPCs were stained using a fluorescent dye (MitoTracker™ Red CMXRos) and visualized with confocal microscopy. Scale bars = 50 μm. **d** Quantification of MitoTracker staining. Diagram shows the mean fluorescent intensities ± SE of NPC lines normalized to number of nuclei. Data were generated from confocal microscopy staining with ImageJ (*N* = 3 independent experiments, *n* = 4–6 pictures/experiments)

We used three independent methods to test for alterations in reactive oxygen species and tolerance to oxidative stress, phenotypes reported earlier in schizophrenia-derived cell lines. Surprisingly, there were lower levels of ROS in the proband-derived NPC line that were statistically not significant (*p* = 0.317, Fig. 5a, Supplementary Fig. 4c and for statistical analysis see Supplementary Table 4d), and no differences were found in the tolerance of cell lines to oxidative stress evoked by treatment with CoCl_2_ for 24 h and subsequent reoxygenation (*p* = 0.637, Fig. 5b). For statistical analysis, see Supplementary Table 4e.

Next, for quantification of mitochondria in NPCs, a mitochondrion-specific dye was used that allows measurement of functional mitochondria based on fluorescence intensity. This demonstrated a tendency (*p* = 0.064) for decreased quantity of functional mitochondria in NPC-SZ-HU-PROB in comparison to both the maternal cell line and the healthy control cell line (Fig. 5c, d and Supplementary Fig. 4d, for statistical analysis, see Supplementary Table 4f). Finally, we used transmission electron microscopy to investigate the morphology of mitochondria in the trio NPC lines. This high-resolution imaging method revealed abnormal, distensed, vacuolized, and broken mitochondria with irregular structure of cristae compared to the elongated, regularly membranized, often fusioned mitochondria found in NPC lines SZ-HU-MO and SZ-HU-FA (Supplementary Fig. 5, representative images).

## Discussion

In a series of experiments, we sought to investigate the biological significance of DNMs identified in a patient suffering from SZ, using iPSCs and in vitro neuronal differentiation protocols as a model system in a case-parent trio design. By the combination of several methodological approaches, we were able to identify molecular and functional phenotypes demonstrating differences between cell lines of the case-parent trio that could be connected to neurodevelopmental pathology and in part also to the investigated DNMs. The identified cell-autonomous phenotypes partially fall in line with previous iPSC-based disease modeling results of SZ; however, some of the results are more typical for ASD. To our best knowledge, this is the first study that has performed reprogramming in a whole trio to test for the putative molecular effects of DNMs in SZ. Similar studies can pave the way in the future for a personalized, precision medicine-based approach in the field of iPSC-based disease-modeling studies.

Consistent with previous findings, all iPSC lines were capable of neuronal differentiation, resulting in homogeneous SOX2 and NESTIN-expressing neuronal progenitors and, subsequently, by further differentiation in MAP 2 and PROX1-expressing, functional, dentate gyrus neurons. qPCR measurements demonstrated similarly efficient neuronal differentiation, as captured by the expression of neuronal marker genes NeuroD1, FOXG1, and PROX1 in the proband-derived NPCs and neuronal cultures. For NeuroD1 mRNA expression, we observed higher levels in the proband-derived NPCs compared to the paternal and maternal NPCs. Yu et al. reported a dampened expression of neuronal markers in SZ-derived differentiating neurons [31]; however, that study applied a case-control design. In our trio-based study, we found nearly equal expression of these markers between the investigated cell lines. The mature neuronal cultures that differentiated from the investigated NPC lines were functional, demonstrated by spontaneous activity in Ca-imaging experiments. However, we performed no direct comparisons at the neuronal level, since the primary focus of the study was the neuronal progenitor stage.

Whole-genome transcriptome analysis allows for the investigation of subtle, network-level differences between cell lines. The transcriptome-level alterations and the clear separation of samples from different individuals of the trio in the PCA and cluster-analysis serve as a proof-of-concept for the applied trio-based approach. The lists of upregulated and downregulated DE genes contain several candidate genes warranting further analysis. Among the upregulated DE genes, GSX1 is a transcription factor that plays an important role in the development of ventral telencephalon interneurons. NeuroD1 expression levels were also in the upregulated group, a finding that was also observed in the qPCR measurements. This transcription factor plays an important role in hippocampal neurogenesis [38]; its upregulation might be explained by precocious, accelerated development in the patient-derived NPCs. Wnt3A, Wnt6, Wnt7B, and Wnt10A both play an important role in brain and neuronal crest development. SCARA5, the most downregulated gene in the proband-derived NPCs, is associated with suicidal behavior in a recently published genome-wide association study [39]. Synapsin 3 (SYN3), another top downregulated DE gene, has been implicated in the regulation of hippocampal neurogenesis [40] and associated with SZ in genetic studies [41]. GO and PATHWAY analyses of upregulated and downregulated DE genes identified several enriched terms that are relevant for SZ (neuroactive ligand-receptor interaction, axon guidance, neurogenesis, neuronal differentiation, Hippo signaling, Wnt signaling) or for the involvement of KHSRP (transcriptional activator activity, RNA-binding). Of these, the most important finding is the involvement of Wnt signaling that has been established in several genetic and disease-modeling studies of SZ [14]. In the list of DE genes, we observed an enrichment of genes that are known or expected targets of KHSRP. We also observed misregulation of several miRNA-species; however, caution should be taken regarding these findings, as the resolution of the sequencing was not optimized for small RNAs. An important limitation of the transcriptomic analyses in this study was the fact that we did not use an external control, as in other experiments. Therefore, the identified differences are only based on comparisons between trio-members.

To test for functional differences between cell lines, we performed Ca^2+^ imaging experiments in NPC cultures. Previous work has shown that NPCs are amenable to measurements of intracellular Ca^2+^ signaling, which is reflective of the reactivity of neuronal progenitors to different ligands. Moreover, alterations have been shown both in SZ and ASD disease modeling-studies [31]. We tested the NPCs reactivity to glutamate, given the fact that dentate gyrus progenitors receive glutamatergic inputs. NPCs derived from the proband demonstrated decreased calcium reactivity to glutamate compared to the father- and mother-derived NPCs. Similarly to these results, Yu et al. found a lower level of spontaneous calcium-activity in SZ-derived cell lines; however, the spontaneous activity was measured in mature neurons and not NPCs. In vivo Ca^2+^ signaling plays an important role in neuronal differentiation and migration in the neuronal stem cell populations [42].

Various assessments of proliferation, migration, neurite outgrowth, oxidative stress, and mitochondrial function were carried out, and we could confirm differences between the trio NPC lines in proliferation and mitochondrial function. These phenotypes were informed by either transcriptomic differences identified in the RNASeq experiment or by previous findings of other groups. In particular, the increased rate of cell NPC proliferation observed in the proband-derived lines in our experiments and differences in migration found by other groups are consistent with the transcriptomic alterations in Wnt signaling and cell adhesion. Marchetto et al. demonstrated increased level of progenitor proliferation in idiopathic ASD-derived NPCs that was mediated by the upregulation of β-catenin signaling [17]. Similarly in a cohort of idiopathic ASD patients, Schäfer et al. showed the temporal dysregulation of specific gene networks that leads to growth acceleration in the patient-derived neuronal progenitors [43]. Both accelerated and decreased neurite outgrowth have also been implicated as important neurodevelopmental cell-autonomous phenotypes in ASD and SZ. In an iPSC-based model of Kleefstra-syndrome, a syndromic and genetically well described subtype of ASD, NPCs demonstrated increased proliferation, while neurite arborization was reduced in mature neurons [44]. It has also been shown that KHSRP regulates the mRNA-stability of GAP43, an important player in the process of axonal and dendritic growth [45]. Genetic manipulation of KHSRP manifested itself in altered axonal growth in mouse primary neuronal cultures. We were able to identify subtle difference in neurite outgrowth, with increased rates in the proband-derived and paternal progenitors. Both the increased NPC proliferation and accelerated neurite outgrowth are phenotypes that were previously associated with ASD; however, in the investigated SZ trio, they could also be identified. Grunwald et al. [46] used iPSC-based methodology to make head-to-head comparisons between SZ and ASD-derived neural cells and also found overlapping phenotypes; however, it was possible to discriminate SZ and AD-derived neurons by the combination of transcriptome analysis and Ca^2+^ imaging.

Finally, we found no alteration in tolerance to oxidative stress and levels of spontaneous reactive oxygen species. However, there was a tendency for lower number of functional mitochondria in the proband-derived NPCs, and electron microscopic images were suggestive of altered mitochondrial morphology in these cell lines. Increased oxidative stress and mitochondrial pathology have been previously implicated in several neurodevelopmental and neurodegenerative disorders, including SZ, ASD, amyotrophic lateral sclerosis, Parkinson’s disorder, and Alzheimer’s disorder. The main concept of this hypothesis is the dysregulation of redox systems in the developing brain that results in increased oxidative stress and mitochondrial pathology [47, 48]. Increased oxidative stress levels lead to the faulty migration and maturation in developing neurons and neuronal toxicity in the adult brain. Several lines of evidence suggest the involvement of mitochondrial dysfunction also in cellular models of SZ. Robiscsek et al. described that mitochondrial respiration and its sensitivity to dopamine-induced inhibition were impaired both in patient-derived keratinocytes and iPSCs. In differentiating dopaminergic neurons, they found altered mitochondrial network structure and connectivity [49]. In another study examining SZ-derived NPCs from 4 patients, both mitochondrial dysfunction (captured by the JC-1 red/green fluorescent dye measuring mitochondrial membrane potential) and oxidative stress, assessed by an OxyBlot procedure that quantifies the level of ROS-induced oxidized proteins, were elevated in the SZ-derived cell lines compared to healthy controls [50]. In the same cohort, immunohistochemical staining and TEM revealed altered mitochondrial pathology. In our NPCs, we found no differences in ROS; however, mitochondrial pathology could be demonstrated.

### Limitations

Limitations of this study have to be discussed. It has been mentioned that there is a bipolar patient in the investigated family, who was not included in the experiments. Therefore, it is highly probable that the SZ patient, besides the identified DNMs, which are attributable to the disorder, also carries a considerable level of genetic liability conveyed by common variants. Genome editing, e.g., CRISPR, which we did not use in our experiments, would be the most appropriate method to investigate the specific effects of the DNMs and connect the putative cellular phenotypes selectively to the DNMs. CNVs developing potentially during the reprogramming process were not screened in the iPSC lines.

The number of iPSC clones included in the study represents a significant limitation of the study. Two iPSC clones of the proband and the mother could be investigated, but only one for the father. Therefore, the standard of using multiple iPSC clones for each individual was not met fully in the study. However, the multiple clones that were examined yielded comparable results.

A number of studies connect LRRC7 function to neurodevelopmental pathology, including ASD, language impairment, and attention deficit hyperactivity disorder. Although LRRC7 is not an established ASD-gene, it is possible that the ASD-specific alterations unraveled in the presented experiments are associated with the DNM harbored in LRRC7, which we did not investigate at the same depth as the DNM in KHSPR.

## Conclusions

Using different methodological approaches, we could demonstrate several phenotypes in a SZ patient-derived NPC line. Many of these phenotypes, i.e., NPC proliferation, and synaptic activity have been associated previously with SZ and ASD as well. According to our results, it is conceivable that there is an overlap of phenotypes between SZ and ASD in the investigated family. These two disorders, although clinically distinct, show considerable degrees of overlap at the levels of symptomatology and genetics. Therefore, it is plausible that overlapping phenotypes are detectable also in disease-modeling studies.

## Supplementary information

**Additional file 1: Supplementary Fig. 1.** Reprograming, characterization and quality control of PBMC-derived control iPSC line CTRL (UCB2). a During reprogramming cells maintained a normal karyotype. b-c Immunofluorescence staining of pluripotency markers Oct4 and Nanog and flow-cytometry of SSEA4 cell surface marker was used to confirm the pluripotent state of the generated iPSC line. d The differentiation potential of iPSC line was demonstrated by the spontaneous differentiation assay, indicating positive staining with ectoderm (β-tubulin), endoderm (AFP) and mesoderm (SMA) germ layer markers. Scale bars = 100 μm.

**Additional file 2: Supplementary Fig. 2.** Differentiation and molecular characterization of NPC lines and neuronal cultures from additional iPSC clones. Sox2, Nestin staining for NPCs and Prox1, Map 2 staining for neurons from iPSC clone 2 of the proband and his mother, and the independent iPSC control CTRL (UCB2) line. Scale bars = 100 μm.

**Additional file 3: Supplementary Fig. 3.** Normalized Ca^2+^ activity (F/FbaseGlu) of NPCs derived from additional iPSC clones (clone2).

**Additional file 4: Supplementary Fig. 4.** Proliferation, scratch, CellRox and Mitotracker assays of all NPCs, demonstrating results for individual clones. **a** Diagrams show differences in proliferation in maternal (upper panel) and proband-derived (middle panel) NPCs, and all NPC clones (lower panel). Values represent the means±SE of cell counts relative to seeded cell numbers (N/N0) (*N* = 3 independent experiments, *n* = 3 technical replicates/experiments). No significant difference was found between two clones (clone 1 and 2) of maternal (*p* = 0.7739) and proband-derived (*p* = 0.4527) NPCs. **b** Scratch assay results for all NPC clones and the control NPC clone. The diagram shows scratch closure relative to day 0 (mean values±SE (*N* = 2 independent experiments, *n* = 9 measurements/experiments). No significant difference was found between additional clones (clone 2) of maternal and proband-derived NPCs (*p* = 0.1405). **c** CellRox assay results for all NPC clones showing no significant differences between the NPC clones (*p* = 0.3175) (N = 3 independent experiments). **d** Mitotracker assay results for all NPC clones showing no significant differences between the NPC clones (*p* = 0.0662). Confocal microscopy records were analyzed with ImageJ (N = 3 independent experiments, *n* = 4–6 pictures/experiments), data represents mean fluorescent intensities±SE normalized to number of nuclei.

**Additional file 5: Supplementary Fig. 5.** Mitochondrial morphology in mechanically dissociated NPC lines captured by transmission electron microscopy. Representative electron micrographs of ultrathin sections. Arrows indicate mitochondria. Scale bars = 0.5 μm.

**Additional file 6: Supplementary Table 1.** List of upregulated DE genes in NPC-SZ-HU-PROB compared to both NPC-SZ-HU-FA and NPC-SZ-HU-MO.

**Additional file 7: Supplementary Table 2.** List of downregulated DE genes in NPC-SZ-HU-PROB compared to both NPC-SZ-HU-FA and NPC-SZ-HU-MO.

**Additional file 8: Supplementary Table 3.** List of DE genes in NPC-SZ-HU-PROB compared to both NPC-SZ-HU-FA and NPC-SZ-HU-MO that are regulated by KHSRP.

**Additional file 9: Supplementary Table 4.** Detailed Statistical analyses.

## Data Availability

The RNASeq datasets generated during the current study are available at Gene Expression Omnibus (GEO) database (https://www.ncbi.nlm.nih.gov/geo/), Accession No: GSE145656. All other data are available from the corresponding author on reasonable request.

## Abbreviations

ASD: Autism spectrum disorder
CNV: Copy number variant
DE: Differentially expressed
DNM: De novo mutation
DPBS: Dulbecco’s modified PBS
EB: Embryoid body
GO: Gene Ontology
iPSC: Induced pluripotent stem cell
KHSRP: K-homology type splicing regulatory protein
KIR2DL1: Killer cell immunoglobulin-like receptor 2DL1
KOS: hKlf4, hOct3/4, hSox2
LRRC7: Leucine-rich repeat-containing 7 gene
MEF: Mouse embryonic fibroblast
NPC: Neuronal progenitor cell
PBMC: Peripheral mononuclear cell
PNBS: para-Nitroblebbistatin
ROS: Reactive oxygen species
SNP: Single-nucleotide polymorphism
SNV: Single-nucleotide variant
SZ: Schizophrenia
