# Supplementary figures and images for: Investigation of de novo mutations in a schizophrenia case-parent trio by induced pluripotent stem cell-based in vitro disease modeling: convergence of schizophrenia- and autism-related cellular phenotypes
Source: Stem Cell Res Ther. 2020 Nov 27;11:504. doi: 10.1186/s13287-020-01980-5 (PMC7694414; doi:10.1186/s13287-020-01980-5)

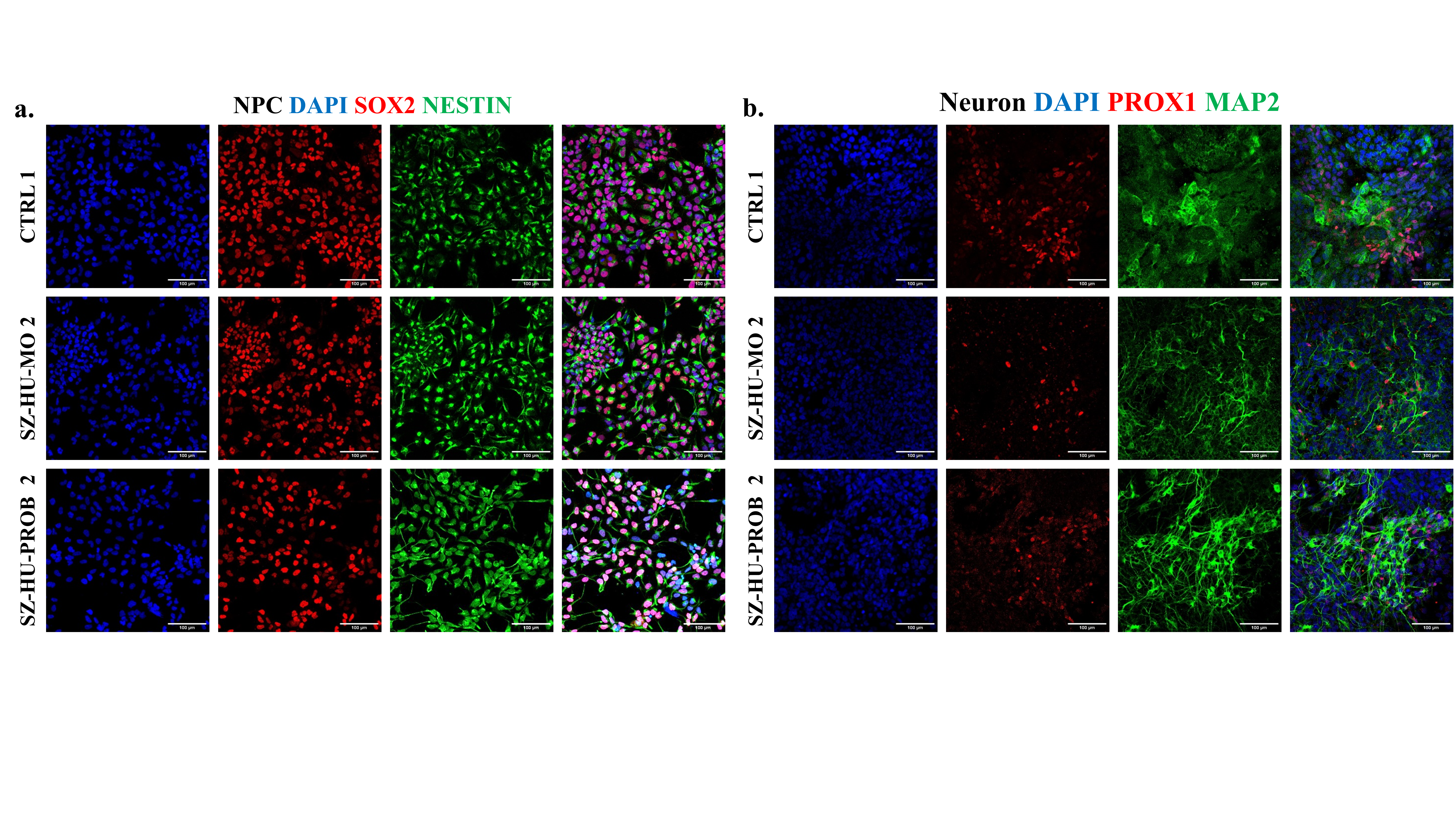

Supplement: Supplementary file 2 — Additional file 2: Supplementary Fig. 2. Differentiation and molecular characterization of NPC lines and neuronal cultures from additional iPSC clones. Sox2, Nestin staining for NPCs and Prox1, Map 2 staining for neurons from iPSC clone 2 of the proband and his mother, and the independent iPSC control CTRL (UCB2) line. Scale bars = 100 μm. [file 13287_2020_1980_MOESM2_ESM.tif]
