# Supplementary material for: Investigation of de novo mutations in a schizophrenia case-parent trio by induced pluripotent stem cell-based in vitro disease modeling: convergence of schizophrenia- and autism-related cellular phenotypes
Source: Stem Cell Res Ther. 2020 Nov 27;11:504. doi: 10.1186/s13287-020-01980-5 (PMC7694414; doi:10.1186/s13287-020-01980-5)
Supplement: Supplementary file 6 — Additional file 6: Supplementary Table 1. List of upregulated DE genes in NPC-SZ-HU-PROB compared to both NPC-SZ-HU-FA and NPC-SZ-HU-MO. [file 13287_2020_1980_MOESM6_ESM.docx]

**Supplementary Table 1. List of upregulated DE genes in NPC-SZ-HU-PROB compared to both NPC-SZ-HU-FA and NPC-SZ-HU-MO.**

**N/A = non-applicable**

| ORDER | ENTREZ ID | SYMBOL | NAME | NPC-SZ-HU-PROB/NPC-SZ-HU-FA LOGFC | NPC-SZ-HU-PROB/NPC-SZ-HU-MO LOGFC | AVG LOGFC | STD |
| --- | --- | --- | --- | --- | --- | --- | --- |
| 1 | 81285 | OR51E2 | olfactory receptor family 51 subfamily E member 2 | 8.64 | 6.58 | 7.61 | 1.45 |
| 2 | 92293 | TMEM132C | transmembrane protein 132C | 8.62 | 6.05 | 7.33 | 1.81 |
| 3 | 3887 | KRT81 | keratin 81 | 6.81 | 4.82 | 5.82 | 1.40 |
| 4 | 1293 | COL6A3 | collagen type VI alpha 3 chain | 6.33 | 5.22 | 5.78 | 0.79 |
| 5 | 219409 | GSX1 | GS homeobox 1 | 6.19 | 5.34 | 5.76 | 0.60 |
| 6 | 572558 | PGM5-AS1 | PGM5 antisense RNA 1 | 5.79 | 5.26 | 5.52 | 0.37 |
| 7 | 8929 | PHOX2B | paired like homeobox 2b | 6.53 | 4.14 | 5.34 | 1.69 |
| 8 | 256297 | PTF1A | pancreas specific transcription factor. 1a | 5.68 | 4.90 | 5.29 | 0.55 |
| 9 | 4632 | MYL1 | myosin light chain 1 | 5.97 | 4.41 | 5.19 | 1.10 |
| 10 | 9607 | CARTPT | CART prepropeptide | 5.66 | 4.66 | 5.16 | 0.71 |
| 11 | 7475 | WNT6 | Wnt family member 6 | 5.94 | 3.88 | 4.91 | 1.45 |
| 12 | 9033 | PKD2L1 | polycystin 2 like 1. transient receptor potential cation channel | 5.94 | 3.87 | 4.91 | 1.46 |
| 13 | 4256 | MGP | matrix Gla protein | 5.81 | 3.45 | 4.63 | 1.67 |
| 14 | 2681 | GGTA1P | glycoprotein. alpha-galactosyltransferase 1 pseudogene | 6.73 | 2.51 | 4.62 | 2.98 |
| 15 | 5239 | PGM5 | phosphoglucomutase 5 | 4.79 | 4.41 | 4.60 | 0.27 |
| 16 | 151278 | CCDC140 | coiled-coil domain containing 140 | 5.16 | 4.02 | 4.59 | 0.80 |
| 17 | 131377 | KLHL40 | kelch like family member 40 | 5.83 | 3.30 | 4.56 | 1.79 |
| 18 | 1303 | COL12A1 | collagen type XII alpha 1 chain | 5.60 | 3.47 | 4.54 | 1.51 |
| 19 | 80731 | THSD7B | thrombospondin type 1 domain containing 7B | 5.39 | 3.55 | 4.47 | 1.30 |
| 20 | 399806 | LBX1-AS1 | LBX1 antisense RNA 1 (head to head) | 5.61 | 3.17 | 4.39 | 1.73 |
| 21 | 5081 | PAX7 | paired box 7 | 3.13 | 5.51 | 4.32 | 1.68 |
| 22 | 5396 | PRRX1 | paired related homeobox 1 | 5.69 | 2.87 | 4.28 | 2.00 |
| 23 | 50509 | COL5A3 | collagen type V alpha 3 chain | 4.28 | 4.24 | 4.26 | 0.03 |
| 24 | 10660 | LBX1 | ladybird homeobox 1 | 5.18 | 3.27 | 4.22 | 1.35 |
| 25 | 1278 | COL1A2 | collagen type I alpha 2 chain | 5.27 | 3.16 | 4.21 | 1.49 |
| 26 | 4060 | LUM | lumican | 5.09 | 3.33 | 4.21 | 1.25 |
| 27 | 5077 | PAX3 | paired box 3 | 4.14 | 4.19 | 4.16 | 0.04 |
| 28 | 3195 | TLX1 | T-cell leukemia homeobox 1 | 5.66 | 2.62 | 4.14 | 2.15 |
| 29 | 59336 | PRDM13 | PR/SET domain 13 | 5.17 | 2.96 | 4.07 | 1.56 |
| 30 | 7020 | TFAP2A | transcription factor AP-2 alpha | 4.21 | 3.89 | 4.05 | 0.23 |
| 31 | 3196 | TLX2 | T-cell leukemia homeobox 2 | 5.02 | 2.88 | 3.95 | 1.51 |
| 32 | 79148 | MMP28 | matrix metallopeptidase 28 | 4.53 | 3.30 | 3.91 | 0.87 |
| 33 | 3814 | KISS1 | KiSS-1 metastasis-suppressor | 4.18 | 3.62 | 3.90 | 0.40 |
| 34 | 8840 | CCN4 | WNT1 inducible signaling pathway protein 1 | 4.53 | 3.22 | 3.88 | 0.92 |
| 35 | 4760 | NEUROD1 | neuronal differentiation 1 | 2.74 | 4.97 | 3.85 | 1.58 |
| 36 | 5458 | POU4F2 | POU class 4 homeobox 2 | 4.72 | 2.97 | 3.85 | 1.23 |
| 37 | 7021 | TFAP2B | transcription factor AP-2 beta | 4.11 | 3.48 | 3.80 | 0.45 |
| 38 | 146395 | GSG1L | GSG1 like | 3.53 | 3.92 | 3.72 | 0.28 |
| 39 | 2020 | EN2 | engrailed homeobox 2 | 5.14 | 2.13 | 3.64 | 2.13 |
| 40 | 1307 | COL16A1 | collagen type XVI alpha 1 chain | 4.14 | 3.13 | 3.63 | 0.71 |
| 41 | 54715 | RBFOX1 | RNA binding protein. fox-1 homolog 1 | 5.24 | 2.03 | 3.63 | 2.27 |
| 42 | 2191 | FAP | fibroblast activation protein alpha | 3.59 | 3.49 | 3.54 | 0.07 |
| 43 | 3201 | HOXA4 | homeobox A4 | 4.51 | 2.53 | 3.52 | 1.40 |
| 44 | 56978 | PRDM8 | PR/SET domain 8 | 4.14 | 2.70 | 3.42 | 1.02 |
| 45 | 2139 | EYA2 | EYA transcriptional coactivator and phosphatase 2 | 2.65 | 4.12 | 3.38 | 1.04 |
| 46 | 58158 | NEUROD4 | neuronal differentiation 4 | 2.37 | 4.39 | 3.38 | 1.42 |
| 47 | 4010 | LMX1B | LIM homeobox transcription factor 1 beta | 3.41 | 3.30 | 3.35 | 0.07 |
| 48 | 4969 | OGN | osteoglycin | 4.12 | 2.56 | 3.34 | 1.11 |
| 49 | 285888 | CNPY1 | canopy FGF signaling regulator 1 | 3.82 | 2.83 | 3.33 | 0.70 |
| 50 | 4880 | NPPC | natriuretic peptide C | 4.09 | 2.48 | 3.28 | 1.14 |
| 51 | 124857 | WFIKKN2 | WAP. follistatin/kazal. immunoglobulin. kunitz and netrin domain containing 2 | 2.69 | 3.82 | 3.26 | 0.80 |
| 52 | 100505639 | N/A | N/A | 3.30 | 3.18 | 3.24 | 0.09 |
| 53 | 10320 | IKZF1 | IKAROS family zinc finger 1 | 4.15 | 2.32 | 3.24 | 1.29 |
| 54 | 1013 | CDH15 | cadherin 15 | 3.98 | 2.45 | 3.22 | 1.08 |
| 55 | 7291 | TWIST1 | twist family bHLH transcription factor 1 | 4.36 | 2.00 | 3.18 | 1.67 |
| 56 | 57615 | ZNF492 | zinc finger protein 492 | 3.29 | 3.00 | 3.14 | 0.20 |
| 57 | 10516 | FBLN5 | fibulin 5 | 3.52 | 2.72 | 3.12 | 0.56 |
| 58 | 347853 | TBX10 | T-box 10 | 3.85 | 2.34 | 3.09 | 1.06 |
| 59 | 22844 | FRMPD1 | FERM and PDZ domain containing 1 | 2.92 | 3.24 | 3.08 | 0.23 |
| 60 | 28526 | TRDC | T cell receptor delta constant | 2.64 | 3.52 | 3.08 | 0.63 |
| 61 | 6900 | CNTN2 | contactin 2 | 2.19 | 3.92 | 3.06 | 1.22 |
| 62 | 728606 | PCAT18 | prostate cancer associated transcript 18 (non-protein coding) | 3.24 | 2.86 | 3.05 | 0.27 |
| 63 | 1421 | CRYGD | crystallin gamma D | 3.11 | 2.96 | 3.03 | 0.11 |
| 64 | 22801 | ITGA11 | integrin subunit alpha 11 | 4.12 | 1.91 | 3.01 | 1.56 |
| 65 | 121256 | TMEM132D | transmembrane protein 132D | 3.38 | 2.62 | 3.00 | 0.54 |
| 66 | 23316 | CUX2 | cut like homeobox 2 | 2.94 | 3.02 | 2.98 | 0.05 |
| 67 | 202559 | KHDRBS2 | KH RNA binding domain containing. signal transduction associated 2 | 2.03 | 3.87 | 2.95 | 1.31 |
| 68 | 135152 | B3GAT2 | beta-1.3-glucuronyltransferase 2 | 3.24 | 2.65 | 2.95 | 0.42 |
| 69 | 9547 | CXCL14 | C-X-C motif chemokine ligand 14 | 2.28 | 3.58 | 2.93 | 0.92 |
| 70 | 3204 | HOXA7 | homeobox A7 | 3.93 | 1.91 | 2.92 | 1.43 |
| 71 | 26082 | LINC02249 | uncharacterized LOC26082 | 3.79 | 2.05 | 2.92 | 1.23 |
| 72 | 100192378 | ZFHX4-AS1 | ZFHX4 antisense RNA 1 | 2.94 | 2.87 | 2.91 | 0.05 |
| 73 | 3763 | KCNJ6 | potassium voltage-gated channel subfamily J member 6 | 2.56 | 3.16 | 2.86 | 0.43 |
| 74 | 594855 | CPLX3 | complexin 3 | 3.36 | 2.32 | 2.84 | 0.73 |
| 75 | 79190 | IRX6 | iroquois homeobox 6 | 3.01 | 2.61 | 2.81 | 0.28 |
| 76 | 133584 | EGFLAM | EGF like. fibronectin type III and laminin G domains | 2.73 | 2.84 | 2.79 | 0.08 |
| 77 | 4807 | NHLH1 | nescient helix-loop-helix 1 | 2.35 | 3.16 | 2.76 | 0.57 |
| 78 | 85407 | NKD1 | naked cuticle homolog 1 | 2.78 | 2.71 | 2.75 | 0.05 |
| 79 | 400576 | N/A | uncharacterized LOC400576 | 2.50 | 2.95 | 2.72 | 0.32 |
| 80 | 145845 | LOC145845 | uncharacterized LOC145845 | 3.52 | 1.85 | 2.68 | 1.18 |
| 81 | 10351 | ABCA8 | ATP binding cassette subfamily A member 8 | 2.87 | 2.48 | 2.67 | 0.28 |
| 82 | 3776 | KCNK2 | potassium two pore domain channel subfamily K member 2 | 3.36 | 1.96 | 2.66 | 0.99 |
| 83 | 2741 | GLRA1 | glycine receptor alpha 1 | 3.35 | 1.90 | 2.62 | 1.02 |
| 84 | 375298 | CERKL | ceramide kinase like | 2.10 | 3.15 | 2.62 | 0.74 |
| 85 | 2066 | ERBB4 | erb-b2 receptor tyrosine kinase 4 | 2.71 | 2.54 | 2.62 | 0.12 |
| 86 | 6345 | SRL | sarcalumenin | 3.41 | 1.81 | 2.61 | 1.13 |
| 87 | 5153 | PDE1B | phosphodiesterase 1B | 2.33 | 2.85 | 2.59 | 0.36 |
| 88 | 6750 | SST | somatostatin | 2.56 | 2.59 | 2.58 | 0.02 |
| 89 | 7122 | CLDN5 | claudin 5 | 3.36 | 1.79 | 2.58 | 1.12 |
| 90 | 63973 | NEUROG2 | neurogenin 2 | 2.43 | 2.72 | 2.57 | 0.21 |
| 91 | 440730 | TRIM67 | tripartite motif containing 67 | 1.89 | 3.26 | 2.57 | 0.97 |
| 92 | 147495 | APCDD1 | APC down-regulated 1 | 2.94 | 2.13 | 2.54 | 0.58 |
| 93 | 5457 | POU4F1 | POU class 4 homeobox 1 | 2.80 | 2.27 | 2.53 | 0.37 |
| 94 | 4487 | MSX1 | msh homeobox 1 | 1.98 | 3.07 | 2.53 | 0.77 |
| 95 | 64388 | GREM2 | gremlin 2. DAN family BMP antagonist | 2.67 | 2.35 | 2.51 | 0.23 |
| 96 | 7477 | WNT7B | Wnt family member 7B | 2.31 | 2.67 | 2.49 | 0.26 |
| 97 | 1012 | CDH13 | cadherin 13 | 2.59 | 2.38 | 2.49 | 0.14 |
| 98 | 5178 | PEG3 | paternally expressed 3 | 2.21 | 2.73 | 2.47 | 0.37 |
| 99 | 7043 | TGFB3 | transforming growth factor beta 3 | 3.08 | 1.81 | 2.45 | 0.90 |
| 100 | 6752 | SSTR2 | somatostatin receptor 2 | 2.44 | 2.42 | 2.43 | 0.01 |
| 101 | 89780 | WNT3A | Wnt family member 3A | 2.40 | 2.46 | 2.43 | 0.04 |
| 102 | 8706 | B3GALNT1 | beta-1.3-N-acetylgalactosaminyltransferase 1 (globoside blood group) | 2.57 | 2.23 | 2.40 | 0.24 |
| 103 | 869 | CBLN1 | cerebellin 1 precursor | 2.78 | 2.01 | 2.40 | 0.54 |
| 104 | 253738 | EBF3 | early B-cell factor 3 | 2.87 | 1.91 | 2.39 | 0.67 |
| 105 | 90050 | FAM181A | family with sequence similarity 181 member A | 1.41 | 3.25 | 2.33 | 1.30 |
| 106 | 3269 | HRH1 | histamine receptor H1 | 2.55 | 2.08 | 2.31 | 0.33 |
| 107 | 401286 | LINC02538 | uncharacterized LOC401286 | 2.23 | 2.40 | 2.31 | 0.12 |
| 108 | 285533 | RNF175 | ring finger protein 175 | 2.85 | 1.77 | 2.31 | 0.76 |
| 109 | 124460 | SNX20 | sorting nexin 20 | 1.91 | 2.69 | 2.30 | 0.55 |
| 110 | 57604 | TRMT9B | KIAA1456 | 2.14 | 2.46 | 2.30 | 0.23 |
| 111 | 81615 | TMEM163 | transmembrane protein 163 | 2.33 | 2.25 | 2.29 | 0.05 |
| 112 | 1545 | CYP1B1 | cytochrome P450 family 1 subfamily B member 1 | 2.85 | 1.74 | 2.29 | 0.79 |
| 113 | 57125 | PLXDC1 | plexin domain containing 1 | 2.22 | 2.34 | 2.28 | 0.09 |
| 114 | 92092 | ZC3HAV1L | zinc finger CCCH-type containing. antiviral 1 like | 2.67 | 1.89 | 2.28 | 0.55 |
| 115 | 390660 | ADAMTS7P1 | ADAMTS7 pseudogene 1 | 1.02 | 3.49 | 2.26 | 1.75 |
| 116 | 151647 | TAFA4 | family with sequence similarity 19 member A4. C-C motif chemokine like | 2.73 | 1.78 | 2.25 | 0.67 |
| 117 | 11155 | LDB3 | LIM domain binding 3 | 2.08 | 2.39 | 2.24 | 0.21 |
| 118 | 10265 | IRX5 | iroquois homeobox 5 | 1.99 | 2.48 | 2.24 | 0.35 |
| 119 | 145270 | PRIMA1 | proline rich membrane anchor 1 | 2.01 | 2.44 | 2.23 | 0.31 |
| 120 | 64641 | EBF2 | early B-cell factor 2 | 2.86 | 1.55 | 2.20 | 0.93 |
| 121 | 5013 | OTX1 | orthodenticle homeobox 1 | 1.82 | 2.59 | 2.20 | 0.54 |
| 122 | 10319 | LAMC3 | laminin subunit gamma 3 | 2.90 | 1.46 | 2.18 | 1.02 |
| 123 | 8641 | PCDHGB4 | protocadherin gamma subfamily B. 4 | 2.07 | 2.28 | 2.18 | 0.15 |
| 124 | 730668 | LOC730668 | dynein heavy chain -like pseudogene | 2.72 | 1.63 | 2.17 | 0.77 |
| 125 | 162494 | RHBDL3 | rhomboid like 3 | 2.21 | 2.12 | 2.17 | 0.06 |
| 126 | 10882 | C1QL1 | complement C1q like 1 | 2.10 | 2.23 | 2.17 | 0.09 |
| 127 | 3748 | KCNC3 | potassium voltage-gated channel subfamily C member 3 | 2.48 | 1.84 | 2.16 | 0.46 |
| 128 | 25758 | KIAA1549L | KIAA1549 like | 1.77 | 2.49 | 2.13 | 0.51 |
| 129 | 83643 | CCDC3 | coiled-coil domain containing 3 | 3.07 | 1.18 | 2.13 | 1.33 |
| 130 | 64221 | ROBO3 | roundabout guidance receptor 3 | 2.32 | 1.93 | 2.12 | 0.28 |
| 131 | 5502 | PPP1R1A | protein phosphatase 1 regulatory inhibitor subunit 1A | 1.39 | 2.84 | 2.12 | 1.03 |
| 132 | 7484 | WNT9B | Wnt family member 9B | 2.36 | 1.81 | 2.09 | 0.39 |
| 133 | 84466 | MEGF10 | multiple EGF like domains 10 | 2.60 | 1.57 | 2.09 | 0.73 |
| 134 | 7092 | TLL1 | tolloid like 1 | 2.33 | 1.83 | 2.08 | 0.35 |
| 135 | 11211 | FZD10 | frizzled class receptor 10 | 2.84 | 1.29 | 2.07 | 1.10 |
| 136 | 93145 | OLFM2 | olfactomedin 2 | 2.03 | 2.09 | 2.06 | 0.04 |
| 137 | 5024 | P2RX3 | purinergic receptor P2X 3 | 2.09 | 1.97 | 2.03 | 0.09 |
| 138 | 100506841 | N/A | N/A | 1.64 | 2.42 | 2.03 | 0.55 |
| 139 | 845 | CASQ2 | calsequestrin 2 | 1.73 | 2.32 | 2.02 | 0.41 |
| 140 | 81706 | PPP1R14C | protein phosphatase 1 regulatory inhibitor subunit 14C | 1.85 | 2.18 | 2.02 | 0.23 |
| 141 | 89832 | CHRFAM7A | CHRNA7 (exons 5-10) and FAM7A (exons A-E) fusion | 2.24 | 1.79 | 2.02 | 0.32 |
| 142 | 1297 | COL9A1 | collagen type IX alpha 1 chain | 1.13 | 2.90 | 2.01 | 1.25 |
| 143 | 2911 | GRM1 | glutamate metabotropic receptor 1 | 2.28 | 1.74 | 2.01 | 0.38 |
| 144 | 79625 | NDNF | neuron derived neurotrophic factor | 2.04 | 1.97 | 2.00 | 0.05 |
| 145 | 7852 | CXCR4 | C-X-C motif chemokine receptor 4 | 2.03 | 1.97 | 2.00 | 0.04 |
| 146 | 4868 | NPHS1 | NPHS1. nephrin | 2.19 | 1.73 | 1.96 | 0.33 |
| 147 | 3166 | HMX1 | H6 family homeobox 1 | 2.38 | 1.51 | 1.94 | 0.62 |
| 148 | 55502 | HES6 | hes family bHLH transcription factor 6 | 1.68 | 2.18 | 1.93 | 0.35 |
| 149 | 1146 | CHRNG | cholinergic receptor nicotinic gamma subunit | 1.88 | 1.97 | 1.93 | 0.06 |
| 150 | 131096 | KCNH8 | potassium voltage-gated channel subfamily H member 8 | 1.51 | 2.32 | 1.91 | 0.57 |
| 151 | 161635 | CSNK1A1P1 | casein kinase 1 alpha 1 pseudogene 1 | 2.50 | 1.32 | 1.91 | 0.83 |
| 152 | 127833 | SYT2 | synaptotagmin 2 | 2.34 | 1.46 | 1.90 | 0.62 |
| 153 | 343052 | LOC343052 | immunoglobulin superfamily DCC subclass member 3 pseudogene | 1.47 | 2.32 | 1.90 | 0.60 |
| 154 | 83541 | FAM110A | family with sequence similarity 110 member A | 1.70 | 2.07 | 1.89 | 0.27 |
| 155 | 440934 | CT75 | uncharacterized LOC440934 | 2.57 | 1.20 | 1.88 | 0.97 |
| 156 | 7424 | VEGFC | vascular endothelial growth factor C | 2.08 | 1.66 | 1.87 | 0.30 |
| 157 | 59335 | PRDM12 | PR/SET domain 12 | 2.68 | 1.04 | 1.86 | 1.16 |
| 158 | 6489 | ST8SIA1 | ST8 alpha-N-acetyl-neuraminide alpha-2.8-sialyltransferase 1 | 1.59 | 2.11 | 1.85 | 0.37 |
| 159 | 117 | ADCYAP1R1 | ADCYAP receptor type I | 1.17 | 2.51 | 1.84 | 0.95 |
| 160 | 100505556 | N/A | N/A | 2.37 | 1.30 | 1.84 | 0.76 |
| 161 | 5362 | PLXNA2 | plexin A2 | 2.03 | 1.64 | 1.83 | 0.27 |
| 162 | 79825 | EFCC1 | EF-hand and coiled-coil domain containing 1 | 1.39 | 2.27 | 1.83 | 0.63 |
| 163 | 56967 | C14orf132 | chromosome 14 open reading frame 132 | 1.83 | 1.81 | 1.82 | 0.02 |
| 164 | 5100 | PCDH8 | protocadherin 8 | 2.12 | 1.51 | 1.82 | 0.43 |
| 165 | 8549 | LGR5 | leucine rich repeat containing G protein-coupled receptor 5 | 1.59 | 2.04 | 1.81 | 0.32 |
| 166 | 1136 | CHRNA3 | cholinergic receptor nicotinic alpha 3 subunit | 1.99 | 1.62 | 1.81 | 0.26 |
| 167 | 84457 | PHYHIPL | phytanoyl-CoA 2-hydroxylase interacting protein like | 2.38 | 1.23 | 1.80 | 0.82 |
| 168 | 114795 | TMEM132B | transmembrane protein 132B | 1.98 | 1.62 | 1.80 | 0.25 |
| 169 | 5121 | PCP4 | Purkinje cell protein 4 | 2.25 | 1.32 | 1.79 | 0.66 |
| 170 | 57452 | GALNT16 | polypeptide N-acetylgalactosaminyltransferase 16 | 1.07 | 2.49 | 1.78 | 1.00 |
| 171 | 642236 | FRG1JP | FSHD region gene 1 family member J. pseudogene | 2.43 | 1.13 | 1.78 | 0.92 |
| 172 | 440119 | FZD10-AS1 | FZD10 antisense RNA 1 (head to head) | 2.14 | 1.41 | 1.77 | 0.52 |
| 173 | 157869 | SBSPON | somatomedin B and thrombospondin type 1 domain containing | 2.08 | 1.45 | 1.77 | 0.44 |
| 174 | 374393 | FAM111B | family with sequence similarity 111 member B | 1.30 | 2.21 | 1.75 | 0.64 |
| 175 | 1143 | CHRNB4 | cholinergic receptor nicotinic beta 4 subunit | 1.71 | 1.79 | 1.75 | 0.06 |
| 176 | 283212 | KLHL35 | kelch like family member 35 | 1.67 | 1.82 | 1.75 | 0.11 |
| 177 | 407003 | MIR219A2 | microRNA 219a-2 | 2.16 | 1.31 | 1.73 | 0.60 |
| 178 | 284600 | LOC284600 | uncharacterized LOC284600 | 1.73 | 1.72 | 1.73 | 0.00 |
| 179 | 100506065 | N/A | N/A | 2.21 | 1.23 | 1.72 | 0.69 |
| 180 | 100132215 | LOC100132215 | uncharacterized LOC100132215 | 1.70 | 1.74 | 1.72 | 0.02 |
| 181 | 7473 | WNT3 | Wnt family member 3 | 1.92 | 1.51 | 1.72 | 0.29 |
| 182 | 2912 | GRM2 | glutamate metabotropic receptor 2 | 1.24 | 2.19 | 1.71 | 0.67 |
| 183 | 83445 | GSG1 | germ cell associated 1 | 2.03 | 1.39 | 1.71 | 0.45 |
| 184 | 1813 | DRD2 | dopamine receptor D2 | 1.39 | 2.00 | 1.70 | 0.43 |
| 185 | 56130 | PCDHB6 | protocadherin beta 6 | 2.21 | 1.17 | 1.69 | 0.73 |
| 186 | 389125 | MUSTN1 | musculoskeletal. embryonic nuclear protein 1 | 1.58 | 1.80 | 1.69 | 0.15 |
| 187 | 10124 | ARL4A | ADP ribosylation factor like GTPase 4A | 1.71 | 1.66 | 1.68 | 0.03 |
| 188 | 221687 | RNF182 | ring finger protein 182 | 1.55 | 1.81 | 1.68 | 0.19 |
| 189 | 1602 | DACH1 | dachshund family transcription factor 1 | 1.60 | 1.75 | 1.67 | 0.11 |
| 190 | 154664 | ABCA13 | ATP binding cassette subfamily A member 13 | 1.82 | 1.51 | 1.67 | 0.22 |
| 191 | 10234 | LRRC17 | leucine rich repeat containing 17 | 1.94 | 1.39 | 1.66 | 0.39 |
| 192 | 11178 | LZTS1 | leucine zipper tumor suppressor 1 | 1.65 | 1.67 | 1.66 | 0.01 |
| 193 | 54508 | EPB41L4A-DT | EPB41L4A antisense RNA 2 (head to head) | 1.72 | 1.61 | 1.66 | 0.08 |
| 194 | 643669 | CCER2 | coiled-coil glutamate rich protein 2 | 2.03 | 1.29 | 1.66 | 0.52 |
| 195 | 100505978 | LOC100505978 | uncharacterized LOC100505978 | 1.74 | 1.55 | 1.65 | 0.13 |
| 196 | 2982 | GUCY1A1 | guanylate cyclase 1 soluble subunit alpha | 1.68 | 1.58 | 1.63 | 0.07 |
| 197 | 10451 | VAV3 | vav guanine nucleotide exchange factor 3 | 1.32 | 1.92 | 1.62 | 0.43 |
| 198 | 100271927 | RASA4B | RAS p21 protein activator 4B | 1.84 | 1.36 | 1.60 | 0.34 |
| 199 | 3815 | KIT | KIT proto-oncogene receptor tyrosine kinase | 1.96 | 1.24 | 1.60 | 0.50 |
| 200 | 161835 | FSIP1 | fibrous sheath interacting protein 1 | 1.92 | 1.26 | 1.59 | 0.47 |
| 201 | 1411 | CRYBA1 | crystallin beta A1 | 1.76 | 1.35 | 1.56 | 0.29 |
| 202 | 1501 | CTNND2 | catenin delta 2 | 1.26 | 1.83 | 1.54 | 0.41 |
| 203 | 57451 | TENM2 | teneurin transmembrane protein 2 | 1.25 | 1.83 | 1.54 | 0.41 |
| 204 | 7133 | TNFRSF1B | TNF receptor superfamily member 1B | 2.05 | 1.03 | 1.54 | 0.72 |
| 205 | 83999 | KREMEN1 | kringle containing transmembrane protein 1 | 1.73 | 1.34 | 1.53 | 0.28 |
| 206 | 6775 | STAT4 | signal transducer and activator of transcription 4 | 1.65 | 1.39 | 1.52 | 0.18 |
| 207 | 56937 | PMEPA1 | prostate transmembrane protein. androgen induced 1 | 1.81 | 1.22 | 1.51 | 0.41 |
| 208 | 1879 | EBF1 | early B-cell factor 1 | 1.71 | 1.30 | 1.50 | 0.29 |
| 209 | 388662 | SLC6A17 | solute carrier family 6 member 17 | 1.36 | 1.63 | 1.49 | 0.19 |
| 210 | 9576 | SPAG6 | sperm associated antigen 6 | 1.62 | 1.36 | 1.49 | 0.18 |
| 211 | 2302 | FOXJ1 | forkhead box J1 | 1.06 | 1.88 | 1.47 | 0.58 |
| 212 | 100131213 | ZNF503-AS2 | ZNF503 antisense RNA 2 | 1.91 | 1.02 | 1.46 | 0.63 |
| 213 | 5454 | POU3F2 | POU class 3 homeobox 2 | 1.28 | 1.62 | 1.45 | 0.24 |
| 214 | 105 | ADARB2 | adenosine deaminase. RNA specific B2 (inactive) | 1.44 | 1.46 | 1.45 | 0.01 |
| 215 | 5992 | RFX4 | regulatory factor X4 | 1.51 | 1.38 | 1.45 | 0.09 |
| 216 | 81553 | FAM49A | family with sequence similarity 49 member A | 1.40 | 1.46 | 1.43 | 0.04 |
| 217 | 100505865 | LINC00920 | long intergenic non-protein coding RNA 920 | 1.27 | 1.58 | 1.42 | 0.22 |
| 218 | 63895 | PIEZO2 | piezo type mechanosensitive ion channel component 2 | 1.45 | 1.39 | 1.42 | 0.04 |
| 219 | 861 | RUNX1 | runt related transcription factor 1 | 1.40 | 1.44 | 1.42 | 0.03 |
| 220 | 8507 | ENC1 | ectodermal-neural cortex 1 | 1.27 | 1.57 | 1.42 | 0.21 |
| 221 | 2619 | GAS1 | growth arrest specific 1 | 1.70 | 1.11 | 1.41 | 0.41 |
| 222 | 22836 | RHOBTB3 | Rho related BTB domain containing 3 | 1.72 | 1.06 | 1.39 | 0.47 |
| 223 | 441094 | NR2F1-AS1 | NR2F1 antisense RNA 1 | 1.43 | 1.35 | 1.39 | 0.06 |
| 224 | 100505668 | N/A | N/A | 1.73 | 1.03 | 1.38 | 0.49 |
| 225 | 6263 | RYR3 | ryanodine receptor 3 | 1.73 | 1.02 | 1.38 | 0.50 |
| 226 | 1993 | ELAVL2 | ELAV like RNA binding protein 2 | 1.46 | 1.30 | 1.38 | 0.12 |
| 227 | 25825 | BACE2 | beta-site APP-cleaving enzyme 2 | 1.68 | 1.07 | 1.38 | 0.43 |
| 228 | 658 | BMPR1B | bone morphogenetic protein receptor type 1B | 1.64 | 1.11 | 1.37 | 0.37 |
| 229 | 81832 | NETO1 | neuropilin and tolloid like 1 | 1.29 | 1.43 | 1.36 | 0.10 |
| 230 | 375057 | STUM | stum. mechanosensory transduction mediator homolog | 1.56 | 1.17 | 1.36 | 0.28 |
| 231 | 1870 | E2F2 | E2F transcription factor 2 | 1.40 | 1.30 | 1.35 | 0.07 |
| 232 | 63901 | FAM111A | family with sequence similarity 111 member A | 1.04 | 1.66 | 1.35 | 0.43 |
| 233 | 84002 | B3GNT5 | UDP-GlcNAc:betaGal beta-1.3-N-acetylglucosaminyltransferase 5 | 1.51 | 1.18 | 1.34 | 0.23 |
| 234 | 11069 | RAPGEF4 | Rap guanine nucleotide exchange factor 4 | 1.30 | 1.38 | 1.34 | 0.05 |
| 235 | 79191 | IRX3 | iroquois homeobox 3 | 1.44 | 1.24 | 1.34 | 0.14 |
| 236 | 114787 | GPRIN1 | G protein regulated inducer of neurite outgrowth 1 | 1.03 | 1.61 | 1.32 | 0.41 |
| 237 | 54898 | ELOVL2 | ELOVL fatty acid elongase 2 | 1.25 | 1.37 | 1.31 | 0.09 |
| 238 | 9901 | SRGAP3 | SLIT-ROBO Rho GTPase activating protein 3 | 1.36 | 1.23 | 1.30 | 0.09 |
| 239 | 22881 | ANKRD6 | ankyrin repeat domain 6 | 1.10 | 1.49 | 1.30 | 0.28 |
| 240 | 388815 | MIR99AHG | mir-99a-let-7c cluster host gene | 1.25 | 1.33 | 1.29 | 0.06 |
| 241 | 7025 | NR2F1 | nuclear receptor subfamily 2 group F member 1 | 1.35 | 1.22 | 1.29 | 0.09 |
| 242 | 100505501 | LOC100505501 | uncharacterized LOC100505501 | 1.53 | 1.05 | 1.29 | 0.34 |
| 243 | 342979 | PALM3 | paralemmin 3 | 1.37 | 1.19 | 1.28 | 0.13 |
| 244 | 344148 | NCKAP5 | NCK associated protein 5 | 1.10 | 1.47 | 1.28 | 0.26 |
| 245 | 57540 | DISP3 | dispatched RND transporter family member 3 | 1.18 | 1.38 | 1.28 | 0.14 |
| 246 | 4774 | NFIA | nuclear factor I A | 1.40 | 1.15 | 1.27 | 0.18 |
| 247 | 116372 | LYPD1 | LY6/PLAUR domain containing 1 | 1.04 | 1.51 | 1.27 | 0.33 |
| 248 | 7352 | UCP3 | uncoupling protein 3 | 1.12 | 1.40 | 1.26 | 0.20 |
| 249 | 84168 | ANTXR1 | anthrax toxin receptor 1 | 1.08 | 1.44 | 1.26 | 0.26 |
| 250 | 29114 | TAGLN3 | transgelin 3 | 1.28 | 1.20 | 1.24 | 0.06 |
| 251 | 2335 | FN1 | fibronectin 1 | 1.42 | 1.06 | 1.24 | 0.25 |
| 252 | 79173 | C19orf57 | chromosome 19 open reading frame 57 | 1.18 | 1.30 | 1.24 | 0.08 |
| 253 | 84879 | MFSD2A | major facilitator superfamily domain containing 2A | 1.27 | 1.21 | 1.24 | 0.05 |
| 254 | 643911 | CRNDE | colorectal neoplasia differentially expressed (non-protein coding) | 1.22 | 1.24 | 1.23 | 0.01 |
| 255 | 85414 | SLC45A3 | solute carrier family 45 member 3 | 1.45 | 1.01 | 1.23 | 0.31 |
| 256 | 4610 | MYCL | v-myc avian myelocytomatosis viral oncogene lung carcinoma derived homolog | 1.18 | 1.28 | 1.23 | 0.07 |
| 257 | 25927 | CNRIP1 | cannabinoid receptor interacting protein 1 | 1.18 | 1.26 | 1.22 | 0.05 |
| 258 | 3764 | KCNJ8 | potassium voltage-gated channel subfamily J member 8 | 1.24 | 1.14 | 1.19 | 0.08 |
| 259 | 259217 | HSPA12A | heat shock protein family A (Hsp70) member 12A | 1.15 | 1.20 | 1.17 | 0.04 |
| 260 | 55022 | PID1 | phosphotyrosine interaction domain containing 1 | 1.31 | 1.03 | 1.17 | 0.20 |
| 261 | 7057 | THBS1 | thrombospondin 1 | 1.02 | 1.32 | 1.17 | 0.21 |
| 262 | 158326 | FREM1 | FRAS1 related extracellular matrix 1 | 1.17 | 1.13 | 1.15 | 0.03 |
| 263 | 146857 | SLFN13 | schlafen family member 13 | 1.16 | 1.14 | 1.15 | 0.02 |
| 264 | 100130155 | MIR124-2HG | MIR124-2 host gene | 1.03 | 1.27 | 1.15 | 0.17 |
| 265 | 9480 | ONECUT2 | one cut homeobox 2 | 1.08 | 1.18 | 1.13 | 0.07 |
| 266 | 23555 | TSPAN15 | tetraspanin 15 | 1.16 | 1.09 | 1.12 | 0.05 |
| 267 | 147650 | SPACA6 | sperm acrosome associated 6 | 1.05 | 1.18 | 1.12 | 0.09 |
| 268 | 100505738 | MIR4458HG | MIR4458 host gene | 1.20 | 1.03 | 1.12 | 0.12 |
| 269 | 26053 | AUTS2 | autism susceptibility candidate 2 | 1.18 | 1.03 | 1.10 | 0.11 |
| 270 | 8313 | AXIN2 | axin 2 | 1.19 | 1.01 | 1.10 | 0.13 |
| 271 | 50487 | PLA2G3 | phospholipase A2 group III | 1.07 | 1.09 | 1.08 | 0.01 |
| 272 | 2674 | GFRA1 | GDNF family receptor alpha 1 | 1.11 | 1.03 | 1.07 | 0.06 |
| 273 | 4185 | ADAM11 | ADAM metallopeptidase domain 11 | 1.09 | 1.05 | 1.07 | 0.03 |
