# Supplementary material for: Investigation of de novo mutations in a schizophrenia case-parent trio by induced pluripotent stem cell-based in vitro disease modeling: convergence of schizophrenia- and autism-related cellular phenotypes
Source: Stem Cell Res Ther. 2020 Nov 27;11:504. doi: 10.1186/s13287-020-01980-5 (PMC7694414; doi:10.1186/s13287-020-01980-5)
Supplement: Supplementary file 7 — Additional file 7: Supplementary Table 2. List of downregulated DE genes in NPC-SZ-HU-PROB compared to both NPC-SZ-HU-FA and NPC-SZ-HU-MO. [file 13287_2020_1980_MOESM7_ESM.docx]

**Supplementary Table 2. List of downregulated DE genes in NPC-SZ-HU-PROB compared to both NPC-SZ-HU-FA and NPC-SZ-HU-MO.**

**N/A = non-applicable**

| ORDER | ENTREZ ID | SYMBOL | NAME | NPC-SZ-HU-PROB/NPC-SZ-HU-FA LOGFC | NPC-SZ-HU-PROB/NPC-SZ-HU-MO LOGFC | AVG LOGFC | STD |
| --- | --- | --- | --- | --- | --- | --- | --- |
| 1 | 286133 | SCARA5 | scavenger receptor class A member 5 | -6.73 | -6.44 | -6.59 | 0.20 |
| 2 | 2267 | FGL1 | fibrinogen like 1 | -6.56 | -4.67 | -5.61 | 1.34 |
| 3 | 6997 | TDGF1 | teratocarcinoma-derived growth factor 1 | -5.43 | -5.75 | -5.59 | 0.22 |
| 4 | 594857 | NPS | neuropeptide S | -5.19 | -5.66 | -5.43 | 0.33 |
| 5 | 387129 | NPSR1 | neuropeptide S receptor 1 | -7.13 | -3.66 | -5.39 | 2.45 |
| 6 | 642587 | MIR205HG | MIR205 host gene | -4.98 | -5.50 | -5.24 | 0.37 |
| 7 | 442425 | FOXB2 | forkhead box B2 | -5.30 | -5.17 | -5.24 | 0.09 |
| 8 | 978 | CDA | cytidine deaminase | -6.54 | -3.93 | -5.24 | 1.84 |
| 9 | 3684 | ITGAM | integrin subunit alpha M | -4.77 | -5.66 | -5.21 | 0.62 |
| 10 | 170850 | KCNG3 | potassium voltage-gated channel modifier subfamily G member 3 | -6.42 | -3.99 | -5.21 | 1.72 |
| 11 | 199713 | NLRP7 | NLR family pyrin domain containing 7 | -4.96 | -5.41 | -5.19 | 0.32 |
| 12 | 100506891 | N/A | N/A | -6.13 | -4.14 | -5.13 | 1.41 |
| 13 | 503582 | ARGFX | arginine-fifty homeobox | -5.15 | -4.95 | -5.05 | 0.14 |
| 14 | 2249 | FGF4 | fibroblast growth factor 4 | -5.69 | -4.42 | -5.05 | 0.90 |
| 15 | 222584 | FAM83B | family with sequence similarity 83 member B | -5.77 | -4.28 | -5.02 | 1.05 |
| 16 | 347694 | ECEL1P2 | endothelin converting enzyme like 1 pseudogene 2 | -5.61 | -4.31 | -4.96 | 0.92 |
| 17 | 6751 | SSTR1 | somatostatin receptor 1 | -4.85 | -5.00 | -4.93 | 0.11 |
| 18 | 100505817 | LINC02582 | uncharacterized LOC100505817 | -5.79 | -4.03 | -4.91 | 1.24 |
| 19 | 344758 | GPR149 | G protein-coupled receptor 149 | -6.28 | -3.34 | -4.81 | 2.08 |
| 20 | 29947 | DNMT3L | DNA methyltransferase 3 like | -4.74 | -4.85 | -4.79 | 0.08 |
| 21 | 1814 | DRD3 | dopamine receptor D3 | -4.28 | -5.29 | -4.78 | 0.71 |
| 22 | 27287 | VENTX | VENT homeobox | -4.94 | -4.53 | -4.74 | 0.29 |
| 23 | 282618 | IFNL1 | interferon lambda 1 | -5.74 | -3.70 | -4.72 | 1.44 |
| 24 | 8433 | UTF1 | undifferentiated embryonic cell transcription factor 1 | -4.94 | -4.37 | -4.65 | 0.40 |
| 25 | 79923 | NANOG | Nanog homeobox | -4.41 | -4.72 | -4.57 | 0.22 |
| 26 | 251 | ALPG | alkaline phosphatase. placental like 2 | -4.61 | -4.52 | -4.57 | 0.06 |
| 27 | 94025 | MUC16 | mucin 16. cell surface associated | -5.70 | -3.39 | -4.55 | 1.63 |
| 28 | 790952 | ESRG | embryonic stem cell related (non-protein coding) | -4.60 | -4.45 | -4.53 | 0.10 |
| 29 | 8224 | SYN3 | synapsin III | -4.80 | -4.07 | -4.44 | 0.52 |
| 30 | 79152 | FA2H | fatty acid 2-hydroxylase | -4.59 | -4.25 | -4.42 | 0.24 |
| 31 | 29785 | CYP2S1 | cytochrome P450 family 2 subfamily S member 1 | -4.73 | -4.07 | -4.40 | 0.46 |
| 32 | 404744 | NPSR1-AS1 | NPSR1 antisense RNA 1 | -4.98 | -3.73 | -4.36 | 0.89 |
| 33 | 9413 | FAM189A2 | family with sequence similarity 189 member A2 | -5.21 | -3.46 | -4.33 | 1.24 |
| 34 | 1618 | DAZL | deleted in azoospermia like | -6.14 | -2.53 | -4.33 | 2.55 |
| 35 | 8928 | FOXH1 | forkhead box H1 | -4.82 | -3.75 | -4.29 | 0.75 |
| 36 | 167127 | UGT3A2 | UDP glycosyltransferase family 3 member A2 | -5.29 | -3.28 | -4.28 | 1.42 |
| 37 | 205860 | TRIML2 | tripartite motif family like 2 | -4.78 | -3.78 | -4.28 | 0.71 |
| 38 | 100507511 | N/A | N/A | -4.88 | -3.65 | -4.27 | 0.87 |
| 39 | 3172 | HNF4A | hepatocyte nuclear factor 4 alpha | -5.92 | -2.57 | -4.25 | 2.37 |
| 40 | 100506389 | N/A | N/A | -5.20 | -3.29 | -4.24 | 1.35 |
| 41 | 9074 | CLDN6 | claudin 6 | -4.83 | -3.64 | -4.23 | 0.84 |
| 42 | 250 | ALPP | alkaline phosphatase. placental | -4.45 | -3.96 | -4.21 | 0.35 |
| 43 | 338596 | ST8SIA6 | ST8 alpha-N-acetyl-neuraminide alpha-2.8-sialyltransferase 6 | -5.58 | -2.81 | -4.19 | 1.96 |
| 44 | 283303 | MRGPRG-AS1 | MRGPRG antisense RNA 1 | -5.77 | -2.54 | -4.16 | 2.29 |
| 45 | 2558 | GABRA5 | gamma-aminobutyric acid type A receptor alpha5 subunit | -4.79 | -3.52 | -4.16 | 0.90 |
| 46 | 6581 | SLC22A3 | solute carrier family 22 member 3 | -4.18 | -4.12 | -4.15 | 0.04 |
| 47 | 10086 | HHLA1 | HERV-H LTR-associating 1 | -4.41 | -3.89 | -4.15 | 0.37 |
| 48 | 100507278 | N/A | N/A | -4.55 | -3.74 | -4.14 | 0.57 |
| 49 | 100506647 | N/A | N/A | -3.90 | -4.38 | -4.14 | 0.34 |
| 50 | 117245 | PLAAT5 | HRAS like suppressor family member 5 | -4.36 | -3.91 | -4.14 | 0.32 |
| 51 | 129025 | ZNF280A | zinc finger protein 280A | -6.19 | -2.07 | -4.13 | 2.91 |
| 52 | 51725 | FBXO40 | F-box protein 40 | -4.46 | -3.75 | -4.11 | 0.50 |
| 53 | 391712 | TRIM61 | tripartite motif containing 61 | -4.69 | -3.51 | -4.10 | 0.84 |
| 54 | 3352 | HTR1D | 5-hydroxytryptamine receptor 1D | -4.44 | -3.75 | -4.10 | 0.49 |
| 55 | 7478 | WNT8A | Wnt family member 8A | -4.54 | -3.62 | -4.08 | 0.65 |
| 56 | 54596 | L1TD1 | LINE1 type transposase domain containing 1 | -4.24 | -3.91 | -4.07 | 0.23 |
| 57 | 1804 | DPP6 | dipeptidyl peptidase like 6 | -3.42 | -4.67 | -4.05 | 0.88 |
| 58 | 6518 | SLC2A5 | solute carrier family 2 member 5 | -4.37 | -3.67 | -4.02 | 0.50 |
| 59 | 359787 | DPPA3 | developmental pluripotency associated 3 | -3.36 | -4.67 | -4.01 | 0.92 |
| 60 | 200150 | PLD5 | phospholipase D family member 5 | -6.28 | -1.74 | -4.01 | 3.21 |
| 61 | 346689 | KLRG2 | killer cell lectin like receptor G2 | -4.55 | -3.44 | -3.99 | 0.78 |
| 62 | 56660 | KCNK12 | potassium two pore domain channel subfamily K member 12 | -4.57 | -3.41 | -3.99 | 0.82 |
| 63 | 943 | TNFRSF8 | TNF receptor superfamily member 8 | -4.28 | -3.68 | -3.98 | 0.42 |
| 64 | 6035 | RNASE1 | ribonuclease A family member 1. pancreatic | -5.19 | -2.76 | -3.98 | 1.72 |
| 65 | 650368 | TSSC2 | tumor suppressing subtransferable candidate 2 pseudogene | -3.60 | -4.28 | -3.94 | 0.48 |
| 66 | 3982 | LIM2 | lens intrinsic membrane protein 2 | -5.09 | -2.74 | -3.91 | 1.66 |
| 67 | 2700 | GJA3 | gap junction protein alpha 3 | -5.09 | -2.73 | -3.91 | 1.67 |
| 68 | 7225 | TRPC6 | transient receptor potential cation channel subfamily C member 6 | -4.16 | -3.62 | -3.89 | 0.38 |
| 69 | 100507127 | LINC00707 | long intergenic non-protein coding RNA 707 | -4.80 | -2.97 | -3.89 | 1.29 |
| 70 | 55244 | SLC47A1 | solute carrier family 47 member 1 | -4.11 | -3.64 | -3.88 | 0.34 |
| 71 | 84891 | ZSCAN10 | zinc finger and SCAN domain containing 10 | -3.68 | -4.07 | -3.87 | 0.28 |
| 72 | 100132916 | SHISAL2B | family with sequence similarity 159 member B | -5.41 | -2.32 | -3.86 | 2.19 |
| 73 | 338339 | CLEC4D | C-type lectin domain family 4 member D | -4.73 | -2.98 | -3.86 | 1.23 |
| 74 | 9215 | LARGE1 | LARGE xylosyl- and glucuronyltransferase 1 | -4.25 | -3.44 | -3.84 | 0.57 |
| 75 | 1379 | CR1L | complement C3b/C4b receptor 1 like | -4.07 | -3.61 | -3.84 | 0.32 |
| 76 | 1117 | CHI3L2 | chitinase 3 like 2 | -4.88 | -2.79 | -3.83 | 1.48 |
| 77 | 9314 | KLF4 | Kruppel like factor 4 | -4.13 | -3.53 | -3.83 | 0.42 |
| 78 | 647166 | C13orf42 | long intergenic non-protein coding RNA 371 | -4.54 | -3.10 | -3.82 | 1.02 |
| 79 | 7490 | WT1 | Wilms tumor 1 | -4.10 | -3.52 | -3.81 | 0.41 |
| 80 | 326 | AIRE | autoimmune regulator | -3.84 | -3.78 | -3.81 | 0.05 |
| 81 | 7113 | TMPRSS2 | transmembrane protease. serine 2 | -3.73 | -3.82 | -3.78 | 0.06 |
| 82 | 389206 | BEND4 | BEN domain containing 4 | -4.27 | -3.29 | -3.78 | 0.69 |
| 83 | 117156 | SCGB3A2 | secretoglobin family 3A member 2 | -4.06 | -3.48 | -3.77 | 0.41 |
| 84 | 7498 | XDH | xanthine dehydrogenase | -5.57 | -1.95 | -3.76 | 2.56 |
| 85 | 153579 | BTNL9 | butyrophilin like 9 | -3.34 | -4.16 | -3.75 | 0.58 |
| 86 | 4071 | TM4SF1 | transmembrane 4 L six family member 1 | -3.51 | -3.95 | -3.73 | 0.32 |
| 87 | 3872 | KRT17 | keratin 17 | -4.57 | -2.88 | -3.73 | 1.20 |
| 88 | 120071 | LARGE2 | LARGE xylosyl- and glucuronyltransferase 2 | -4.37 | -3.06 | -3.71 | 0.93 |
| 89 | 100169750 | PRINS | psoriasis associated non-protein coding RNA induced by stress | -4.35 | -3.04 | -3.70 | 0.93 |
| 90 | 55237 | VRTN | vertebrae development associated | -4.54 | -2.85 | -3.70 | 1.19 |
| 91 | 7010 | TEK | TEK receptor tyrosine kinase | -4.35 | -2.93 | -3.64 | 1.01 |
| 92 | 3775 | KCNK1 | potassium two pore domain channel subfamily K member 1 | -3.88 | -3.39 | -3.64 | 0.35 |
| 93 | 283875 | LINC00514 | long intergenic non-protein coding RNA 514 | -4.36 | -2.90 | -3.63 | 1.03 |
| 94 | 63978 | PRDM14 | PR/SET domain 14 | -3.72 | -3.50 | -3.61 | 0.15 |
| 95 | 887 | CCKBR | cholecystokinin B receptor | -5.59 | -1.62 | -3.61 | 2.81 |
| 96 | 3489 | IGFBP6 | insulin like growth factor binding protein 6 | -4.03 | -3.15 | -3.59 | 0.62 |
| 97 | 5544 | PRB3 | proline rich protein BstNI subfamily 3 | -4.37 | -2.80 | -3.58 | 1.11 |
| 98 | 54845 | ESRP1 | epithelial splicing regulatory protein 1 | -3.11 | -4.04 | -3.58 | 0.66 |
| 99 | 5624 | PROC | protein C. inactivator of coagulation factors Va and VIIIa | -3.42 | -3.72 | -3.57 | 0.21 |
| 100 | 664701 | ZNF826P | zinc finger protein 826. pseudogene | -3.86 | -3.27 | -3.57 | 0.42 |
| 101 | 642559 | POU5F1P3 | POU class 5 homeobox 1 pseudogene 3 | -3.92 | -3.20 | -3.56 | 0.51 |
| 102 | 4585 | MUC4 | mucin 4. cell surface associated | -3.64 | -3.42 | -3.53 | 0.16 |
| 103 | 91662 | NLRP12 | NLR family pyrin domain containing 12 | -3.60 | -3.43 | -3.51 | 0.12 |
| 104 | 729330 | OC90 | otoconin 90 | -3.70 | -3.33 | -3.51 | 0.27 |
| 105 | 2642 | GCGR | glucagon receptor | -3.64 | -3.38 | -3.51 | 0.18 |
| 106 | 4494 | MT1F | metallothionein 1F | -3.83 | -3.19 | -3.51 | 0.46 |
| 107 | 256691 | MAMDC2 | MAM domain containing 2 | -3.74 | -3.26 | -3.50 | 0.34 |
| 108 | 8854 | ALDH1A2 | aldehyde dehydrogenase 1 family member A2 | -3.56 | -3.35 | -3.46 | 0.15 |
| 109 | 999 | CDH1 | cadherin 1 | -3.67 | -3.23 | -3.45 | 0.31 |
| 110 | 10544 | PROCR | protein C receptor | -3.77 | -3.09 | -3.43 | 0.48 |
| 111 | 29842 | TFCP2L1 | transcription factor CP2 like 1 | -3.66 | -3.20 | -3.43 | 0.32 |
| 112 | 100507150 | N/A | N/A | -3.79 | -3.06 | -3.42 | 0.52 |
| 113 | 256764 | WDR72 | WD repeat domain 72 | -4.06 | -2.76 | -3.41 | 0.92 |
| 114 | 55655 | NLRP2 | NLR family pyrin domain containing 2 | -3.53 | -3.27 | -3.40 | 0.19 |
| 115 | 126669 | SHE | Src homology 2 domain containing E | -3.39 | -3.39 | -3.39 | 0.00 |
| 116 | 8638 | OASL | 2'-5'-oligoadenylate synthetase like | -4.27 | -2.50 | -3.38 | 1.25 |
| 117 | 9623 | TCL1B | T-cell leukemia/lymphoma 1B | -3.68 | -3.08 | -3.38 | 0.43 |
| 118 | 1043 | CD52 | CD52 molecule | -3.58 | -3.14 | -3.36 | 0.31 |
| 119 | 5308 | PITX2 | paired like homeodomain 2 | -3.09 | -3.61 | -3.35 | 0.36 |
| 120 | 56944 | OLFML3 | olfactomedin like 3 | -3.36 | -3.34 | -3.35 | 0.02 |
| 121 | 285401 | LINC00698 | long intergenic non-protein coding RNA 698 | -3.58 | -3.06 | -3.32 | 0.37 |
| 122 | 5462 | POU5F1B | POU class 5 homeobox 1B | -3.68 | -2.96 | -3.32 | 0.51 |
| 123 | 3106 | HLA-B | major histocompatibility complex. class I. B | -3.54 | -3.09 | -3.32 | 0.32 |
| 124 | 8532 | CPZ | carboxypeptidase Z | -3.71 | -2.92 | -3.31 | 0.56 |
| 125 | 100506013 | APELA | apelin receptor early endogenous ligand | -3.23 | -3.38 | -3.31 | 0.11 |
| 126 | 442894 | MIR302B | microRNA 302b | -4.29 | -2.31 | -3.30 | 1.40 |
| 127 | 389634 | LINC00937 | long intergenic non-protein coding RNA 937 | -3.43 | -3.16 | -3.30 | 0.19 |
| 128 | 84935 | MEDAG | mesenteric estrogen dependent adipogenesis | -4.37 | -2.22 | -3.30 | 1.52 |
| 129 | 22899 | ARHGEF15 | Rho guanine nucleotide exchange factor 15 | -4.07 | -2.46 | -3.27 | 1.14 |
| 130 | 54941 | RNF125 | ring finger protein 125 | -3.36 | -3.17 | -3.27 | 0.13 |
| 131 | 2895 | GRID2 | glutamate ionotropic receptor delta type subunit 2 | -3.08 | -3.44 | -3.26 | 0.25 |
| 132 | 84433 | CARD11 | caspase recruitment domain family member 11 | -3.21 | -3.31 | -3.26 | 0.07 |
| 133 | 283897 | C16orf54 | chromosome 16 open reading frame 54 | -2.90 | -3.59 | -3.25 | 0.49 |
| 134 | 339535 | LINC01139 | long intergenic non-protein coding RNA 1139 | -3.41 | -3.08 | -3.24 | 0.23 |
| 135 | 30816 | ERVW-1 | endogenous retrovirus group W member 1 | -3.21 | -3.27 | -3.24 | 0.04 |
| 136 | 100507084 | N/A | N/A | -4.25 | -2.21 | -3.23 | 1.44 |
| 137 | 130399 | ACVR1C | activin A receptor type 1C | -3.79 | -2.68 | -3.23 | 0.79 |
| 138 | 4501 | MT1X | metallothionein 1X | -3.59 | -2.85 | -3.22 | 0.52 |
| 139 | 401262 | CRIP3 | cysteine rich protein 3 | -3.65 | -2.77 | -3.21 | 0.62 |
| 140 | 3248 | HPGD | hydroxyprostaglandin dehydrogenase 15-(NAD) | -2.56 | -3.85 | -3.20 | 0.91 |
| 141 | 100128304 | N/A | N/A | -4.55 | -1.86 | -3.20 | 1.90 |
| 142 | 644150 | WIPF3 | WAS/WASL interacting protein family member 3 | -3.24 | -3.15 | -3.19 | 0.06 |
| 143 | 84691 | FAM71F1 | family with sequence similarity 71 member F1 | -3.54 | -2.85 | -3.19 | 0.49 |
| 144 | 23349 | PHF24 | PHD finger protein 24 | -3.27 | -3.10 | -3.18 | 0.12 |
| 145 | 51314 | NME8 | NME/NM23 family member 8 | -4.14 | -2.21 | -3.18 | 1.36 |
| 146 | 144568 | A2ML1 | alpha-2-macroglobulin like 1 | -3.58 | -2.74 | -3.16 | 0.60 |
| 147 | 5272 | SERPINB9 | serpin family B member 9 | -2.92 | -3.39 | -3.15 | 0.34 |
| 148 | 9124 | PDLIM1 | PDZ and LIM domain 1 | -3.00 | -3.31 | -3.15 | 0.22 |
| 149 | 6424 | SFRP4 | secreted frizzled related protein 4 | -3.90 | -2.39 | -3.15 | 1.07 |
| 150 | 29119 | CTNNA3 | catenin alpha 3 | -3.93 | -2.37 | -3.15 | 1.10 |
| 151 | 2295 | FOXF2 | forkhead box F2 | -2.96 | -3.32 | -3.14 | 0.25 |
| 152 | 55808 | ST6GALNAC1 | ST6 N-acetylgalactosaminide alpha-2.6-sialyltransferase 1 | -3.21 | -3.06 | -3.13 | 0.10 |
| 153 | 154075 | SAMD3 | sterile alpha motif domain containing 3 | -4.50 | -1.75 | -3.13 | 1.94 |
| 154 | 27145 | FILIP1 | filamin A interacting protein 1 | -3.50 | -2.73 | -3.11 | 0.55 |
| 155 | 152 | ADRA2C | adrenoceptor alpha 2C | -3.86 | -2.36 | -3.11 | 1.06 |
| 156 | 7111 | TMOD1 | tropomodulin 1 | -4.14 | -2.08 | -3.11 | 1.46 |
| 157 | 148281 | SYT6 | synaptotagmin 6 | -3.15 | -3.05 | -3.10 | 0.07 |
| 158 | 1366 | CLDN7 | claudin 7 | -3.24 | -2.95 | -3.10 | 0.20 |
| 159 | 3856 | KRT8 | keratin 8 | -3.12 | -3.06 | -3.09 | 0.04 |
| 160 | 3375 | IAPP | islet amyloid polypeptide | -3.43 | -2.75 | -3.09 | 0.48 |
| 161 | 51305 | KCNK9 | potassium two pore domain channel subfamily K member 9 | -4.87 | -1.27 | -3.07 | 2.55 |
| 162 | 6262 | RYR2 | ryanodine receptor 2 | -3.41 | -2.72 | -3.07 | 0.49 |
| 163 | 151888 | BTLA | B and T lymphocyte associated | -3.51 | -2.62 | -3.06 | 0.63 |
| 164 | 3171 | FOXA3 | forkhead box A3 | -3.36 | -2.76 | -3.06 | 0.42 |
| 165 | 970 | CD70 | CD70 molecule | -3.01 | -3.10 | -3.06 | 0.06 |
| 166 | 9370 | ADIPOQ | adiponectin. C1Q and collagen domain containing | -3.87 | -2.21 | -3.04 | 1.18 |
| 167 | 133482 | SLCO6A1 | solute carrier organic anion transporter family member 6A1 | -4.18 | -1.79 | -2.99 | 1.69 |
| 168 | 51090 | PLLP | plasmolipin | -3.03 | -2.93 | -2.98 | 0.07 |
| 169 | 27033 | ZBTB32 | zinc finger and BTB domain containing 32 | -3.70 | -2.24 | -2.97 | 1.04 |
| 170 | 647024 | C6orf132 | chromosome 6 open reading frame 132 | -3.04 | -2.89 | -2.97 | 0.10 |
| 171 | 114769 | CARD16 | caspase recruitment domain family member 16 | -3.71 | -2.21 | -2.96 | 1.06 |
| 172 | 51384 | WNT16 | Wnt family member 16 | -3.43 | -2.48 | -2.96 | 0.67 |
| 173 | 7368 | UGT8 | UDP glycosyltransferase 8 | -3.33 | -2.59 | -2.96 | 0.53 |
| 174 | 400680 | LINC00664 | long intergenic non-protein coding RNA 664 | -2.88 | -3.01 | -2.95 | 0.10 |
| 175 | 440590 | ZYG11A | zyg-11 family member A. cell cycle regulator | -3.27 | -2.62 | -2.95 | 0.46 |
| 176 | 645682 | POU5F1P4 | POU class 5 homeobox 1 pseudogene 4 | -3.30 | -2.54 | -2.92 | 0.53 |
| 177 | 338645 | LUZP2 | leucine zipper protein 2 | -3.22 | -2.62 | -2.92 | 0.42 |
| 178 | 115265 | DDIT4L | DNA damage inducible transcript 4 like | -4.45 | -1.39 | -2.92 | 2.16 |
| 179 | 115572 | TENT5B | family with sequence similarity 46 member B | -2.93 | -2.90 | -2.91 | 0.02 |
| 180 | 57126 | CD177 | CD177 molecule | -4.31 | -1.49 | -2.90 | 2.00 |
| 181 | 1007 | CDH9 | cadherin 9 | -3.13 | -2.67 | -2.90 | 0.32 |
| 182 | 5154 | PDGFA | platelet derived growth factor subunit A | -3.00 | -2.74 | -2.87 | 0.18 |
| 183 | 23533 | PIK3R5 | phosphoinositide-3-kinase regulatory subunit 5 | -3.30 | -2.44 | -2.87 | 0.61 |
| 184 | 6690 | SPINK1 | serine peptidase inhibitor. Kazal type 1 | -2.72 | -3.01 | -2.86 | 0.21 |
| 185 | 240 | ALOX5 | arachidonate 5-lipoxygenase | -3.92 | -1.78 | -2.85 | 1.51 |
| 186 | 55811 | ADCY10 | adenylate cyclase 10. soluble | -2.96 | -2.67 | -2.81 | 0.20 |
| 187 | 11131 | CAPN11 | calpain 11 | -3.01 | -2.57 | -2.79 | 0.31 |
| 188 | 55612 | FERMT1 | fermitin family member 1 | -3.08 | -2.47 | -2.77 | 0.43 |
| 189 | 79727 | LIN28A | lin-28 homolog A | -3.26 | -2.29 | -2.77 | 0.69 |
| 190 | 25791 | NGEF | neuronal guanine nucleotide exchange factor | -3.47 | -2.07 | -2.77 | 0.99 |
| 191 | 116285 | ACSM1 | acyl-CoA synthetase medium-chain family member 1 | -2.86 | -2.68 | -2.77 | 0.13 |
| 192 | 1365 | CLDN3 | claudin 3 | -3.93 | -1.60 | -2.76 | 1.64 |
| 193 | 344167 | FOXI3 | forkhead box I3 | -2.53 | -2.96 | -2.75 | 0.31 |
| 194 | 9830 | TRIM14 | tripartite motif containing 14 | -2.85 | -2.64 | -2.75 | 0.15 |
| 195 | 100130921 | N/A | N/A | -3.24 | -2.23 | -2.73 | 0.71 |
| 196 | 135398 | C6orf141 | chromosome 6 open reading frame 141 | -3.10 | -2.36 | -2.73 | 0.53 |
| 197 | 153562 | MARVELD2 | MARVEL domain containing 2 | -3.82 | -1.63 | -2.73 | 1.55 |
| 198 | 26034 | IPCEF1 | interaction protein for cytohesin exchange factors 1 | -2.74 | -2.71 | -2.72 | 0.03 |
| 199 | 5865 | RAB3B | RAB3B. member RAS oncogene family | -2.70 | -2.74 | -2.72 | 0.03 |
| 200 | 22795 | NID2 | nidogen 2 | -3.07 | -2.36 | -2.72 | 0.50 |
| 201 | 25769 | SLC24A2 | solute carrier family 24 member 2 | -2.92 | -2.51 | -2.71 | 0.29 |
| 202 | 118788 | PIK3AP1 | phosphoinositide-3-kinase adaptor protein 1 | -2.84 | -2.57 | -2.71 | 0.19 |
| 203 | 167826 | OLIG3 | oligodendrocyte transcription factor 3 | -2.82 | -2.59 | -2.70 | 0.16 |
| 204 | 27134 | TJP3 | tight junction protein 3 | -3.21 | -2.18 | -2.70 | 0.73 |
| 205 | 641364 | SLC7A11-AS1 | SLC7A11 antisense RNA 1 | -2.82 | -2.57 | -2.70 | 0.18 |
| 206 | 51599 | LSR | lipolysis stimulated lipoprotein receptor | -3.15 | -2.23 | -2.69 | 0.65 |
| 207 | 1535 | CYBA | cytochrome b-245 alpha chain | -3.17 | -2.19 | -2.68 | 0.70 |
| 208 | 399829 | LINC01168 | long intergenic non-protein coding RNA 1168 | -3.51 | -1.84 | -2.68 | 1.18 |
| 209 | 57471 | ERMN | ermin | -3.69 | -1.65 | -2.67 | 1.44 |
| 210 | 441355 | MIR2052HG | MIR2052 host gene | -3.54 | -1.78 | -2.66 | 1.24 |
| 211 | 793 | CALB1 | calbindin 1 | -3.60 | -1.72 | -2.66 | 1.33 |
| 212 | 202374 | STK32A | serine/threonine kinase 32A | -3.65 | -1.64 | -2.64 | 1.43 |
| 213 | 219293 | ATAD3C | ATPase family. AAA domain containing 3C | -2.49 | -2.80 | -2.64 | 0.22 |
| 214 | 375519 | GJB7 | gap junction protein beta 7 | -3.03 | -2.25 | -2.64 | 0.55 |
| 215 | 2348 | FOLR1 | folate receptor 1 | -3.87 | -1.41 | -2.64 | 1.73 |
| 216 | 1413 | CRYBA4 | crystallin beta A4 | -2.97 | -2.31 | -2.64 | 0.47 |
| 217 | 762 | CA4 | carbonic anhydrase 4 | -3.38 | -1.89 | -2.64 | 1.05 |
| 218 | 4146 | MATN1 | matrilin 1. cartilage matrix protein | -3.22 | -2.05 | -2.64 | 0.83 |
| 219 | 2919 | CXCL1 | C-X-C motif chemokine ligand 1 | -3.10 | -2.15 | -2.62 | 0.67 |
| 220 | 64478 | CSMD1 | CUB and Sushi multiple domains 1 | -2.78 | -2.46 | -2.62 | 0.22 |
| 221 | 222171 | PRR15 | proline rich 15 | -3.12 | -2.11 | -2.61 | 0.71 |
| 222 | 390010 | NKX1-2 | NK1 homeobox 2 | -3.04 | -2.18 | -2.61 | 0.61 |
| 223 | 100505880 | N/A | N/A | -3.64 | -1.56 | -2.60 | 1.47 |
| 224 | 144195 | SLC2A14 | solute carrier family 2 member 14 | -2.58 | -2.62 | -2.60 | 0.03 |
| 225 | 134285 | TMEM171 | transmembrane protein 171 | -3.64 | -1.56 | -2.60 | 1.47 |
| 226 | 3875 | KRT18 | keratin 18 | -2.50 | -2.69 | -2.60 | 0.14 |
| 227 | 493812 | HCG11 | HLA complex group 11 (non-protein coding) | -3.63 | -1.56 | -2.59 | 1.46 |
| 228 | 344787 | ZNF860 | zinc finger protein 860 | -3.43 | -1.75 | -2.59 | 1.19 |
| 229 | 729085 | GASK1A | family with sequence similarity 198 member A | -3.56 | -1.61 | -2.58 | 1.38 |
| 230 | 639 | PRDM1 | PR/SET domain 1 | -2.33 | -2.83 | -2.58 | 0.35 |
| 231 | 2118 | ETV4 | ETS variant 4 | -2.34 | -2.82 | -2.58 | 0.34 |
| 232 | 3433 | IFIT2 | interferon induced protein with tetratricopeptide repeats 2 | -3.27 | -1.87 | -2.57 | 0.99 |
| 233 | 57569 | ARHGAP20 | Rho GTPase activating protein 20 | -2.78 | -2.33 | -2.56 | 0.32 |
| 234 | 55289 | ACOXL | acyl-CoA oxidase-like | -2.25 | -2.86 | -2.55 | 0.43 |
| 235 | 6387 | CXCL12 | C-X-C motif chemokine ligand 12 | -2.40 | -2.70 | -2.55 | 0.21 |
| 236 | 203328 | SUSD3 | sushi domain containing 3 | -2.25 | -2.84 | -2.55 | 0.42 |
| 237 | 8744 | TNFSF9 | tumor necrosis factor superfamily member 9 | -2.44 | -2.65 | -2.54 | 0.15 |
| 238 | 11005 | SPINK5 | serine peptidase inhibitor. Kazal type 5 | -2.92 | -2.15 | -2.54 | 0.55 |
| 239 | 1131 | CHRM3 | cholinergic receptor muscarinic 3 | -2.91 | -2.16 | -2.53 | 0.53 |
| 240 | 84803 | GPAT3 | glycerol-3-phosphate acyltransferase 3 | -2.12 | -2.92 | -2.52 | 0.57 |
| 241 | 4939 | OAS2 | 2'-5'-oligoadenylate synthetase 2 | -3.15 | -1.89 | -2.52 | 0.89 |
| 242 | 2650 | GCNT1 | glucosaminyl (N-acetyl) transferase 1. core 2 | -3.12 | -1.91 | -2.52 | 0.85 |
| 243 | 115749 | C12orf56 | chromosome 12 open reading frame 56 | -2.66 | -2.34 | -2.50 | 0.22 |
| 244 | 7643 | ZNF90 | zinc finger protein 90 | -1.97 | -3.02 | -2.50 | 0.74 |
| 245 | 1515 | CTSV | cathepsin V | -2.83 | -2.16 | -2.50 | 0.48 |
| 246 | 100506658 | OCLN | occludin | -3.73 | -1.26 | -2.50 | 1.75 |
| 247 | 83878 | USHBP1 | USH1 protein network component harmonin binding protein 1 | -2.70 | -2.28 | -2.49 | 0.29 |
| 248 | 5420 | PODXL | podocalyxin like | -2.72 | -2.23 | -2.47 | 0.35 |
| 249 | 23166 | STAB1 | stabilin 1 | -3.24 | -1.70 | -2.47 | 1.09 |
| 250 | 1414 | CRYBB1 | crystallin beta B1 | -2.87 | -2.06 | -2.46 | 0.57 |
| 251 | 51700 | CYB5R2 | cytochrome b5 reductase 2 | -2.30 | -2.61 | -2.46 | 0.22 |
| 252 | 441307 | HRAT92 | heart tissue-associated transcript 92 | -2.54 | -2.37 | -2.45 | 0.12 |
| 253 | 59341 | TRPV4 | transient receptor potential cation channel subfamily V member 4 | -2.25 | -2.62 | -2.44 | 0.27 |
| 254 | 718 | C3 | complement C3 | -2.02 | -2.82 | -2.42 | 0.57 |
| 255 | 8092 | ALX1 | ALX homeobox 1 | -2.55 | -2.29 | -2.42 | 0.19 |
| 256 | 2697 | GJA1 | gap junction protein alpha 1 | -2.49 | -2.35 | -2.42 | 0.10 |
| 257 | 4233 | MET | MET proto-oncogene. receptor tyrosine kinase | -2.83 | -2.00 | -2.42 | 0.59 |
| 258 | 645367 | GGT8P | gamma-glutamyltransferase 8 pseudogene | -2.76 | -2.07 | -2.42 | 0.49 |
| 259 | 27063 | ANKRD1 | ankyrin repeat domain 1 | -3.25 | -1.56 | -2.40 | 1.19 |
| 260 | 93099 | DMKN | dermokine | -1.95 | -2.85 | -2.40 | 0.64 |
| 261 | 4645 | MYO5B | myosin VB | -2.58 | -2.21 | -2.40 | 0.26 |
| 262 | 149461 | CLDN19 | claudin 19 | -3.15 | -1.64 | -2.40 | 1.06 |
| 263 | 7450 | VWF | von Willebrand factor | -3.51 | -1.27 | -2.39 | 1.59 |
| 264 | 6442 | SGCA | sarcoglycan alpha | -2.83 | -1.94 | -2.39 | 0.62 |
| 265 | 51191 | HERC5 | HECT and RLD domain containing E3 ubiquitin protein ligase 5 | -2.93 | -1.81 | -2.37 | 0.79 |
| 266 | 187 | APLNR | apelin receptor | -2.19 | -2.55 | -2.37 | 0.25 |
| 267 | 2264 | FGFR4 | fibroblast growth factor receptor 4 | -2.46 | -2.26 | -2.36 | 0.14 |
| 268 | 60675 | PROK2 | prokineticin 2 | -2.57 | -2.15 | -2.36 | 0.30 |
| 269 | 168667 | BMPER | BMP binding endothelial regulator | -3.06 | -1.67 | -2.36 | 0.98 |
| 270 | 7022 | TFAP2C | transcription factor AP-2 gamma | -2.05 | -2.66 | -2.35 | 0.43 |
| 271 | 728591 | CCDC169 | coiled-coil domain containing 169 | -2.59 | -2.09 | -2.34 | 0.35 |
| 272 | 100507173 | LINC01012 | long intergenic non-protein coding RNA 1012 | -3.12 | -1.54 | -2.33 | 1.12 |
| 273 | 164668 | APOBEC3H | apolipoprotein B mRNA editing enzyme catalytic subunit 3H | -2.78 | -1.87 | -2.33 | 0.65 |
| 274 | 6275 | S100A4 | S100 calcium binding protein A4 | -2.87 | -1.77 | -2.32 | 0.78 |
| 275 | 4295 | MLN | motilin | -2.01 | -2.62 | -2.31 | 0.43 |
| 276 | 924 | CD7 | CD7 molecule | -2.25 | -2.37 | -2.31 | 0.08 |
| 277 | 495 | ATP4A | ATPase H+/K+ transporting alpha subunit | -2.85 | -1.75 | -2.30 | 0.78 |
| 278 | 3560 | IL2RB | interleukin 2 receptor subunit beta | -2.07 | -2.52 | -2.29 | 0.32 |
| 279 | 1396 | CRIP1 | cysteine rich protein 1 | -2.90 | -1.68 | -2.29 | 0.86 |
| 280 | 221002 | RASGEF1A | RasGEF domain family member 1A | -2.37 | -2.20 | -2.28 | 0.12 |
| 281 | 11145 | PLAAT3 | phospholipase A2 group XVI | -2.07 | -2.48 | -2.28 | 0.29 |
| 282 | 5915 | RARB | retinoic acid receptor beta | -1.62 | -2.93 | -2.27 | 0.93 |
| 283 | 121601 | ANO4 | anoctamin 4 | -2.14 | -2.40 | -2.27 | 0.18 |
| 284 | 2562 | GABRB3 | gamma-aminobutyric acid type A receptor beta3 subunit | -2.47 | -2.06 | -2.26 | 0.29 |
| 285 | 1604 | CD55 | CD55 molecule (Cromer blood group) | -2.31 | -2.21 | -2.26 | 0.07 |
| 286 | 108 | ADCY2 | adenylate cyclase 2 | -2.36 | -2.15 | -2.25 | 0.15 |
| 287 | 1299 | COL9A3 | collagen type IX alpha 3 chain | -2.18 | -2.32 | -2.25 | 0.10 |
| 288 | 197257 | LDHD | lactate dehydrogenase D | -2.40 | -2.09 | -2.25 | 0.22 |
| 289 | 8792 | TNFRSF11A | TNF receptor superfamily member 11a | -2.44 | -2.04 | -2.24 | 0.28 |
| 290 | 348 | APOE | apolipoprotein E | -2.45 | -2.03 | -2.24 | 0.29 |
| 291 | 4306 | NR3C2 | nuclear receptor subfamily 3 group C member 2 | -2.64 | -1.82 | -2.23 | 0.58 |
| 292 | 55214 | P3H2 | prolyl 3-hydroxylase 2 | -2.72 | -1.72 | -2.22 | 0.71 |
| 293 | 26153 | KIF26A | kinesin family member 26A | -2.42 | -2.01 | -2.22 | 0.29 |
| 294 | 145447 | ABHD12B | abhydrolase domain containing 12B | -1.50 | -2.93 | -2.22 | 1.02 |
| 295 | 65988 | ZNF747 | zinc finger protein 747 | -2.95 | -1.48 | -2.21 | 1.04 |
| 296 | 1832 | DSP | desmoplakin | -2.85 | -1.56 | -2.20 | 0.91 |
| 297 | 4145 | MATK | megakaryocyte-associated tyrosine kinase | -2.08 | -2.31 | -2.20 | 0.16 |
| 298 | 55240 | STEAP3 | STEAP3 metalloreductase | -2.43 | -1.96 | -2.20 | 0.33 |
| 299 | 8614 | STC2 | stanniocalcin 2 | -2.35 | -2.04 | -2.19 | 0.22 |
| 300 | 23657 | SLC7A11 | solute carrier family 7 member 11 | -2.49 | -1.89 | -2.19 | 0.43 |
| 301 | 162466 | PHOSPHO1 | phosphoethanolamine/phosphocholine phosphatase | -2.42 | -1.96 | -2.19 | 0.33 |
| 302 | 341 | APOC1 | apolipoprotein C1 | -2.71 | -1.67 | -2.19 | 0.74 |
| 303 | 1674 | DES | desmin | -2.04 | -2.33 | -2.18 | 0.20 |
| 304 | 256987 | SERINC5 | serine incorporator 5 | -2.18 | -2.17 | -2.18 | 0.01 |
| 305 | 728780 | ANKDD1B | ankyrin repeat and death domain containing 1B | -2.35 | -1.99 | -2.17 | 0.26 |
| 306 | 3673 | ITGA2 | integrin subunit alpha 2 | -2.82 | -1.52 | -2.17 | 0.92 |
| 307 | 3351 | HTR1B | 5-hydroxytryptamine receptor 1B | -2.56 | -1.77 | -2.17 | 0.56 |
| 308 | 339366 | ADAMTSL5 | ADAMTS like 5 | -2.28 | -2.05 | -2.16 | 0.17 |
| 309 | 5733 | PTGER3 | prostaglandin E receptor 3 | -2.96 | -1.36 | -2.16 | 1.13 |
| 310 | 2903 | GRIN2A | glutamate ionotropic receptor NMDA type subunit 2A | -2.67 | -1.64 | -2.16 | 0.73 |
| 311 | 6710 | SPTB | spectrin beta. erythrocytic | -2.45 | -1.85 | -2.15 | 0.42 |
| 312 | 2152 | F3 | coagulation factor III. tissue factor | -2.14 | -2.16 | -2.15 | 0.02 |
| 313 | 149775 | GNAS-AS1 | GNAS antisense RNA 1 | -1.86 | -2.44 | -2.15 | 0.41 |
| 314 | 51225 | ABI3 | ABI family member 3 | -2.04 | -2.23 | -2.14 | 0.13 |
| 315 | 645321 | LINC01896 | uncharacterized LOC645321 | -2.02 | -2.25 | -2.14 | 0.16 |
| 316 | 51252 | FAM178B | family with sequence similarity 178 member B | -2.56 | -1.70 | -2.13 | 0.61 |
| 317 | 159963 | SLC5A12 | solute carrier family 5 member 12 | -2.66 | -1.60 | -2.13 | 0.74 |
| 318 | 2324 | FLT4 | fms related tyrosine kinase 4 | -2.40 | -1.86 | -2.13 | 0.38 |
| 319 | 84282 | RNF135 | ring finger protein 135 | -2.80 | -1.46 | -2.13 | 0.94 |
| 320 | 646658 | SYNDIG1L | synapse differentiation inducing 1 like | -2.54 | -1.70 | -2.12 | 0.59 |
| 321 | 114884 | OSBPL10 | oxysterol binding protein like 10 | -2.54 | -1.70 | -2.12 | 0.59 |
| 322 | 100507098 | ADAMTS9-AS2 | ADAMTS9 antisense RNA 2 | -2.50 | -1.74 | -2.12 | 0.54 |
| 323 | 2624 | GATA2 | GATA binding protein 2 | -2.23 | -2.00 | -2.12 | 0.16 |
| 324 | 81607 | NECTIN4 | nectin cell adhesion molecule 4 | -2.30 | -1.93 | -2.12 | 0.27 |
| 325 | 2886 | GRB7 | growth factor receptor bound protein 7 | -1.25 | -2.99 | -2.12 | 1.23 |
| 326 | 84451 | MAP3K21 | mixed lineage kinase 4 | -2.53 | -1.70 | -2.12 | 0.59 |
| 327 | 168002 | DACT2 | dishevelled binding antagonist of beta catenin 2 | -2.05 | -2.17 | -2.11 | 0.08 |
| 328 | 5588 | PRKCQ | protein kinase C theta | -2.30 | -1.92 | -2.11 | 0.27 |
| 329 | 9080 | CLDN9 | claudin 9 | -2.50 | -1.72 | -2.11 | 0.56 |
| 330 | 6446 | SGK1 | serum/glucocorticoid regulated kinase 1 | -2.10 | -2.12 | -2.11 | 0.01 |
| 331 | 7127 | TNFAIP2 | TNF alpha induced protein 2 | -2.17 | -2.02 | -2.09 | 0.11 |
| 332 | 54937 | SOHLH2 | spermatogenesis and oogenesis specific basic helix-loop-helix 2 | -2.18 | -2.01 | -2.09 | 0.12 |
| 333 | 3993 | LLGL2 | LLGL2. scribble cell polarity complex component | -2.53 | -1.65 | -2.09 | 0.63 |
| 334 | 5777 | PTPN6 | protein tyrosine phosphatase. non-receptor type 6 | -1.98 | -2.18 | -2.08 | 0.14 |
| 335 | 27293 | SMPDL3B | sphingomyelin phosphodiesterase acid like 3B | -1.91 | -2.25 | -2.08 | 0.24 |
| 336 | 10317 | B3GALT5 | beta-1.3-galactosyltransferase 5 | -1.65 | -2.49 | -2.07 | 0.60 |
| 337 | 10217 | CTDSPL | CTD small phosphatase like | -2.17 | -1.97 | -2.07 | 0.14 |
| 338 | 392636 | AGMO | alkylglycerol monooxygenase | -2.56 | -1.57 | -2.07 | 0.70 |
| 339 | 256710 | GLIPR1L1 | GLI pathogenesis related 1 like 1 | -2.46 | -1.67 | -2.07 | 0.56 |
| 340 | 79412 | KREMEN2 | kringle containing transmembrane protein 2 | -2.02 | -2.11 | -2.06 | 0.07 |
| 341 | 148645 | LINC00337 | long intergenic non-protein coding RNA 337 | -1.95 | -2.17 | -2.06 | 0.15 |
| 342 | 26996 | GPR160 | G protein-coupled receptor 160 | -1.63 | -2.47 | -2.05 | 0.59 |
| 343 | 392275 | SMPD5 | sphingomyelin phosphodiesterase 5 | -2.23 | -1.87 | -2.05 | 0.25 |
| 344 | 9940 | DLEC1 | deleted in lung and esophageal cancer 1 | -2.26 | -1.83 | -2.04 | 0.30 |
| 345 | 54502 | RBM47 | RNA binding motif protein 47 | -1.86 | -2.23 | -2.04 | 0.26 |
| 346 | 150368 | PHETA2 | family with sequence similarity 109 member B | -1.90 | -2.18 | -2.04 | 0.19 |
| 347 | 57228 | SMAGP | small cell adhesion glycoprotein | -2.28 | -1.80 | -2.04 | 0.34 |
| 348 | 8828 | NRP2 | neuropilin 2 | -2.21 | -1.86 | -2.04 | 0.25 |
| 349 | 6329 | SCN4A | sodium voltage-gated channel alpha subunit 4 | -2.87 | -1.17 | -2.02 | 1.20 |
| 350 | 80704 | SLC19A3 | solute carrier family 19 member 3 | -2.19 | -1.86 | -2.02 | 0.23 |
| 351 | 729830 | FAM160A1 | family with sequence similarity 160 member A1 | -1.79 | -2.25 | -2.02 | 0.32 |
| 352 | 100506966 | N/A | N/A | -1.91 | -2.12 | -2.02 | 0.15 |
| 353 | 100132891 | MSC-AS1 | MSC antisense RNA 1 | -2.02 | -2.01 | -2.01 | 0.00 |
| 354 | 9945 | GFPT2 | glutamine-fructose-6-phosphate transaminase 2 | -2.31 | -1.72 | -2.01 | 0.42 |
| 355 | 4825 | NKX6-1 | NK6 homeobox 1 | -2.22 | -1.80 | -2.01 | 0.30 |
| 356 | 117283 | IP6K3 | inositol hexakisphosphate kinase 3 | -2.43 | -1.58 | -2.00 | 0.60 |
| 357 | 2810 | SFN | stratifin | -2.36 | -1.64 | -2.00 | 0.51 |
| 358 | 145624 | PWAR1 | Prader Willi/Angelman region RNA 1 | -2.21 | -1.79 | -2.00 | 0.30 |
| 359 | 29943 | PADI1 | peptidyl arginine deiminase 1 | -2.17 | -1.83 | -2.00 | 0.24 |
| 360 | 27164 | SALL3 | spalt like transcription factor 3 | -1.82 | -2.14 | -1.98 | 0.23 |
| 361 | 5336 | PLCG2 | phospholipase C gamma 2 | -2.58 | -1.38 | -1.98 | 0.85 |
| 362 | 6337 | SCNN1A | sodium channel epithelial 1 alpha subunit | -1.65 | -2.30 | -1.98 | 0.46 |
| 363 | 254295 | PHYHD1 | phytanoyl-CoA dioxygenase domain containing 1 | -2.47 | -1.49 | -1.98 | 0.69 |
| 364 | 375616 | KCP | kielin/chordin-like protein | -2.77 | -1.17 | -1.97 | 1.13 |
| 365 | 9965 | FGF19 | fibroblast growth factor 19 | -2.07 | -1.84 | -1.96 | 0.16 |
| 366 | 56105 | PCDHGA11 | protocadherin gamma subfamily A. 11 | -2.42 | -1.49 | -1.96 | 0.65 |
| 367 | 222537 | HS3ST5 | heparan sulfate-glucosamine 3-sulfotransferase 5 | -1.77 | -2.13 | -1.95 | 0.25 |
| 368 | 84504 | NKX6-2 | NK6 homeobox 2 | -1.95 | -1.94 | -1.95 | 0.00 |
| 369 | 56243 | KIAA1217 | KIAA1217 | -2.03 | -1.87 | -1.95 | 0.11 |
| 370 | 11030 | RBPMS | RNA binding protein with multiple splicing | -2.25 | -1.64 | -1.94 | 0.44 |
| 371 | 200407 | CREG2 | cellular repressor of E1A stimulated genes 2 | -2.29 | -1.60 | -1.94 | 0.49 |
| 372 | 5920 | PLAAT4 | retinoic acid receptor responder 3 | -2.10 | -1.78 | -1.94 | 0.22 |
| 373 | 9071 | CLDN10 | claudin 10 | -2.57 | -1.31 | -1.94 | 0.89 |
| 374 | 10886 | NPFFR2 | neuropeptide FF receptor 2 | -1.99 | -1.89 | -1.94 | 0.07 |
| 375 | 641700 | ECSCR | endothelial cell surface expressed chemotaxis and apoptosis regulator | -1.96 | -1.92 | -1.94 | 0.03 |
| 376 | 634 | CEACAM1 | carcinoembryonic antigen related cell adhesion molecule 1 | -2.02 | -1.84 | -1.93 | 0.13 |
| 377 | 154790 | CLEC2L | C-type lectin domain family 2 member L | -1.77 | -2.08 | -1.92 | 0.22 |
| 378 | 6236 | RRAD | RRAD. Ras related glycolysis inhibitor and calcium channel regulator | -1.72 | -2.12 | -1.92 | 0.29 |
| 379 | 6813 | STXBP2 | syntaxin binding protein 2 | -1.78 | -2.05 | -1.92 | 0.19 |
| 380 | 64114 | TMBIM1 | transmembrane BAX inhibitor motif containing 1 | -1.81 | -2.02 | -1.91 | 0.14 |
| 381 | 401 | PHOX2A | paired like homeobox 2a | -1.62 | -2.21 | -1.91 | 0.42 |
| 382 | 100506351 | N/A | N/A | -1.67 | -2.15 | -1.91 | 0.35 |
| 383 | 79651 | RHBDF2 | rhomboid 5 homolog 2 | -2.02 | -1.78 | -1.90 | 0.16 |
| 384 | 56475 | RPRM | reprimo. TP53 dependent G2 arrest mediator candidate | -1.90 | -1.89 | -1.89 | 0.01 |
| 385 | 125965 | COX6B2 | cytochrome c oxidase subunit 6B2 | -1.89 | -1.89 | -1.89 | 0.00 |
| 386 | 100507177 | N/A | N/A | -1.91 | -1.86 | -1.89 | 0.04 |
| 387 | 64116 | SLC39A8 | solute carrier family 39 member 8 | -2.08 | -1.69 | -1.88 | 0.28 |
| 388 | 5167 | ENPP1 | ectonucleotide pyrophosphatase/phosphodiesterase 1 | -1.81 | -1.94 | -1.88 | 0.09 |
| 389 | 64798 | DEPTOR | DEP domain containing MTOR-interacting protein | -1.64 | -2.11 | -1.87 | 0.34 |
| 390 | 10346 | TRIM22 | tripartite motif containing 22 | -2.29 | -1.45 | -1.87 | 0.59 |
| 391 | 162461 | TMEM92 | transmembrane protein 92 | -1.95 | -1.78 | -1.86 | 0.12 |
| 392 | 100130238 | LOC100130238 | uncharacterized LOC100130238 | -2.14 | -1.57 | -1.86 | 0.41 |
| 393 | 162966 | ZNF600 | zinc finger protein 600 | -1.70 | -2.01 | -1.85 | 0.22 |
| 394 | 84958 | SYTL1 | synaptotagmin like 1 | -2.30 | -1.40 | -1.85 | 0.64 |
| 395 | 5625 | PRODH | proline dehydrogenase 1 | -1.95 | -1.75 | -1.85 | 0.14 |
| 396 | 4647 | MYO7A | myosin VIIA | -2.16 | -1.53 | -1.85 | 0.45 |
| 397 | 10622 | POLR3G | RNA polymerase III subunit G | -1.80 | -1.88 | -1.84 | 0.05 |
| 398 | 255738 | PCSK9 | proprotein convertase subtilisin/kexin type 9 | -1.59 | -2.09 | -1.84 | 0.35 |
| 399 | 56704 | JPH1 | junctophilin 1 | -2.11 | -1.57 | -1.84 | 0.38 |
| 400 | 3386 | ICAM4 | intercellular adhesion molecule 4 (Landsteiner-Wiener blood group) | -1.64 | -2.03 | -1.83 | 0.28 |
| 401 | 285965 | EPHA1-AS1 | EPHA1 antisense RNA 1 | -1.75 | -1.90 | -1.82 | 0.11 |
| 402 | 80059 | LRRTM4 | leucine rich repeat transmembrane neuronal 4 | -1.90 | -1.75 | -1.82 | 0.10 |
| 403 | 5652 | PRSS8 | protease. serine 8 | -1.87 | -1.78 | -1.82 | 0.06 |
| 404 | 316 | AOX1 | aldehyde oxidase 1 | -2.20 | -1.44 | -1.82 | 0.54 |
| 405 | 1356 | CP | ceruloplasmin | -1.05 | -2.59 | -1.82 | 1.08 |
| 406 | 974 | CD79B | CD79b molecule | -1.93 | -1.71 | -1.82 | 0.16 |
| 407 | 53827 | FXYD5 | FXYD domain containing ion transport regulator 5 | -1.91 | -1.71 | -1.81 | 0.14 |
| 408 | 6819 | SULT1C2 | sulfotransferase family 1C member 2 | -2.27 | -1.34 | -1.81 | 0.65 |
| 409 | 126433 | FBXO27 | F-box protein 27 | -2.26 | -1.35 | -1.80 | 0.65 |
| 410 | 644815 | FAM83G | family with sequence similarity 83 member G | -2.01 | -1.60 | -1.80 | 0.29 |
| 411 | 79838 | TMC5 | transmembrane channel like 5 | -2.32 | -1.28 | -1.80 | 0.73 |
| 412 | 9848 | MFAP3L | microfibrillar associated protein 3 like | -2.00 | -1.59 | -1.80 | 0.29 |
| 413 | 50848 | F11R | F11 receptor | -1.66 | -1.92 | -1.79 | 0.18 |
| 414 | 3635 | INPP5D | inositol polyphosphate-5-phosphatase D | -1.55 | -2.03 | -1.79 | 0.34 |
| 415 | 51450 | PRRX2 | paired related homeobox 2 | -1.85 | -1.73 | -1.79 | 0.09 |
| 416 | 646643 | SBK2 | SH3 domain binding kinase family member 2 | -1.94 | -1.64 | -1.79 | 0.21 |
| 417 | 221806 | VWDE | von Willebrand factor D and EGF domains | -1.82 | -1.76 | -1.79 | 0.04 |
| 418 | 2877 | GPX2 | glutathione peroxidase 2 | -1.98 | -1.59 | -1.79 | 0.28 |
| 419 | 91523 | PCED1B | PC-esterase domain containing 1B | -1.56 | -2.01 | -1.78 | 0.32 |
| 420 | 5376 | PMP22 | peripheral myelin protein 22 | -1.75 | -1.81 | -1.78 | 0.04 |
| 421 | 5097 | PCDH1 | protocadherin 1 | -1.68 | -1.86 | -1.77 | 0.12 |
| 422 | 728755 | LOC728755 | uncharacterized LOC728755 | -1.95 | -1.59 | -1.77 | 0.26 |
| 423 | 285359 | PDCL3P4 | phosducin-like 3 pseudogene 4 | -1.40 | -2.13 | -1.77 | 0.52 |
| 424 | 6693 | SPN | sialophorin | -1.81 | -1.71 | -1.76 | 0.07 |
| 425 | 954 | ENTPD2 | ectonucleoside triphosphate diphosphohydrolase 2 | -2.14 | -1.37 | -1.75 | 0.54 |
| 426 | 3791 | KDR | kinase insert domain receptor | -2.38 | -1.12 | -1.75 | 0.90 |
| 427 | 9723 | SEMA3E | semaphorin 3E | -2.24 | -1.25 | -1.74 | 0.71 |
| 428 | 1308 | COL17A1 | collagen type XVII alpha 1 chain | -1.99 | -1.49 | -1.74 | 0.36 |
| 429 | 146802 | SLC47A2 | solute carrier family 47 member 2 | -2.28 | -1.19 | -1.73 | 0.77 |
| 430 | 400685 | LINC01801 | uncharacterized LOC400685 | -2.27 | -1.19 | -1.73 | 0.76 |
| 431 | 100129196 | MATN1-AS1 | MATN1 antisense RNA 1 | -1.87 | -1.59 | -1.73 | 0.20 |
| 432 | 283089 | WDR11-AS1 | WDR11 antisense RNA 1 | -1.83 | -1.63 | -1.73 | 0.14 |
| 433 | 26030 | PLEKHG3 | pleckstrin homology and RhoGEF domain containing G3 | -2.01 | -1.43 | -1.72 | 0.41 |
| 434 | 3274 | HRH2 | histamine receptor H2 | -2.26 | -1.18 | -1.72 | 0.76 |
| 435 | 9478 | CABP1 | calcium binding protein 1 | -1.80 | -1.63 | -1.71 | 0.12 |
| 436 | 928 | CD9 | CD9 molecule | -1.33 | -2.10 | -1.71 | 0.55 |
| 437 | 404550 | C16orf74 | chromosome 16 open reading frame 74 | -1.44 | -1.97 | -1.71 | 0.38 |
| 438 | 4838 | NODAL | nodal growth differentiation factor | -1.45 | -1.94 | -1.70 | 0.34 |
| 439 | 79933 | SYNPO2L | synaptopodin 2 like | -2.10 | -1.29 | -1.69 | 0.57 |
| 440 | 55268 | ECHDC2 | enoyl-CoA hydratase domain containing 2 | -1.81 | -1.57 | -1.69 | 0.17 |
| 441 | 342527 | SMTNL2 | smoothelin like 2 | -1.33 | -2.05 | -1.69 | 0.50 |
| 442 | 162979 | ZNF296 | zinc finger protein 296 | -1.70 | -1.67 | -1.69 | 0.03 |
| 443 | 2649 | NR6A1 | nuclear receptor subfamily 6 group A member 1 | -1.81 | -1.55 | -1.68 | 0.19 |
| 444 | 23401 | FRAT2 | frequently rearranged in advanced T-cell lymphomas 2 | -1.55 | -1.81 | -1.68 | 0.18 |
| 445 | 64856 | VWA1 | von Willebrand factor A domain containing 1 | -1.59 | -1.76 | -1.68 | 0.13 |
| 446 | 7058 | THBS2 | thrombospondin 2 | -1.76 | -1.59 | -1.67 | 0.13 |
| 447 | 340075 | ARSI | arylsulfatase family member I | -1.83 | -1.52 | -1.67 | 0.22 |
| 448 | 23237 | ARC | activity regulated cytoskeleton associated protein | -1.44 | -1.91 | -1.67 | 0.34 |
| 449 | 9242 | MSC | musculin | -1.58 | -1.77 | -1.67 | 0.13 |
| 450 | 57552 | NCEH1 | neutral cholesterol ester hydrolase 1 | -1.38 | -1.95 | -1.67 | 0.40 |
| 451 | 132112 | RTP1 | receptor transporter protein 1 | -1.74 | -1.58 | -1.66 | 0.11 |
| 452 | 64065 | PERP | PERP. TP53 apoptosis effector | -2.16 | -1.16 | -1.66 | 0.71 |
| 453 | 306 | ANXA3 | annexin A3 | -2.05 | -1.26 | -1.66 | 0.56 |
| 454 | 10610 | ST6GALNAC2 | ST6 N-acetylgalactosaminide alpha-2.6-sialyltransferase 2 | -1.83 | -1.48 | -1.65 | 0.25 |
| 455 | 6515 | SLC2A3 | solute carrier family 2 member 3 | -1.76 | -1.54 | -1.65 | 0.15 |
| 456 | 1264 | CNN1 | calponin 1 | -2.27 | -1.02 | -1.64 | 0.88 |
| 457 | 9435 | CHST2 | carbohydrate sulfotransferase 2 | -1.71 | -1.58 | -1.64 | 0.09 |
| 458 | 64787 | EPS8L2 | EPS8 like 2 | -1.82 | -1.47 | -1.64 | 0.25 |
| 459 | 389102 | N/A | N/A | -1.81 | -1.47 | -1.64 | 0.24 |
| 460 | 5028 | P2RY1 | purinergic receptor P2Y1 | -1.65 | -1.63 | -1.64 | 0.02 |
| 461 | 2242 | FES | FES proto-oncogene. tyrosine kinase | -1.52 | -1.74 | -1.63 | 0.16 |
| 462 | 92211 | CDHR1 | cadherin related family member 1 | -2.05 | -1.21 | -1.63 | 0.60 |
| 463 | 27106 | ARRDC2 | arrestin domain containing 2 | -1.36 | -1.90 | -1.63 | 0.38 |
| 464 | 91584 | PLXNA4 | plexin A4 | -1.65 | -1.61 | -1.63 | 0.03 |
| 465 | 10420 | TESK2 | testis-specific kinase 2 | -1.55 | -1.71 | -1.63 | 0.12 |
| 466 | 131405 | TRIM71 | tripartite motif containing 71 | -1.79 | -1.46 | -1.63 | 0.23 |
| 467 | 6095 | RORA | RAR related orphan receptor A | -1.28 | -1.98 | -1.63 | 0.49 |
| 468 | 6258 | RXRG | retinoid X receptor gamma | -1.75 | -1.49 | -1.62 | 0.18 |
| 469 | 54438 | GFOD1 | glucose-fructose oxidoreductase domain containing 1 | -1.69 | -1.56 | -1.62 | 0.09 |
| 470 | 1789 | DNMT3B | DNA methyltransferase 3 beta | -1.65 | -1.58 | -1.62 | 0.05 |
| 471 | 83959 | SLC4A11 | solute carrier family 4 member 11 | -1.52 | -1.71 | -1.62 | 0.14 |
| 472 | 146206 | CARMIL2 | capping protein regulator and myosin 1 linker 2 | -2.00 | -1.23 | -1.61 | 0.55 |
| 473 | 56154 | TEX15 | testis expressed 15 | -2.13 | -1.07 | -1.60 | 0.75 |
| 474 | 169200 | TMEM64 | transmembrane protein 64 | -1.24 | -1.95 | -1.60 | 0.50 |
| 475 | 8309 | ACOX2 | acyl-CoA oxidase 2 | -1.97 | -1.23 | -1.60 | 0.52 |
| 476 | 55966 | AJAP1 | adherens junctions associated protein 1 | -1.47 | -1.71 | -1.59 | 0.17 |
| 477 | 100134229 | KDM7A-DT | JHDM1D antisense RNA 1 (head to head) | -1.67 | -1.49 | -1.58 | 0.13 |
| 478 | 307 | ANXA4 | annexin A4 | -1.61 | -1.55 | -1.58 | 0.04 |
| 479 | 5630 | PRPH | peripherin | -1.31 | -1.84 | -1.58 | 0.37 |
| 480 | 6769 | STAC | SH3 and cysteine rich domain | -1.73 | -1.42 | -1.57 | 0.22 |
| 481 | 2046 | EPHA8 | EPH receptor A8 | -1.84 | -1.30 | -1.57 | 0.38 |
| 482 | 79624 | ARMT1 | acidic residue methyltransferase 1 | -1.36 | -1.78 | -1.57 | 0.30 |
| 483 | 4688 | NCF2 | neutrophil cytosolic factor 2 | -1.54 | -1.60 | -1.57 | 0.04 |
| 484 | 141 | ADPRH | ADP-ribosylarginine hydrolase | -1.65 | -1.48 | -1.56 | 0.12 |
| 485 | 63898 | SH2D4A | SH2 domain containing 4A | -1.55 | -1.57 | -1.56 | 0.02 |
| 486 | 85442 | KNDC1 | kinase non-catalytic C-lobe domain containing 1 | -1.88 | -1.24 | -1.56 | 0.45 |
| 487 | 23428 | SLC7A8 | solute carrier family 7 member 8 | -1.79 | -1.32 | -1.56 | 0.34 |
| 488 | 79805 | VASH2 | vasohibin 2 | -1.15 | -1.96 | -1.55 | 0.57 |
| 489 | 10468 | FST | follistatin | -1.85 | -1.26 | -1.55 | 0.41 |
| 490 | 117247 | SLC16A10 | solute carrier family 16 member 10 | -1.21 | -1.88 | -1.55 | 0.48 |
| 491 | 79887 | PLBD1 | phospholipase B domain containing 1 | -1.38 | -1.71 | -1.54 | 0.23 |
| 492 | 100033416 | SNORD116-4 | small nucleolar RNA. C/D box 116-4 | -2.05 | -1.03 | -1.54 | 0.73 |
| 493 | 125206 | SLC5A10 | solute carrier family 5 member 10 | -1.90 | -1.18 | -1.54 | 0.51 |
| 494 | 55196 | RESF1 | KIAA1551 | -1.35 | -1.72 | -1.53 | 0.26 |
| 495 | 5318 | PKP2 | plakophilin 2 | -2.01 | -1.05 | -1.53 | 0.68 |
| 496 | 54101 | RIPK4 | receptor interacting serine/threonine kinase 4 | -1.50 | -1.55 | -1.53 | 0.04 |
| 497 | 3595 | IL12RB2 | interleukin 12 receptor subunit beta 2 | -1.50 | -1.55 | -1.52 | 0.03 |
| 498 | 6274 | S100A3 | S100 calcium binding protein A3 | -1.85 | -1.19 | -1.52 | 0.47 |
| 499 | 4922 | NTS | neurotensin | -1.61 | -1.41 | -1.51 | 0.15 |
| 500 | 56999 | ADAMTS9 | ADAM metallopeptidase with thrombospondin type 1 motif 9 | -1.73 | -1.28 | -1.51 | 0.32 |
| 501 | 7164 | TPD52L1 | tumor protein D52-like 1 | -1.45 | -1.56 | -1.51 | 0.08 |
| 502 | 152404 | IGSF11 | immunoglobulin superfamily member 11 | -1.78 | -1.22 | -1.50 | 0.40 |
| 503 | 220108 | FAM124A | family with sequence similarity 124 member A | -1.66 | -1.33 | -1.49 | 0.23 |
| 504 | 7035 | TFPI | tissue factor pathway inhibitor | -1.78 | -1.21 | -1.49 | 0.40 |
| 505 | 55773 | TBC1D23 | TBC1 domain family member 23 | -1.16 | -1.81 | -1.49 | 0.46 |
| 506 | 100128242 | N/A | uncharacterized LOC100128242 | -1.96 | -1.01 | -1.48 | 0.67 |
| 507 | 3589 | IL11 | interleukin 11 | -1.25 | -1.72 | -1.48 | 0.33 |
| 508 | 9056 | SLC7A7 | solute carrier family 7 member 7 | -1.60 | -1.36 | -1.48 | 0.17 |
| 509 | 10736 | SIX2 | SIX homeobox 2 | -1.23 | -1.72 | -1.48 | 0.35 |
| 510 | 8291 | DYSF | dysferlin | -1.12 | -1.83 | -1.47 | 0.50 |
| 511 | 3428 | IFI16 | interferon gamma inducible protein 16 | -1.31 | -1.61 | -1.46 | 0.21 |
| 512 | 7360 | UGP2 | UDP-glucose pyrophosphorylase 2 | -1.22 | -1.71 | -1.46 | 0.35 |
| 513 | 3880 | KRT19 | keratin 19 | -1.29 | -1.64 | -1.46 | 0.25 |
| 514 | 55113 | XKR8 | XK related 8 | -1.70 | -1.20 | -1.45 | 0.36 |
| 515 | 6281 | S100A10 | S100 calcium binding protein A10 | -1.74 | -1.16 | -1.45 | 0.41 |
| 516 | 57194 | ATP10A | ATPase phospholipid transporting 10A (putative) | -1.69 | -1.21 | -1.45 | 0.34 |
| 517 | 6776 | STAT5A | signal transducer and activator of transcription 5A | -1.09 | -1.80 | -1.45 | 0.50 |
| 518 | 144347 | RFLNA | refilin A | -1.35 | -1.54 | -1.44 | 0.14 |
| 519 | 80054 | CEBPA-DT | CEBPA antisense RNA 1 (head to head) | -1.75 | -1.14 | -1.44 | 0.43 |
| 520 | 51127 | TRIM17 | tripartite motif containing 17 | -1.33 | -1.55 | -1.44 | 0.15 |
| 521 | 90649 | ZNF486 | zinc finger protein 486 | -1.29 | -1.59 | -1.44 | 0.21 |
| 522 | 400954 | EML6 | echinoderm microtubule associated protein like 6 | -1.56 | -1.30 | -1.43 | 0.19 |
| 523 | 643965 | TMEM88B | transmembrane protein 88B | -1.57 | -1.29 | -1.43 | 0.19 |
| 524 | 55766 | H2AJ | H2A histone family member J | -1.36 | -1.49 | -1.43 | 0.10 |
| 525 | 133522 | PPARGC1B | PPARG coactivator 1 beta | -1.44 | -1.41 | -1.42 | 0.02 |
| 526 | 132014 | IL17RE | interleukin 17 receptor E | -1.23 | -1.62 | -1.42 | 0.27 |
| 527 | 10553 | HTATIP2 | HIV-1 Tat interactive protein 2 | -1.23 | -1.60 | -1.42 | 0.26 |
| 528 | 677809 | SNORA24 | small nucleolar RNA. H/ACA box 24 | -1.65 | -1.19 | -1.42 | 0.33 |
| 529 | 80832 | APOL4 | apolipoprotein L4 | -1.20 | -1.63 | -1.41 | 0.30 |
| 530 | 27241 | BBS9 | Bardet-Biedl syndrome 9 | -1.48 | -1.35 | -1.41 | 0.09 |
| 531 | 341640 | FREM2 | FRAS1 related extracellular matrix protein 2 | -1.69 | -1.12 | -1.41 | 0.40 |
| 532 | 5493 | PPL | periplakin | -1.68 | -1.13 | -1.40 | 0.39 |
| 533 | 57556 | SEMA6A | semaphorin 6A | -1.29 | -1.51 | -1.40 | 0.16 |
| 534 | 7444 | VRK2 | vaccinia related kinase 2 | -1.72 | -1.08 | -1.40 | 0.45 |
| 535 | 128344 | PIFO | primary cilia formation | -1.35 | -1.45 | -1.40 | 0.07 |
| 536 | 64218 | SEMA4A | semaphorin 4A | -1.35 | -1.44 | -1.39 | 0.06 |
| 537 | 9780 | PIEZO1 | piezo type mechanosensitive ion channel component 1 | -1.50 | -1.28 | -1.39 | 0.15 |
| 538 | 22949 | PTGR1 | prostaglandin reductase 1 | -1.43 | -1.35 | -1.39 | 0.05 |
| 539 | 9510 | ADAMTS1 | ADAM metallopeptidase with thrombospondin type 1 motif 1 | -1.65 | -1.12 | -1.39 | 0.38 |
| 540 | 132671 | SPATA18 | spermatogenesis associated 18 | -1.33 | -1.45 | -1.39 | 0.08 |
| 541 | 1006 | CDH8 | cadherin 8 | -1.33 | -1.45 | -1.39 | 0.09 |
| 542 | 1050 | CEBPA | CCAAT/enhancer binding protein alpha | -1.34 | -1.41 | -1.38 | 0.05 |
| 543 | 6649 | SOD3 | superoxide dismutase 3. extracellular | -1.63 | -1.12 | -1.37 | 0.36 |
| 544 | 54532 | USP53 | ubiquitin specific peptidase 53 | -1.25 | -1.49 | -1.37 | 0.17 |
| 545 | 92840 | REEP6 | receptor accessory protein 6 | -1.36 | -1.38 | -1.37 | 0.01 |
| 546 | 6663 | SOX10 | SRY-box 10 | -1.68 | -1.06 | -1.37 | 0.44 |
| 547 | 2651 | GCNT2 | glucosaminyl (N-acetyl) transferase 2. I-branching enzyme (I blood group) | -1.40 | -1.31 | -1.35 | 0.06 |
| 548 | 6480 | ST6GAL1 | ST6 beta-galactoside alpha-2.6-sialyltransferase 1 | -1.27 | -1.43 | -1.35 | 0.11 |
| 549 | 1373 | CPS1 | carbamoyl-phosphate synthase 1 | -1.59 | -1.10 | -1.34 | 0.35 |
| 550 | 54863 | TOR4A | torsin family 4 member A | -1.30 | -1.38 | -1.34 | 0.06 |
| 551 | 4254 | KITLG | KIT ligand | -1.22 | -1.45 | -1.34 | 0.16 |
| 552 | 8459 | TPST2 | tyrosylprotein sulfotransferase 2 | -1.38 | -1.28 | -1.33 | 0.07 |
| 553 | 80144 | FRAS1 | Fraser extracellular matrix complex subunit 1 | -1.40 | -1.24 | -1.32 | 0.11 |
| 554 | 83850 | ESYT3 | extended synaptotagmin 3 | -1.23 | -1.39 | -1.31 | 0.11 |
| 555 | 84502 | JPH4 | junctophilin 4 | -1.50 | -1.12 | -1.31 | 0.27 |
| 556 | 822 | CAPG | capping actin protein. gelsolin like | -1.18 | -1.42 | -1.30 | 0.17 |
| 557 | 66000 | TMEM108 | transmembrane protein 108 | -1.27 | -1.33 | -1.30 | 0.05 |
| 558 | 7453 | WARS1 | tryptophanyl-tRNA synthetase | -1.38 | -1.22 | -1.30 | 0.11 |
| 559 | 4067 | LYN | LYN proto-oncogene. Src family tyrosine kinase | -1.37 | -1.21 | -1.29 | 0.11 |
| 560 | 94059 | LENG9 | leukocyte receptor cluster member 9 | -1.53 | -1.05 | -1.29 | 0.34 |
| 561 | 6620 | SNCB | synuclein beta | -1.51 | -1.08 | -1.29 | 0.31 |
| 562 | 8635 | RNASET2 | ribonuclease T2 | -1.33 | -1.25 | -1.29 | 0.06 |
| 563 | 5971 | RELB | RELB proto-oncogene. NF-kB subunit | -1.50 | -1.05 | -1.28 | 0.32 |
| 564 | 79937 | CNTNAP3 | contactin associated protein-like 3 | -1.31 | -1.24 | -1.27 | 0.04 |
| 565 | 53833 | IL20RB | interleukin 20 receptor subunit beta | -1.31 | -1.23 | -1.27 | 0.05 |
| 566 | 10588 | MTHFS | 5.10-methenyltetrahydrofolate synthetase (5-formyltetrahydrofolate cyclo-ligase) | -1.38 | -1.15 | -1.27 | 0.16 |
| 567 | 55211 | DPPA4 | developmental pluripotency associated 4 | -1.18 | -1.34 | -1.26 | 0.12 |
| 568 | 283345 | RPL13P5 | ribosomal protein L13 pseudogene 5 | -1.19 | -1.32 | -1.26 | 0.09 |
| 569 | 3912 | LAMB1 | laminin subunit beta 1 | -1.21 | -1.27 | -1.24 | 0.04 |
| 570 | 5836 | PYGL | phosphorylase. glycogen. liver | -1.13 | -1.34 | -1.24 | 0.15 |
| 571 | 392255 | GDF6 | growth differentiation factor 6 | -1.45 | -1.02 | -1.23 | 0.31 |
| 572 | 79026 | AHNAK | AHNAK nucleoprotein | -1.47 | -1.00 | -1.23 | 0.33 |
| 573 | 5578 | PRKCA | protein kinase C alpha | -1.45 | -1.01 | -1.23 | 0.31 |
| 574 | 89874 | SLC25A21 | solute carrier family 25 member 21 | -1.44 | -1.01 | -1.23 | 0.30 |
| 575 | 100 | ADA | adenosine deaminase | -1.21 | -1.24 | -1.23 | 0.02 |
| 576 | 55106 | SLFN12 | schlafen family member 12 | -1.29 | -1.16 | -1.22 | 0.09 |
| 577 | 4861 | NPAS1 | neuronal PAS domain protein 1 | -1.33 | -1.11 | -1.22 | 0.16 |
| 578 | 645431 | FUT8-AS1 | FUT8 antisense RNA 1 | -1.23 | -1.19 | -1.21 | 0.03 |
| 579 | 1757 | SARDH | sarcosine dehydrogenase | -1.09 | -1.32 | -1.21 | 0.16 |
| 580 | 3988 | LIPA | lipase A. lysosomal acid type | -1.01 | -1.39 | -1.20 | 0.27 |
| 581 | 2150 | F2RL1 | F2R like trypsin receptor 1 | -1.03 | -1.34 | -1.19 | 0.22 |
| 582 | 51385 | ZNF589 | zinc finger protein 589 | -1.28 | -1.07 | -1.18 | 0.15 |
| 583 | 255928 | SYT14 | synaptotagmin 14 | -1.25 | -1.10 | -1.17 | 0.11 |
| 584 | 3710 | ITPR3 | inositol 1.4.5-trisphosphate receptor type 3 | -1.18 | -1.17 | -1.17 | 0.00 |
| 585 | 3691 | ITGB4 | integrin subunit beta 4 | -1.20 | -1.07 | -1.14 | 0.10 |
| 586 | 10797 | MTHFD2 | methylenetetrahydrofolate dehydrogenase (NADP+ dependent) 2. methenyltetrahydrofolate cyclohydrolase | -1.24 | -1.02 | -1.13 | 0.16 |
| 587 | 23242 | COBL | cordon-bleu WH2 repeat protein | -1.13 | -1.12 | -1.13 | 0.01 |
| 588 | 10678 | B3GNT2 | UDP-GlcNAc:betaGal beta-1.3-N-acetylglucosaminyltransferase 2 | -1.12 | -1.12 | -1.12 | 0.00 |
| 589 | 30812 | SOX8 | SRY-box 8 | -1.01 | -1.23 | -1.12 | 0.16 |
| 590 | 8462 | KLF11 | Kruppel like factor 11 | -1.06 | -1.16 | -1.11 | 0.07 |
| 591 | 23446 | SLC44A1 | solute carrier family 44 member 1 | -1.01 | -1.18 | -1.10 | 0.12 |
| 592 | 1809 | DPYSL3 | dihydropyrimidinase like 3 | -1.08 | -1.08 | -1.08 | 0.00 |
| 593 | 100093630 | SNHG8 | small nucleolar RNA host gene 8 | -1.07 | -1.09 | -1.08 | 0.02 |
| 594 | 1999 | ELF3 | E74 like ETS transcription factor 3 | -1.02 | -1.12 | -1.07 | 0.07 |
| 595 | 7262 | PHLDA2 | pleckstrin homology like domain family A member 2 | -1.07 | -1.05 | -1.06 | 0.01 |
| 596 | 148213 | ZNF681 | zinc finger protein 681 | -1.03 | -1.05 | -1.04 | 0.01 |
| 597 | 25819 | NOCT | nocturnin | -1.00 | -1.03 | -1.02 | 0.02 |
