# Supplementary material for: Investigation of de novo mutations in a schizophrenia case-parent trio by induced pluripotent stem cell-based in vitro disease modeling: convergence of schizophrenia- and autism-related cellular phenotypes
Source: Stem Cell Res Ther. 2020 Nov 27;11:504. doi: 10.1186/s13287-020-01980-5 (PMC7694414; doi:10.1186/s13287-020-01980-5)
Supplement: Supplementary file 8 — Additional file 8: Supplementary Table 3. List of DE genes in NPC-SZ-HU-PROB compared to both NPC-SZ-HU-FA and NPC-SZ-HU-MO that are regulated by KHSRP. [file 13287_2020_1980_MOESM8_ESM.docx]

**Supplementary Table 3. List of DE genes in NPC-SZ-HU-PROB compared to both NPC-SZ-HU-FA and NPC-SZ-HU-MO that are regulated by KHSRP.**

| ENTREZ ID | SYMBOL | NAME |
| --- | --- | --- |
| 108 | ADCY2 | adenylate cyclase 2 |
| 117 | ADCYAP1R1 | ADCYAP receptor type I |
| 141 | ADPRH | ADP-ribosylarginine hydrolase |
| 306 | ANXA3 | annexin A3 |
| 307 | ANXA4 | annexin A4 |
| 308 | ANXA5 | annexin A5 |
| 639 | PRDM1 | PR/SET domain 1 |
| 658 | BMPR1B | bone morphogenetic protein receptor type 1B |
| 718 | C3 | complement C3 |
| 793 | CALB1 | calbindin 1 |
| 928 | CD9 | CD9 molecule |
| 1006 | CDH8 | cadherin 8 |
| 1007 | CDH9 | cadherin 9 |
| 1012 | CDH13 | cadherin 13 |
| 1050 | CEBPA | CCAAT/enhancer binding protein alpha |
| 1131 | CHRM3 | cholinergic receptor muscarinic 3 |
| 1143 | CHRNB4 | cholinergic receptor nicotinic beta 4 subunit |
| 1278 | COL1A2 | collagen type I alpha 2 chain |
| 1303 | COL12A1 | collagen type XII alpha 1 chain |
| 1366 | CLDN7 | claudin 7 |
| 1373 | CPS1 | carbamoyl-phosphate synthase 1 |
| 1515 | CTSV | cathepsin V |
| 1602 | DACH1 | dachshund family transcription factor 1 |
| 1604 | CD55 | CD55 molecule (Cromer blood group) |
| 1804 | DPP6 | dipeptidyl peptidase like 6 |
| 1809 | DPYSL3 | dihydropyrimidinase like 3 |
| 1814 | DRD3 | dopamine receptor D3 |
| 1832 | DSP | desmoplakin |
| 1870 | E2F2 | E2F transcription factor 2 |
| 1993 | ELAVL2 | ELAV like RNA binding protein 2 |
| 2020 | EN2 | engrailed homeobox 2 |
| 2066 | ERBB4 | erb-b2 receptor tyrosine kinase 4 |
| 2150 | F2RL1 | F2R like trypsin receptor 1 |
| 2152 | F3 | coagulation factor III, tissue factor |
| 2191 | FAP | fibroblast activation protein alpha |
| 2295 | FOXF2 | forkhead box F2 |
| 2335 | FN1 | fibronectin 1 |
| 2562 | GABRB3 | gamma-aminobutyric acid type A receptor beta3 subunit |
| 2619 | GAS1 | growth arrest specific 1 |
| 2650 | GCNT1 | glucosaminyl (N-acetyl) transferase 1, core 2 |
| 2651 | GCNT2 | glucosaminyl (N-acetyl) transferase 2, I-branching enzyme (I blood group) |
| 2674 | GFRA1 | GDNF family receptor alpha 1 |
| 2681 | GGTA1P | glycoprotein, alpha-galactosyltransferase 1 pseudogene |
| 2741 | GLRA1 | glycine receptor alpha 1 |
| 2895 | GRID2 | glutamate ionotropic receptor delta type subunit 2 |
| 2903 | GRIN2A | glutamate ionotropic receptor NMDA type subunit 2A |
| 2911 | GRM1 | glutamate metabotropic receptor 1 |
| 2912 | GRM2 | glutamate metabotropic receptor 2 |
| 2919 | CXCL1 | C-X-C motif chemokine ligand 1 |
| 2982 | GUCY1A3 | guanylate cyclase 1 soluble subunit alpha |
| 3171 | FOXA3 | forkhead box A3 |
| 3248 | HPGD | hydroxyprostaglandin dehydrogenase 15-(NAD) |
| 3269 | HRH1 | histamine receptor H1 |
| 3352 | HTR1D | 5-hydroxytryptamine receptor 1D |
| 3375 | IAPP | islet amyloid polypeptide |
| 3428 | IFI16 | interferon gamma inducible protein 16 |
| 3433 | IFIT2 | interferon induced protein with tetratricopeptide repeats 2 |
| 3635 | INPP5D | inositol polyphosphate-5-phosphatase D |
| 3673 | ITGA2 | integrin subunit alpha 2 |
| 3684 | ITGAM | integrin subunit alpha M |
| 3710 | ITPR3 | inositol 1,4,5-trisphosphate receptor type 3 |
| 3763 | KCNJ6 | potassium voltage-gated channel subfamily J member 6 |
| 3776 | KCNK2 | potassium two pore domain channel subfamily K member 2 |
| 3815 | KIT | KIT proto-oncogene receptor tyrosine kinase |
| 3988 | LIPA | lipase A, lysosomal acid type |
| 4067 | LYN | LYN proto-oncogene, Src family tyrosine kinase |
| 4233 | MET | MET proto-oncogene, receptor tyrosine kinase |
| 4254 | KITLG | KIT ligand |
| 4306 | NR3C2 | nuclear receptor subfamily 3 group C member 2 |
| 4645 | MYO5B | myosin VB |
| 4774 | NFIA | nuclear factor I A |
| 4922 | NTS | neurotensin |
| 4969 | OGN | osteoglycin |
| 5028 | P2RY1 | purinergic receptor P2Y1 |
| 5077 | PAX3 | paired box 3 |
| 5100 | PCDH8 | protocadherin 8 |
| 5153 | PDE1B | phosphodiesterase 1B |
| 5167 | ENPP1 | ectonucleotide pyrophosphatase/phosphodiesterase 1 |
| 5178 | PEG3 | paternally expressed 3 |
| 5308 | PITX2 | paired like homeodomain 2 |
| 5318 | PKP2 | plakophilin 2 |
| 5336 | PLCG2 | phospholipase C gamma 2 |
| 5362 | PLXNA2 | plexin A2 |
| 5376 | PMP22 | peripheral myelin protein 22 |
| 5396 | PRRX1 | paired related homeobox 1 |
| 5454 | POU3F2 | POU class 3 homeobox 2 |
| 5457 | POU4F1 | POU class 4 homeobox 1 |
| 5458 | POU4F2 | POU class 4 homeobox 2 |
| 5578 | PRKCA | protein kinase C alpha |
| 5588 | PRKCQ | protein kinase C theta |
| 5733 | PTGER3 | prostaglandin E receptor 3 |
| 5865 | RAB3B | RAB3B, member RAS oncogene family |
| 5915 | RARB | retinoic acid receptor beta |
| 5992 | RFX4 | regulatory factor X4 |
| 6095 | RORA | RAR related orphan receptor A |
| 6262 | RYR2 | ryanodine receptor 2 |
| 6263 | RYR3 | ryanodine receptor 3 |
| 6387 | CXCL12 | C-X-C motif chemokine ligand 12 |
| 6424 | SFRP4 | secreted frizzled related protein 4 |
| 6446 | SGK1 | serum/glucocorticoid regulated kinase 1 |
| 6489 | ST8SIA1 | ST8 alpha-N-acetyl-neuraminide alpha-2,8-sialyltransferase 1 |
| 6515 | SLC2A3 | solute carrier family 2 member 3 |
| 6518 | SLC2A5 | solute carrier family 2 member 5 |
| 6581 | SLC22A3 | solute carrier family 22 member 3 |
| 6663 | SOX10 | SRY-box 10 |
| 6690 | SPINK1 | serine peptidase inhibitor, Kazal type 1 |
| 6751 | SSTR1 | somatostatin receptor 1 |
| 6752 | SSTR2 | somatostatin receptor 2 |
| 6769 | STAC | SH3 and cysteine rich domain |
| 6819 | SULT1C2 | sulfotransferase family 1C member 2 |
| 7025 | NR2F1 | nuclear receptor subfamily 2 group F member 1 |
| 7035 | TFPI | tissue factor pathway inhibitor |
| 7092 | TLL1 | tolloid like 1 |
| 7113 | TMPRSS2 | transmembrane protease, serine 2 |
| 7127 | TNFAIP2 | TNF alpha induced protein 2 |
| 7164 | TPD52L1 | tumor protein D52-like 1 |
| 7225 | TRPC6 | transient receptor potential cation channel subfamily C member 6 |
| 7368 | UGT8 | UDP glycosyltransferase 8 |
| 7453 | WARS | tryptophanyl-tRNA synthetase |
| 7478 | WNT8A | Wnt family member 8A |
| 7643 | ZNF90 | zinc finger protein 90 |
| 8092 | ALX1 | ALX homeobox 1 |
| 8313 | AXIN2 | axin 2 |
| 8462 | KLF11 | Kruppel like factor 11 |
| 8507 | ENC1 | ectodermal-neural cortex 1 |
| 8614 | STC2 | stanniocalcin 2 |
| 8635 | RNASET2 | ribonuclease T2 |
| 8792 | TNFRSF11A | TNF receptor superfamily member 11a |
| 8840 | WISP1 | WNT1 inducible signaling pathway protein 1 |
| 8854 | ALDH1A2 | aldehyde dehydrogenase 1 family member A2 |
| 8929 | PHOX2B | paired like homeobox 2b |
| 9124 | PDLIM1 | PDZ and LIM domain 1 |
| 9370 | ADIPOQ | adiponectin, C1Q and collagen domain containing |
| 9413 | FAM189A2 | family with sequence similarity 189 member A2 |
| 9480 | ONECUT2 | one cut homeobox 2 |
| 9510 | ADAMTS1 | ADAM metallopeptidase with thrombospondin type 1 motif 1 |
| 9576 | SPAG6 | sperm associated antigen 6 |
| 9723 | SEMA3E | semaphorin 3E |
| 9848 | MFAP3L | microfibrillar associated protein 3 like |
| 9901 | SRGAP3 | SLIT-ROBO Rho GTPase activating protein 3 |
| 10086 | HHLA1 | HERV-H LTR-associating 1 |
| 10124 | ARL4A | ADP ribosylation factor like GTPase 4A |
| 10217 | CTDSPL | CTD small phosphatase like |
| 10317 | B3GALT5 | beta-1,3-galactosyltransferase 5 |
| 10320 | IKZF1 | IKAROS family zinc finger 1 |
| 10346 | TRIM22 | tripartite motif containing 22 |
| 10351 | ABCA8 | ATP binding cassette subfamily A member 8 |
| 10468 | FST | follistatin |
| 10533 | ATG7 | autophagy related 7 |
| 10622 | POLR3G | RNA polymerase III subunit G |
| 10678 | B3GNT2 | UDP-GlcNAc:betaGal beta-1,3-N-acetylglucosaminyltransferase 2 |
| 10797 | MTHFD2 | methylenetetrahydrofolate dehydrogenase |
| 11030 | RBPMS | RNA binding protein with multiple splicing |
| 11069 | RAPGEF4 | Rap guanine nucleotide exchange factor 4 |
| 11145 | PLA2G16 | phospholipase A2 group XVI |
| 11155 | LDB3 | LIM domain binding 3 |
| 22795 | NID2 | nidogen 2 |
| 22801 | ITGA11 | integrin subunit alpha 11 |
| 22836 | RHOBTB3 | Rho related BTB domain containing 3 |
| 22881 | ANKRD6 | ankyrin repeat domain 6 |
| 22949 | PTGR1 | prostaglandin reductase 1 |
| 23242 | COBL | cordon-bleu WH2 repeat protein |
| 23349 | PHF24 | PHD finger protein 24 |
| 23446 | SLC44A1 | solute carrier family 44 member 1 |
| 23576 | DDAH1 | dimethylarginine dimethylaminohydrolase 1 |
| 25758 | KIAA1549L | KIAA1549 like |
| 25769 | SLC24A2 | solute carrier family 24 member 2 |
| 25825 | BACE2 | beta-site APP-cleaving enzyme 2 |
| 25927 | CNRIP1 | cannabinoid receptor interacting protein 1 |
| 26034 | IPCEF1 | interaction protein for cytohesin exchange factors |
| 26053 | AUTS2 | autism susceptibility candidate 2 |
| 26082 | DKFZP434L187 | uncharacterized LOC26082 |
| 26696 | OR2T1 | olfactory receptor family 2 subfamily T member 1 |
| 27063 | ANKRD1 | ankyrin repeat domain 1 |
| 27145 | FILIP1 | filamin A interacting protein 1 |
| 27164 | SALL3 | spalt like transcription factor 3 |
| 27241 | BBS9 | Bardet-Biedl syndrome 9 |
| 30812 | SOX8 | SRY-box 8 |
| 30816 | ERVW-1 | endogenous retrovirus group W member 1 |
| 51090 | PLLP | plasmolipin |
| 51305 | KCNK9 | potassium two pore domain channel subfamily K member 9 |
| 51384 | WNT16 | Wnt family member 16 |
| 54101 | RIPK4 | receptor interacting serine/threonine kinase 4 |
| 54438 | GFOD1 | glucose-fructose oxidoreductase domain containing 1 |
| 54502 | RBM47 | RNA binding motif protein 47 |
| 54532 | USP53 | ubiquitin specific peptidase 53 |
| 54715 | RBFOX1 | RNA binding protein, fox-1 homolog 1 |
| 54845 | ESRP1 | epithelial splicing regulatory protein 1 |
| 54898 | ELOVL2 | ELOVL fatty acid elongase 2 |
| 54937 | SOHLH2 | spermatogenesis and oogenesis specific basic helix-loop-helix 2 |
| 54941 | RNF125 | ring finger protein 125 |
| 55022 | PID1 | phosphotyrosine interaction domain containing 1 |
| 55106 | SLFN12 | schlafen family member 12 |
| 55211 | DPPA4 | developmental pluripotency associated 4 |
| 55237 | VRTN | vertebrae development associated |
| 55289 | ACOXL | acyl-CoA oxidase-like |
| 55766 | H2AFJ | H2A histone family member J |
| 55773 | TBC1D23 | TBC1 domain family member 23 |
| 55811 | ADCY10 | adenylate cyclase 10, soluble |
| 55966 | AJAP1 | adherens junctions associated protein 1 |
| 56154 | TEX15 | testis expressed 15 |
| 56243 | KIAA1217 | KIAA1217 |
| 56660 | KCNK12 | potassium two pore domain channel subfamily K member 12 |
| 56704 | JPH1 | junctophilin 1 |
| 56978 | PRDM8 | PR/SET domain 8 |
| 56999 | ADAMTS9 | ADAM metallopeptidase with thrombospondin type 1 motif 9 |
| 57125 | PLXDC1 | plexin domain containing 1 |
| 57451 | TENM2 | teneurin transmembrane protein 2 |
| 57471 | ERMN | ermin |
| 57552 | NCEH1 | neutral cholesterol ester hydrolase 1 |
| 57556 | SEMA6A | semaphorin 6A |
| 57604 | KIAA1456 | KIAA1456 |
| 57615 | ZNF492 | zinc finger protein 492 |
| 58158 | NEUROD4 | neuronal differentiation 4 |
| 59335 | PRDM12 | PR/SET domain 12 |
| 63895 | PIEZO2 | piezo type mechanosensitive ion channel component 2 |
| 63901 | FAM111A | family with sequence similarity 111 member A |
| 64065 | PERP | PERP, TP53 apoptosis effector |
| 64116 | SLC39A8 | solute carrier family 39 member 8 |
| 64218 | SEMA4A | semaphorin 4A |
| 64388 | GREM2 | gremlin 2, DAN family BMP antagonist |
| 64478 | CSMD1 | CUB and Sushi multiple domains 1 |
| 64641 | EBF2 | early B-cell factor 2 |
| 66000 | TMEM108 | transmembrane protein 108 |
| 79727 | LIN28A | lin-28 homolog A |
| 79805 | VASH2 | vasohibin 2 |
| 79838 | TMC5 | transmembrane channel like 5 |
| 79923 | NANOG | Nanog homeobox |
| 79937 | CNTNAP3 | contactin associated protein-like 3 |
| 80144 | FRAS1 | Fraser extracellular matrix complex subunit 1 |
| 80704 | SLC19A3 | solute carrier family 19 member 3 |
| 80731 | THSD7B | thrombospondin type 1 domain containing 7B |
| 81285 | OR51E2 | olfactory receptor family 51 subfamily E member 2 |
| 81533 | ITFG1 | integrin alpha FG-GAP repeat containing 1 |
| 81615 | TMEM163 | transmembrane protein 163 |
| 81832 | NETO1 | neuropilin and tolloid like 1 |
| 83445 | GSG1 | germ cell associated 1 |
| 83850 | ESYT3 | extended synaptotagmin 3 |
| 83999 | KREMEN1 | kringle containing transmembrane protein 1 |
| 84002 | B3GNT5 | UDP-GlcNAc:betaGal beta-1,3-N-acetylglucosaminyltransferase 5 |
| 84168 | ANTXR1 | anthrax toxin receptor 1 |
| 84451 | MLK4 | mixed lineage kinase 4 |
| 84457 | PHYHIPL | phytanoyl-CoA 2-hydroxylase interacting protein like |
| 84466 | MEGF10 | multiple EGF like domains 10 |
| 84691 | FAM71F1 | family with sequence similarity 71 member F1 |
| 84803 | GPAT3 | glycerol-3-phosphate acyltransferase 3 |
| 84879 | MFSD2A | major facilitator superfamily domain containing 2A |
| 85407 | NKD1 | naked cuticle homolog 1 |
| 89832 | CHRFAM7A | CHRNA7 |
| 89874 | SLC25A21 | solute carrier family 25 member 21 |
| 90649 | ZNF486 | zinc finger protein 486 |
| 91584 | PLXNA4 | plexin A4 |
| 114795 | TMEM132B | transmembrane protein 132B |
| 115749 | C12orf56 | chromosome 12 open reading frame 56 |
| 116285 | ACSM1 | acyl-CoA synthetase medium-chain family member 1 |
| 117247 | SLC16A10 | solute carrier family 16 member 10 |
| 118788 | PIK3AP1 | phosphoinositide-3-kinase adaptor protein 1 |
| 121601 | ANO4 | anoctamin 4 |
| 124460 | SNX20 | sorting nexin 20 |
| 126669 | SHE | Src homology 2 domain containing E |
| 127833 | SYT2 | synaptotagmin 2 |
| 128344 | PIFO | primary cilia formation |
| 130399 | ACVR1C | activin A receptor type 1C |
| 131096 | KCNH8 | potassium voltage-gated channel subfamily H member 8 |
| 131405 | TRIM71 | tripartite motif containing 71 |
| 132671 | SPATA18 | spermatogenesis associated 18 |
| 133522 | PPARGC1B | PPARG coactivator 1 beta |
| 135398 | C6orf141 | chromosome 6 open reading frame 141 |
| 144195 | SLC2A14 | solute carrier family 2 member 14 |
| 145447 | ABHD12B | abhydrolase domain containing 12B |
| 146857 | SLFN13 | schlafen family member 13 |
| 147495 | APCDD1 | APC down-regulated 1 |
| 148213 | ZNF681 | zinc finger protein 681 |
| 151647 | FAM19A4 | family with sequence similarity 19 member A4, C-C motif chemokine like |
| 151888 | BTLA | B and T lymphocyte associated |
| 152404 | IGSF11 | immunoglobulin superfamily member 11 |
| 153579 | BTNL9 | butyrophilin like 9 |
| 154075 | SAMD3 | sterile alpha motif domain containing 3 |
| 154664 | ABCA13 | ATP binding cassette subfamily A member 13 |
| 157869 | SBSPON | somatomedin B and thrombospondin type 1 domain containing |
| 159963 | SLC5A12 | solute carrier family 5 member 12 |
| 161835 | FSIP1 | fibrous sheath interacting protein 1 |
| 162966 | ZNF600 | zinc finger protein 600 |
| 168667 | BMPER | BMP binding endothelial regulator |
| 169200 | TMEM64 | transmembrane protein 64 |
| 200150 | PLD5 | phospholipase D family member 5 |
| 200407 | CREG2 | cellular repressor of E1A stimulated genes 2 |
| 202374 | STK32A | serine/threonine kinase 32A |
| 202559 | KHDRBS2 | KH RNA binding domain containing, signal transduction associated 2 |
| 220108 | FAM124A | family with sequence similarity 124 member A |
| 221687 | RNF182 | ring finger protein 182 |
| 222171 | PRR15 | proline rich 15 |
| 222537 | HS3ST5 | heparan sulfate-glucosamine 3-sulfotransferase 5 |
| 222584 | FAM83B | family with sequence similarity 83 member B |
| 254295 | PHYHD1 | phytanoyl-CoA dioxygenase domain containing 1 |
| 255738 | PCSK9 | proprotein convertase subtilisin/kexin type 9 |
| 255928 | SYT14 | synaptotagmin 14 |
| 256691 | MAMDC2 | MAM domain containing 2 |
| 256764 | WDR72 | WD repeat domain 72 |
| 256987 | SERINC5 | serine incorporator 5 |
| 259217 | HSPA12A | heat shock protein family A |
| 283089 | WDR11-AS1 | WDR11 antisense RNA 1 |
| 283212 | KLHL35 | kelch like family member 35 |
| 285401 | LINC00698 | long intergenic non-protein coding RNA 698 |
| 285888 | CNPY1 | canopy FGF signaling regulator 1 |
| 285965 | EPHA1-AS1 | EPHA1 antisense RNA 1 |
| 338645 | LUZP2 | leucine zipper protein 2 |
| 339535 | LINC01139 | long intergenic non-protein coding RNA 1139 |
| 341640 | FREM2 | FRAS1 related extracellular matrix protein 2 |
| 344148 | NCKAP5 | NCK associated protein 5 |
| 359787 | DPPA3 | developmental pluripotency associated 3 |
| 374393 | FAM111B | family with sequence similarity 111 member B |
| 388815 | MIR99AHG | mir-99a-let-7c cluster host gene |
| 389206 | BEND4 | BEN domain containing 4 |
| 391712 | TRIM61 | tripartite motif containing 61 |
| 392636 | AGMO | alkylglycerol monooxygenase |
| 400680 | LINC00664 | long intergenic non-protein coding RNA 664 |
| 400954 | EML6 | echinoderm microtubule associated protein like 6 |
| 404744 | NPSR1-AS1 | NPSR1 antisense RNA 1 |
| 440119 | FZD10-AS1 | FZD10 antisense RNA 1 |
| 441307 | HRAT92 | heart tissue-associated transcript 92 |
| 441355 | MIR2052HG | MIR2052 host gene |
| 641364 | SLC7A11-AS1 | SLC7A11 antisense RNA 1 |
| 644150 | WIPF3 | WAS/WASL interacting protein family member 3 |
| 644815 | FAM83G | family with sequence similarity 83 member G |
| 647024 | C6orf132 | chromosome 6 open reading frame 132 |
| 728591 | CCDC169 | coiled-coil domain containing 169 |
| 728780 | ANKDD1B | ankyrin repeat and death domain containing 1B |
| 729085 | FAM198A | family with sequence similarity 198 member A |
| 100132891 | MSC-AS1 | MSC antisense RNA 1 |
| 100132916 | FAM159B | family with sequence similarity 159 member B |
| 100505738 | MIR4458HG | MIR4458 host gene |
| 100506658 | OCLN | occludin |
| 100507173 | LINC01012 | long intergenic non-protein coding RNA 1012 |
