# Supplementary material for: Investigation of de novo mutations in a schizophrenia case-parent trio by induced pluripotent stem cell-based in vitro disease modeling: convergence of schizophrenia- and autism-related cellular phenotypes
Source: Stem Cell Res Ther. 2020 Nov 27;11:504. doi: 10.1186/s13287-020-01980-5 (PMC7694414; doi:10.1186/s13287-020-01980-5)
Supplement: Supplementary file 9 — Additional file 9: Supplementary Table 4. Detailed Statistical analyses. [file 13287_2020_1980_MOESM9_ESM.docx]

**Supplementary Table 4. Detailed Statistical analyses.**

1. **Prolifetarion test, 2-way ANOVA.**

| **Tukey's multiple comparisons test** | **Mean difference** | **95% CI of difference** | **Summary** | **Adjusted p-value** |
| --- | --- | --- | --- | --- |
|  |  |  |  |  |
| **Day 0** |  |  |  |  |
| NPC SZ_HU-FA vs. NPC SZ-HU-MO | 0 | -0.78-0.78 | ns | >0.99 |
| NPC SZ_HU-FA vs. NPC SZ-HU-PROB | 0 | -0.78- 0.78 | ns | >0.99 |
| NPC SZ-HU-MO vs. NPC SZ-HU-PROB | 0 | -0.64- 0.64 | ns | >0.99 |
|  |  |  |  |  |
| **Day 2** |  |  |  |  |
| NPC SZ_HU-FA vs. NPC SZ-HU-MO | 0.051 | -0.73- 0.83 | ns | 0.98 |
| NPC SZ_HU-FA vs. NPC SZ-HU-PROB | 0.024 | -0.76- 0.80 | ns | 0.99 |
| NPC SZ-HU-MO vs. NPC SZ-HU-PROB | -0.027 | -0.66- 0.61 | ns | 0.99 |
|  |  |  |  |  |
| **Day 3** |  |  |  |  |
| NPC SZ_HU-FA vs. NPC SZ-HU-MO | -0.178 | -0.96- 0.60 | ns | 0.8467 |
| NPC SZ_HU-FA vs. NPC SZ-HU-PROB | -0.389 | -1.17- 0.39 | ns | 0.4581 |
| NPC SZ-HU-MO vs. NPC SZ-HU-PROB | -0.211 | -0.85- 0.42 | ns | 0.7066 |
|  |  |  |  |  |
| **Day 4** |  |  |  |  |
| NPC SZ_HU-FA vs. NPC SZ-HU-MO | -0.324 | -1.10-0.45 | ns | 0.579 |
| **NPC SZ_HU-FA vs. NPC SZ-HU-PROB** | **-1.102** | **-1.88--0.31** | ****** | **0.003** |
| **NPC SZ-HU-MO vs. NPC SZ-HU-PROB** | **-0.776** | **-1.41- -0.13** | ***** | **0.013** |

1. **Scratch-test, one-way ANOVA.**

| **ANOVA table (Scratch clone1)** | **SS** | **DF** | **MS** | **F (DFn. DFd)** | **P value** |
| --- | --- | --- | --- | --- | --- |
| **Treatment (between columns)** | **0.0212** | **2** | **0.0106** | **F (2. 10) = 0.7101** | **P=0.514** |
| **Residual (within columns)** | **0.1493** | **10** | **0.01493** |  |  |
| **Total** | **0.1705** | **12** |  |  |  |

| **Table Analyzed** | **Scratch clone2** |
| --- | --- |
|  |  |
| **Column B** | **NPC SZ-HU-PROB** |
| **vs.** | **vs.** |
| **Column A** | **NPC SZ-HU-MO** |
|  |  |
| **Unpaired t test** |  |
| **P value** | **0.1405** |
| **P value summary** | **ns** |
| **Significantly different (P < 0.05)?** | **No** |
| **One- or two-tailed P value?** | **Two-tailed** |
| **t. df** | **t=1.636. df=8** |

| **Anova multiple comparison- sratch (clone1-clone2)** |  |  |  |  |
| --- | --- | --- | --- | --- |
| **Tukey's multiple comparisons test** | **Mean difference** | **95% CI of difference** | **Summary** | **Adjusted p-value** |
| NPC SZ_HU-FA vs. NPC SZ-HU-MO 1 | -0.02109 | -0.2423-0.2001 | ns | 0.99 |
| NPC SZ_HU-FA vs. NPC SZ-HU-PROB 1 | -0.1061 | -0.3678-0.1557 | ns | 0.79 |
| **NPC SZ_HU-FA vs. NPC SZ-HU-MO 2** | 0.2301 | 0.008958-0.4513 | * | **0.03** |
| NPC SZ_HU-FA vs. NPC SZ-HU-PROB 2 | 0.1345 | -0.1078-0.3768 | ns | 0.51 |
| NPC SZ_HU-FA vs. CTRL | -0.01616 | -0.3129-0.2806 | ns | >0.99 |
| NPC SZ-HU-MO 1 vs. NPC SZ-HU-PROB 1 | -0.08497 | -0.3273-0.1573 | ns | 0.87 |
| **NPC SZ-HU-MO 1 vs. NPC SZ-HU-MO 2** | 0.2512 | 0.05340-0.4491 | ** | **0.00** |
| NPC SZ-HU-MO 1 vs. NPC SZ-HU-PROB 2 | 0.1556 | -0.06556-0.3768 | ns | 0.27 |
| NPC SZ-HU-MO 1 vs. CTRL | 0.004925 | -0.2749-0.2847 | ns | >0.99 |
| **NPC SZ-HU-PROB 1 vs. NPC SZ-HU-MO 2** | 0.3362 | 0.09391-0.5785 | ** | **0.003** |
| NPC SZ-HU-PROB 1 vs. NPC SZ-HU-PROB 2 | 0.2406 | -0.02112-0.5023 | ns | 0.08 |
| NPC SZ-HU-PROB 1 vs. CTRL | 0.0899 | -0.2229-0.4027 | ns | 0.93 |
| NPC SZ-HU-MO 2 vs. NPC SZ-HU-PROB 2 | -0.09561 | -0.3168-0.1256 | ns | 0.74 |
| NPC SZ-HU-MO 2 vs. CTRL | -0.2463 | -0.5261-0.03347 | ns | 0.10 |
| NPC SZ-HU-PROB 2 vs. CTRL | -0.1507 | -0.4475-0.1461 | ns | 0.60 |

1. **Neurite outgrowth test, one-way ANOVA.**

| **DMSO condition** |  |  |  |  |  |
| --- | --- | --- | --- | --- | --- |
|  |  |  |  |  |  |
| **Table Analyzed** | **neurite outgrowth DMSO** | | |  |  |
|  |  |  |  |  |  |
| **Two-way ANOVA** | **Ordinary** |  |  |  |  |
| **Alpha** | **0.05** |  |  |  |  |
|  |  |  |  |  |  |
| **Source of Variation** | **% of total variation** | **P value** | **P value summary** | **Significant?** | |
| **Interaction** | **11.12** | **<0.0001** | ******** | **Yes** |  |
| **Row Factor** | **30.09** | **<0.0001** | ******** | **Yes** |  |
| **Column Factor** | **45.64** | **<0.0001** | ******** | **Yes** |  |
|  |  |  |  |  |  |
| **ANOVA table** | **SS** | **DF** | **MS** | **F (DFn. DFd)** | **P value** |
| **Interaction** | **1.7** | **32** | **0.05312** | **F (32. 102) = 2.698** | **P<0.0001** |
| **Row Factor** | **4.598** | **16** | **0.2873** | **F (16. 102) = 14.59** | **P<0.0001** |
| **Column Factor** | **6.974** | **2** | **3.487** | **F (2. 102) = 177.1** | **P<0.0001** |
| **Residual** | **2.008** | **102** | **0.01969** |  |  |

| **DMSO neurite outgrowth** |  |  |  |  |
| --- | --- | --- | --- | --- |
| Within each row. compare columns (simple effects within rows) | | | |  |
|  |  |  |  |  |
| Number of families | 17 |  |  |  |
| Number of comparisons per family | 3 |  |  |  |
| Alpha | 0.05 |  |  |  |
|  |  |  |  |  |
| **Tukey's multiple comparisons test** | **Mean difference** | **95% CI of difference** | **Summary** | **Adjusted p-value** |
|  |  |  |  |  |
| 0.00 |  |  |  |  |
| SZ-HU-PROB 1 vs. SZ-HU-MO 1 | 0 | -0.2725 to 0.2725 | ns | >0.9999 |
| SZ-HU-PROB 1 vs. SZ-HU-FA 1 | 0 | -0.2725 to 0.2725 | ns | >0.9999 |
| SZ-HU-MO 1 vs. SZ-HU-FA 1 | 0 | -0.2725 to 0.2725 | ns | >0.9999 |
|  |  |  |  |  |
| 0.25 |  |  |  |  |
| SZ-HU-PROB 1 vs. SZ-HU-MO 1 | 0.13 | -0.1425 to 0.4025 | ns | 0.4951 |
| SZ-HU-PROB 1 vs. SZ-HU-FA 1 | -0.03 | -0.3025 to 0.2425 | ns | 0.9629 |
| SZ-HU-MO 1 vs. SZ-HU-FA 1 | -0.16 | -0.4325 to 0.1125 | ns | 0.3465 |
|  |  |  |  |  |
| 0.50 |  |  |  |  |
| SZ-HU-PROB 1 vs. SZ-HU-MO 1 | 0.21 | -0.06250 to 0.4825 | ns | 0.1641 |
| SZ-HU-PROB 1 vs. SZ-HU-FA 1 | -0.02 | -0.2925 to 0.2525 | ns | 0.9833 |
| SZ-HU-MO 1 vs. SZ-HU-FA 1 | -0.23 | -0.5025 to 0.04250 | ns | 0.1156 |
|  |  |  |  |  |
| 0.75 |  |  |  |  |
| SZ-HU-PROB 1 vs. SZ-HU-MO 1 | 0.19 | -0.08250 to 0.4625 | ns | 0.2263 |
| SZ-HU-PROB 1 vs. SZ-HU-FA 1 | -0.08 | -0.3525 to 0.1925 | ns | 0.765 |
| SZ-HU-MO 1 vs. SZ-HU-FA 1 | -0.27 | -0.5425 to 0.002500 | ns | 0.0527 |
|  |  |  |  |  |
| 1.00 |  |  |  |  |
| SZ-HU-PROB 1 vs. SZ-HU-MO 1 | 0.25 | -0.02250 to 0.5225 | ns | 0.0791 |
| SZ-HU-PROB 1 vs. SZ-HU-FA 1 | -0.16 | -0.4325 to 0.1125 | ns | 0.3465 |
| SZ-HU-MO 1 vs. SZ-HU-FA 1 | -0.41 | -0.6825 to -0.1375 | ****** | **0.0015** |
|  |  |  |  |  |
| 1.25 |  |  |  |  |
| SZ-HU-PROB 1 vs. SZ-HU-MO 1 | 0.16 | -0.1125 to 0.4325 | ns | 0.3465 |
| SZ-HU-PROB 1 vs. SZ-HU-FA 1 | -0.22 | -0.4925 to 0.05250 | ns | 0.1382 |
| SZ-HU-MO 1 vs. SZ-HU-FA 1 | -0.38 | -0.6525 to -0.1075 | ****** | **0.0036** |
|  |  |  |  |  |
| 1.50 |  |  |  |  |
| SZ-HU-PROB 1 vs. SZ-HU-MO 1 | 0.2 | -0.07250 to 0.4725 | ns | 0.1934 |
| SZ-HU-PROB 1 vs. SZ-HU-FA 1 | -0.17 | -0.4425 to 0.1025 | ns | 0.3029 |
| SZ-HU-MO 1 vs. SZ-HU-FA 1 | -0.37 | -0.6425 to -0.09750 | ****** | **0.0047** |
|  |  |  |  |  |
| 1.75 |  |  |  |  |
| SZ-HU-PROB 1 vs. SZ-HU-MO 1 | 0.25 | -0.02250 to 0.5225 | ns | 0.0791 |
| SZ-HU-PROB 1 vs. SZ-HU-FA 1 | -0.27 | -0.5425 to 0.002500 | ns | 0.0527 |
| SZ-HU-MO 1 vs. SZ-HU-FA 1 | -0.52 | -0.7925 to -0.2475 | ******** | **<0.0001** |
|  |  |  |  |  |
| 2.00 |  |  |  |  |
| SZ-HU-PROB 1 vs. SZ-HU-MO 1 | 0.25 | -0.02250 to 0.5225 | ns | 0.0791 |
| SZ-HU-PROB 1 vs. SZ-HU-FA 1 | -0.29 | -0.5625 to -0.01750 | ***** | **0.0342** |
| SZ-HU-MO 1 vs. SZ-HU-FA 1 | -0.54 | -0.8125 to -0.2675 | ******** | **<0.0001** |
|  |  |  |  |  |
| 2.25 |  |  |  |  |
| SZ-HU-PROB 1 vs. SZ-HU-MO 1 | 0.44 | 0.1675 to 0.7125 | ******* | **0.0006** |
| SZ-HU-PROB 1 vs. SZ-HU-FA 1 | -0.19 | -0.4625 to 0.08250 | ns | 0.2263 |
| SZ-HU-MO 1 vs. SZ-HU-FA 1 | -0.63 | -0.9025 to -0.3575 | ******** | **<0.0001** |
|  |  |  |  |  |
| 2.50 |  |  |  |  |
| SZ-HU-PROB 1 vs. SZ-HU-MO 1 | 0.4 | 0.1275 to 0.6725 | ****** | **0.002** |
| SZ-HU-PROB 1 vs. SZ-HU-FA 1 | -0.23 | -0.5025 to 0.04250 | ns | 0.1156 |
| SZ-HU-MO 1 vs. SZ-HU-FA 1 | -0.63 | -0.9025 to -0.3575 | ******** | **<0.0001** |
|  |  |  |  |  |
| 2.75 |  |  |  |  |
| SZ-HU-PROB 1 vs. SZ-HU-MO 1 | 0.5 | 0.2275 to 0.7725 | ******** | **<0.0001** |
| SZ-HU-PROB 1 vs. SZ-HU-FA 1 | -0.24 | -0.5125 to 0.03250 | ns | 0.096 |
| SZ-HU-MO 1 vs. SZ-HU-FA 1 | -0.74 | -1.012 to -0.4675 | ******** | **<0.0001** |
|  |  |  |  |  |
| 3.00 |  |  |  |  |
| SZ-HU-PROB 1 vs. SZ-HU-MO 1 | 0.45 | 0.1775 to 0.7225 | ******* | **0.0005** |
| SZ-HU-PROB 1 vs. SZ-HU-FA 1 | -0.24 | -0.5125 to 0.03250 | ns | 0.096 |
| SZ-HU-MO 1 vs. SZ-HU-FA 1 | -0.69 | -0.9625 to -0.4175 | ******** | **<0.0001** |
|  |  |  |  |  |
| 3.25 |  |  |  |  |
| SZ-HU-PROB 1 vs. SZ-HU-MO 1 | 0.51 | 0.2375 to 0.7825 | ******** | **<0.0001** |
| SZ-HU-PROB 1 vs. SZ-HU-FA 1 | -0.3 | -0.5725 to -0.02750 | ***** | **0.0273** |
| SZ-HU-MO 1 vs. SZ-HU-FA 1 | -0.81 | -1.082 to -0.5375 | ******** | **<0.0001** |
|  |  |  |  |  |
| 3.50 |  |  |  |  |
| SZ-HU-PROB 1 vs. SZ-HU-MO 1 | 0.45 | 0.1775 to 0.7225 | ******* | **0.0005** |
| SZ-HU-PROB 1 vs. SZ-HU-FA 1 | -0.28 | -0.5525 to -0.007500 | ***** | **0.0426** |
| SZ-HU-MO 1 vs. SZ-HU-FA 1 | -0.73 | -1.002 to -0.4575 | ******** | **<0.0001** |
|  |  |  |  |  |
| 3.75 |  |  |  |  |
| SZ-HU-PROB 1 vs. SZ-HU-MO 1 | 0.55 | 0.2775 to 0.8225 | ******** | **<0.0001** |
| SZ-HU-PROB 1 vs. SZ-HU-FA 1 | -0.29 | -0.5625 to -0.01750 | ***** | **0.0342** |
| SZ-HU-MO 1 vs. SZ-HU-FA 1 | -0.84 | -1.112 to -0.5675 | ******** | **<0.0001** |
|  |  |  |  |  |
| 4.00 |  |  |  |  |
| SZ-HU-PROB 1 vs. SZ-HU-MO 1 | 0.66 | 0.3875 to 0.9325 | ******** | **<0.0001** |
| SZ-HU-PROB 1 vs. SZ-HU-FA 1 | -0.17 | -0.4425 to 0.1025 | ns | 0.3029 |
| SZ-HU-MO 1 vs. SZ-HU-FA 1 | -0.83 | -1.102 to -0.5575 | ******** | **<0.0001** |

| **NBS neurite outgrowth** |  |  |  |  |  |
| --- | --- | --- | --- | --- | --- |
|  |  |  |  |  |  |
| Table Analyzed | Neurite outgrowth NBS | |  |  |  |
|  |  |  |  |  |  |
| Two-way ANOVA | Ordinary |  |  |  |  |
| Alpha | 0.05 |  |  |  |  |
|  |  |  |  |  |  |
| Source of Variation | % of total variation | P value | P value summary | Significant? | |
| Interaction | 1.466 | >0.9999 | **ns** | **No** |  |
| Row Factor | 60.81 | <0.0001 | **** | Yes |  |
| Column Factor | 3.167 | 0.0114 | * | Yes |  |
|  |  |  |  |  |  |
| ANOVA table | SS | DF | MS | F (DFn. DFd) | P value |
| Interaction | 0.4779 | 32 | 0.01493 | F (32. 102) = 0.1352 | P>0.9999 |
| Row Factor | 19.83 | 16 | 1.239 | F (16. 102) = 11.22 | P<0.0001 |
| Column Factor | 1.033 | 2 | **0.5163** | F (2. 102) = 4.674 | P=0.0114 |
| Residual | 11.27 | 102 | 0.1105 |  |  |

| **NBS neurite outgrowth** |  |  |  |  |
| --- | --- | --- | --- | --- |
| Within each row. compare columns (simple effects within rows) | | |  |  |
|  |  |  |  |  |
| Number of families | 17 |  |  |  |
| Number of comparisons per family | 3 |  |  |  |
| Alpha | 0.05 |  |  |  |
|  |  |  |  |  |
| **Tukey's multiple comparisons test** | **Mean difference** | **95% CI of difference** | **Summary** | **Adjusted p-value** |
|  |  |  |  |  |
| 0.00 |  |  |  |  |
| SZ-HU-PROB 1 vs. SZ-HU-MO 1 | 0 | -0.6454 to 0.6454 | ns | >0.9999 |
| SZ-HU-PROB 1 vs. SZ-HU-FA 1 | 0 | -0.6454 to 0.6454 | ns | >0.9999 |
| SZ-HU-MO 1 vs. SZ-HU-FA 1 | 0 | -0.6454 to 0.6454 | ns | >0.9999 |
|  |  |  |  |  |
| 0.25 |  |  |  |  |
| SZ-HU-PROB 1 vs. SZ-HU-MO 1 | -0.14 | -0.7854 to 0.5054 | ns | 0.8638 |
| SZ-HU-PROB 1 vs. SZ-HU-FA 1 | 0.1 | -0.5454 to 0.7454 | ns | 0.9279 |
| SZ-HU-MO 1 vs. SZ-HU-FA 1 | 0.24 | -0.4054 to 0.8854 | ns | 0.6513 |
|  |  |  |  |  |
| 0.50 |  |  |  |  |
| SZ-HU-PROB 1 vs. SZ-HU-MO 1 | -0.28 | -0.9254 to 0.3654 | ns | 0.5586 |
| SZ-HU-PROB 1 vs. SZ-HU-FA 1 | 0.01 | -0.6354 to 0.6554 | ns | 0.9993 |
| SZ-HU-MO 1 vs. SZ-HU-FA 1 | 0.29 | -0.3554 to 0.9354 | ns | 0.5356 |
|  |  |  |  |  |
| 0.75 |  |  |  |  |
| SZ-HU-PROB 1 vs. SZ-HU-MO 1 | -0.15 | -0.7954 to 0.4954 | ns | 0.8453 |
| SZ-HU-PROB 1 vs. SZ-HU-FA 1 | 0.04 | -0.6054 to 0.6854 | ns | 0.9881 |
| SZ-HU-MO 1 vs. SZ-HU-FA 1 | 0.19 | -0.4554 to 0.8354 | ns | 0.7639 |
|  |  |  |  |  |
| 1.00 |  |  |  |  |
| SZ-HU-PROB 1 vs. SZ-HU-MO 1 | -0.16 | -0.8054 to 0.4854 | ns | 0.826 |
| SZ-HU-PROB 1 vs. SZ-HU-FA 1 | -0.01 | -0.6554 to 0.6354 | ns | 0.9993 |
| SZ-HU-MO 1 vs. SZ-HU-FA 1 | 0.15 | -0.4954 to 0.7954 | ns | 0.8453 |
|  |  |  |  |  |
| 1.25 |  |  |  |  |
| SZ-HU-PROB 1 vs. SZ-HU-MO 1 | -0.01 | -0.6554 to 0.6354 | ns | 0.9993 |
| SZ-HU-PROB 1 vs. SZ-HU-FA 1 | 0.07 | -0.5754 to 0.7154 | ns | 0.964 |
| SZ-HU-MO 1 vs. SZ-HU-FA 1 | 0.08 | -0.5654 to 0.7254 | ns | 0.9532 |
|  |  |  |  |  |
| 1.50 |  |  |  |  |
| SZ-HU-PROB 1 vs. SZ-HU-MO 1 | -0.11 | -0.7554 to 0.5354 | ns | 0.9135 |
| SZ-HU-PROB 1 vs. SZ-HU-FA 1 | -0.04 | -0.6854 to 0.6054 | ns | 0.9881 |
| SZ-HU-MO 1 vs. SZ-HU-FA 1 | 0.07 | -0.5754 to 0.7154 | ns | 0.964 |
|  |  |  |  |  |
| 1.75 |  |  |  |  |
| SZ-HU-PROB 1 vs. SZ-HU-MO 1 | -0.11 | -0.7554 to 0.5354 | ns | 0.9135 |
| SZ-HU-PROB 1 vs. SZ-HU-FA 1 | -0.02 | -0.6654 to 0.6254 | ns | 0.997 |
| SZ-HU-MO 1 vs. SZ-HU-FA 1 | 0.09 | -0.5554 to 0.7354 | ns | 0.9412 |
|  |  |  |  |  |
| 2.00 |  |  |  |  |
| SZ-HU-PROB 1 vs. SZ-HU-MO 1 | -0.04 | -0.6854 to 0.6054 | ns | 0.9881 |
| SZ-HU-PROB 1 vs. SZ-HU-FA 1 | 0.05 | -0.5954 to 0.6954 | ns | 0.9815 |
| SZ-HU-MO 1 vs. SZ-HU-FA 1 | 0.09 | -0.5554 to 0.7354 | ns | 0.9412 |
|  |  |  |  |  |
| 2.25 |  |  |  |  |
| SZ-HU-PROB 1 vs. SZ-HU-MO 1 | -0.25 | -0.8954 to 0.3954 | ns | 0.6281 |
| SZ-HU-PROB 1 vs. SZ-HU-FA 1 | -0.14 | -0.7854 to 0.5054 | ns | 0.8638 |
| SZ-HU-MO 1 vs. SZ-HU-FA 1 | 0.11 | -0.5354 to 0.7554 | ns | 0.9135 |
|  |  |  |  |  |
| 2.50 |  |  |  |  |
| SZ-HU-PROB 1 vs. SZ-HU-MO 1 | -0.14 | -0.7854 to 0.5054 | ns | 0.8638 |
| SZ-HU-PROB 1 vs. SZ-HU-FA 1 | -0.09 | -0.7354 to 0.5554 | ns | 0.9412 |
| SZ-HU-MO 1 vs. SZ-HU-FA 1 | 0.05 | -0.5954 to 0.6954 | ns | 0.9815 |
|  |  |  |  |  |
| 2.75 |  |  |  |  |
| SZ-HU-PROB 1 vs. SZ-HU-MO 1 | -0.3 | -0.9454 to 0.3454 | ns | 0.5129 |
| SZ-HU-PROB 1 vs. SZ-HU-FA 1 | -0.1 | -0.7454 to 0.5454 | ns | 0.9279 |
| SZ-HU-MO 1 vs. SZ-HU-FA 1 | 0.2 | -0.4454 to 0.8454 | ns | 0.7421 |
|  |  |  |  |  |
| 3.00 |  |  |  |  |
| SZ-HU-PROB 1 vs. SZ-HU-MO 1 | -0.22 | -0.8654 to 0.4254 | ns | 0.6973 |
| SZ-HU-PROB 1 vs. SZ-HU-FA 1 | -0.14 | -0.7854 to 0.5054 | ns | 0.8638 |
| SZ-HU-MO 1 vs. SZ-HU-FA 1 | 0.08 | -0.5654 to 0.7254 | ns | 0.9532 |
|  |  |  |  |  |
| 3.25 |  |  |  |  |
| SZ-HU-PROB 1 vs. SZ-HU-MO 1 | -0.27 | -0.9154 to 0.3754 | ns | 0.5817 |
| SZ-HU-PROB 1 vs. SZ-HU-FA 1 | -0.03 | -0.6754 to 0.6154 | ns | 0.9933 |
| SZ-HU-MO 1 vs. SZ-HU-FA 1 | 0.24 | -0.4054 to 0.8854 | ns | 0.6513 |
|  |  |  |  |  |
| 3.50 |  |  |  |  |
| SZ-HU-PROB 1 vs. SZ-HU-MO 1 | -0.23 | -0.8754 to 0.4154 | ns | 0.6744 |
| SZ-HU-PROB 1 vs. SZ-HU-FA 1 | -0.08 | -0.7254 to 0.5654 | ns | 0.9532 |
| SZ-HU-MO 1 vs. SZ-HU-FA 1 | 0.15 | -0.4954 to 0.7954 | ns | 0.8453 |
|  |  |  |  |  |
| 3.75 |  |  |  |  |
| SZ-HU-PROB 1 vs. SZ-HU-MO 1 | -0.35 | -0.9954 to 0.2954 | ns | 0.4042 |
| SZ-HU-PROB 1 vs. SZ-HU-FA 1 | -0.05 | -0.6954 to 0.5954 | ns | 0.9815 |
| SZ-HU-MO 1 vs. SZ-HU-FA 1 | 0.3 | -0.3454 to 0.9454 | ns | 0.5129 |
|  |  |  |  |  |
| 4.00 |  |  |  |  |
| SZ-HU-PROB 1 vs. SZ-HU-MO 1 | -0.46 | -1.105 to 0.1854 | ns | 0.212 |
| SZ-HU-PROB 1 vs. SZ-HU-FA 1 | -0.18 | -0.8254 to 0.4654 | ns | 0.7852 |
| SZ-HU-MO 1 vs. SZ-HU-FA 1 | 0.28 | -0.3654 to 0.9254 | ns | 0.5586 |

1. **CellRox-test, one-way ANOVA.**

| ANOVA summary-**CellRox Clone1** |  |  |  |  |  |
| --- | --- | --- | --- | --- | --- |
| F | 1.398 |  |  |  |  |
| P value | 0.3175 |  |  |  |  |
| P value summary | ns |  |  |  |  |
| Significant diff. among means (P < 0.05)? | No |  |  |  |  |
| R square | 0.4114 |  |  |  |  |
|  |  |  |  |  |  |
| Brown-Forsythe test |  |  |  |  |  |
| F (DFn. DFd) |  |  |  |  |  |
| P value |  |  |  |  |  |
| P value summary |  |  |  |  |  |
| Are SDs significantly different (P < 0.05)? |  |  |  |  |  |
|  |  |  |  |  |  |
| Bartlett's test |  |  |  |  |  |
| Bartlett's statistic (corrected) |  |  |  |  |  |
| P value |  |  |  |  |  |
| P value summary |  |  |  |  |  |
| Are SDs significantly different (P < 0.05)? |  |  |  |  |  |
|  |  |  |  |  |  |
| ANOVA table | SS | DF | MS | F (DFn. DFd) | P value |
| Treatment (between columns) | 8401649 | 4 | 2100412 | F (4. 8) = 1.398 | **P=0.3175** |
| Residual (within columns) | 12019433 | 8 | 1502429 |  |  |
| Total | 20421082 | 12 |  |  |  |

| ANOVA summary- **CellRox (all NPCs)** |  |
| --- | --- |
| F | 2.868 |
| **P value** | **0.0734** |
| P value summary | ns |
| Significant diff. among means (P < 0.05)? | No |
| R square | 0.5891 |

| Number of families | 1 |  |  |  |
| --- | --- | --- | --- | --- |
| Number of comparisons per family | 15 |  |  |  |
| Alpha | 0.05 |  |  |  |
|  |  |  |  |  |
| **Tukey's multiple comparisons test** | **Mean difference** | **95% CI of difference** | **Summary** | **Adjusted p-value** |
| SZ_HU-FA vs. SZ-HU-MO 1 | 495.7 | -2989-3980 | ns | 0.9953 |
| SZ_HU-FA vs. SZ-HU-PROB 1 | 1022 | -2095-4139 | ns | 0.8546 |
| SZ_HU-FA vs. SZ-HU-MO 2 | 1938 | -1179-5055 | ns | 0.3324 |
| SZ_HU-FA vs. SZ-HU-PROB 2 | 2080 | -1404-5565 | ns | 0.3699 |
| SZ_HU-FA vs. CTRL | 2992 | -124.6-6109 | ns | 0.0618 |
| SZ-HU-MO 1 vs. SZ-HU-PROB 1 | 526.5 | -2958-4011 | ns | 0.9938 |
| SZ-HU-MO 1 vs. SZ-HU-MO 2 | 1442 | -2042-4927 | ns | 0.7065 |
| SZ-HU-MO 1 vs. SZ-HU-PROB 2 | 1585 | -2233-5402 | ns | 0.7041 |
| SZ-HU-MO 1 vs. CTRL | 2496 | -988.2-5981 | ns | 0.2146 |
| SZ-HU-PROB 1 vs. SZ-HU-MO 2 | 915.8 | -2201-4033 | ns | 0.9007 |
| SZ-HU-PROB 1 vs. SZ-HU-PROB 2 | 1058 | -2426-4543 | ns | 0.8883 |
| SZ-HU-PROB 1 vs. CTRL | 1970 | -1147-5087 | ns | 0.3176 |
| SZ-HU-MO 2 vs. SZ-HU-PROB 2 | 142.4 | -3342-3627 | ns | >0.9999 |
| SZ-HU-MO 2 vs. CTRL | 1054 | -2063-4171 | ns | 0.839 |
| SZ-HU-PROB 2 vs. CTRL | 911.8 | -2573-4396 | ns | 0.9355 |

1. **CoCl_2_ treatment test, one-way ANOVA.**

| Two-way ANOVA-**CoCl_2_ treatment (clone1)** | Ordinary |  |  |  |  |
| --- | --- | --- | --- | --- | --- |
| Alpha | 0.05 |  |  |  |  |
|  |  |  |  |  |  |
| Source of Variation | % of total variation | P value | P value summary | Significant? |  |
| Interaction | 1.392 | 0.6372 | ns | No |  |
| Row Factor | 73.24 | <0.0001 | **** | Yes |  |
| Column Factor | 0.8713 | 0.4554 | ns | No |  |
|  |  |  |  |  |  |
| ANOVA table | SS | DF | MS | F (DFn. DFd) | P value |
| Interaction | 0.05796 | 4 | 0.01449 | F (4. 45) = 0.6392 | **P=0.6372** |
| Row Factor | 3.05 | 2 | 1.525 | F (2. 45) = 67.28 | P<0.0001 |
| Column Factor | 0.03629 | 2 | 0.01814 | F (2. 45) = 0.8004 | P=0.4554 |
| Residual | 1.02 | 45 | 0.02267 |  |  |

| CoCl2 treatment |  |  |  |  |
| --- | --- | --- | --- | --- |
| Within each row. compare columns (simple effects within rows) | |  |  |  |
|  |  |  |  |  |
| Number of families | 3 |  |  |  |
| Number of comparisons per family | 3 |  |  |  |
| Alpha | 0.05 |  |  |  |
|  |  |  |  |  |
| **Tukey's multiple comparisons test** | **Mean difference** | **95% CI of difference** | **Summary** | **Adjusted p-value** |
|  |  |  |  |  |
| **0 µM** |  |  |  |  |
| SZ-HU-PROB 1 vs. SZ-HU-MO 1 | 0 | -0.2107-0.2107 | ns | >0.9999 |
| SZ-HU-PROB 1 vs. SZ-HU-FA 1 | 0 | -0.2107-0.2107 | ns | >0.9999 |
| SZ-HU-MO 1 vs. SZ-HU-FA 1 | 0 | -0.2107-0.2107 | ns | >0.9999 |
|  |  |  |  |  |
| **125 µM** |  |  |  |  |
| SZ-HU-PROB 1 vs. SZ-HU-MO 1 | -0.0831 | -0.2938-0.1276 | ns | 0.6082 |
| SZ-HU-PROB 1 vs. SZ-HU-FA 1 | -0.02633 | -0.2370-0.1843 | ns | 0.9507 |
| SZ-HU-MO 1 vs. SZ-HU-FA 1 | 0.05676 | -0.1539-0.2674 | ns | 0.7916 |
|  |  |  |  |  |
| **250 µM** |  |  |  |  |
| SZ-HU-PROB 1 vs. SZ-HU-MO 1 | 0.003578 | -0.2071-0.2142 | ns | 0.9991 |
| SZ-HU-PROB 1 vs. SZ-HU-FA 1 | 0.1365 | -0.07419-0.3472 | ns | 0.2689 |
| SZ-HU-MO 1 vs. SZ-HU-FA 1 | 0.1329 | -0.07777-0.3436 | ns | 0.2872 |

**f. Mitotracker test, One-way ANOVA**

| Table Analyzed | **Mitotracker clone1** |
| --- | --- |
| Data sets analyzed | A-C |
|  |  |
| ANOVA summary |  |
| F | 3.557 |
| P value | **0.0643** |
| P value summary | ns |
| Significant diff. among means (P < 0.05)? | No |
| R square | 0.3928 |

| Table Analyzed | **MitoTracker all NPCs** |
| --- | --- |
| Data sets analyzed | A-F |
|  |  |
| ANOVA summary |  |
| F | 2.426 |
| P value | **0.0662** |
| P value summary | ns |
| Significant diff. among means (P < 0.05)? | No |
| R square | 0.3453 |

| Multiple comparison - **MitoTracker all NPCs** |  |  |  |  |
| --- | --- | --- | --- | --- |
| **Tukey's multiple comparisons test** | **Mean difference** | **95% CI of difference** | **Summary** | **Adjusted p-value** |
| SZ-HU-FA vs. SZ-HU-MO 1 | -34474 | -233472-164524 | ns | 0.9939 |
| SZ-HU-FA vs. SZ-HU-PROB 1 | 147294 | -51705-346292 | ns | 0.2355 |
| SZ-HU-FA vs. SZ-HU-MO 2 | -23089 | -249658-203481 | ns | 0.9995 |
| SZ-HU-FA vs. SZ-HU-PROB 2 | 65642 | -125844-257128 | ns | 0.8906 |
| SZ-HU-FA vs. CTRL | 65038 | -126448-256525 | ns | 0.8942 |
| SZ-HU-MO 1 vs. SZ-HU-PROB 1 | 181768 | -5850-369385 | ns | 0.0613 |
| SZ-HU-MO 1 vs. SZ-HU-MO 2 | 11385 | -205257-228027 | ns | >0.9999 |
| SZ-HU-MO 1 vs. SZ-HU-PROB 2 | 100116 | -79514-279746 | ns | 0.5274 |
| SZ-HU-MO 1 vs. CTRL | 99512 | -80118-279142 | ns | 0.5337 |
| SZ-HU-PROB 1 vs. SZ-HU-MO 2 | -170382 | -387024-46259 | ns | 0.1839 |
| SZ-HU-PROB 1 vs. SZ-HU-PROB 2 | -81651 | -261281-97979 | ns | 0.7206 |
| SZ-HU-PROB 1 vs. CTRL | -82255 | -261885-97375 | ns | 0.7145 |
| SZ-HU-MO 2 vs. SZ-HU-PROB 2 | 88731 | -121032-298494 | ns | 0.7753 |
| SZ-HU-MO 2 vs. CTRL | 88127 | -121635-297890 | ns | 0.7801 |
| SZ-HU-PROB 2 vs. CTRL | -603.8 | -171874-170667 | ns | >0.9999 |
